# Augmentation of the RNA m6A reader signature is associated with poor survival by enhancing cell proliferation and EMT across cancer types

**DOI:** 10.1038/s12276-022-00795-z

**Published:** 2022-07-06

**Authors:** Jaeik Oh, Chanwoong Hwa, Dongjun Jang, Seungjae Shin, Soo-Jin Lee, Jiwon Kim, Sang Eun Lee, Hae Rim Jung, Yumi Oh, Giyong Jang, Obin Kwon, Joon-Yong An, Sung-Yup Cho

**Affiliations:** 1 Department of Translational Medicine, Seoul National University College of Medicine, Seoul, 03080 Korea; 2 Department of Internal Medicine, Seoul National University Hospital, Seoul, 03080 Korea; 3 School of Biosystem and Biomedical Science, College of Health Science, Korea University, Seoul, 02841 Korea; 4 BK21FOUR R&E Center for Learning Health Systems, Korea University, Seoul, 02841 Korea; 5 Department of Integrated Biomedical and Life Science, Korea University, Seoul, 02841 Korea; 6 Department of Biomedical Sciences, Seoul National University College of Medicine, Seoul, 03080 Korea; 7 Medical Research Center, Genomic Medicine Institute, Seoul National University College of Medicine, Seoul, 03080 Korea; 8 Cancer Research Institute, Seoul National University, Seoul, 03080 Korea

**Keywords:** Cancer genomics, Tumour biomarkers

## Abstract

N^6^-Methyladenosine (m6A) RNA modification plays a critical role in the posttranscriptional regulation of gene expression. Alterations in cellular m6A levels and m6A-related genes have been reported in many cancers, but whether they play oncogenic or tumor-suppressive roles is inconsistent across cancer types. We investigated common features of alterations in m6A modification and m6A-related genes during carcinogenesis by analyzing transcriptome data of 11 solid tumors from The Cancer Genome Atlas database and our in-house gastric cancer cohort. We calculated m6A writer (W), eraser (E), and reader (R) signatures based on corresponding gene expression. Alterations in the W and E signatures varied according to the cancer type, with a strong positive correlation between the W and E signatures in all types. When the patients were divided according to m6A levels estimated by the ratio of the W and E signatures, the prognostic effect of m6A was inconsistent according to the cancer type. The R and especially the R2 signatures (based on the expression of IGF2BPs) were upregulated in all cancers. Patients with a high R2 signature exhibited poor prognosis across types, which was attributed to enrichment of cell cycle- and epithelial–mesenchymal transition-related pathways. Our study demonstrates common features of m6A alterations across cancer types and suggests that targeting m6A R proteins is a promising strategy for cancer treatment.

## Cancer: The role of modifications to RNA

Studying the effects of a chemical modification of messenger RNA molecules (mRNA), which carry genetic information from DNA to the cell’s protein-making machinery, reveals new insights into the role of these modifications in cancer, suggesting potential therapeutic approaches. Researchers in Seoul, South Korea, led by Joon-Yong An at Korea University and Sung-Yup Cho at Seoul National University investigated the most common modifications of mRNA involving methyl groups (CH_3_): addition (‘writing’), having a regulatory effect on the cell (‘reading’) or removal (‘erasing’). The molecular activities involved in reading the modifications were increased in all 11 types of cancer in cancer-sampling databases and their own patient cohort. Changes in writing and erasing of the modifications varied with cancer type. The proteins that mediate the reading responses to RNA methylation are possible targets for new anti-cancer drugs.

## Introduction

Carcinogenesis is associated with the remodeling of gene expression at the genome, epigenome, and transcriptome levels that results in the development of cancer hallmarks^1^. RNA modification is an additional regulatory layer of gene expression, and N^6^-methyladenosine (m6A) is the most abundant internal modification in eukaryotic mRNAs^2^. m6A RNA modification is involved in several steps of posttranscriptional mRNA regulation, such as splicing, transport, translation, and degradation^2^. Writer and eraser genes dynamically regulate intracellular m6A levels. The writer genes, including METTL3, METTL14, and other appendage genes, form a methyltransferase complex that increases global m6A levels^3,4^. Two eraser genes, ALKBH5 and FTO, remove m6A from mRNAs and downregulate total m6A levels^5,6^. m6A-mediated mRNA regulation occurs via reader genes, which directly bind to m6A sites on mRNAs and execute regulatory processes, such as alternative splicing, translation, stabilization, and degradation^7^.

Because m6A modification plays a critical role in mRNA metabolism, the aberrant regulation of m6A levels is closely associated with carcinogenesis^8^. Dysregulation of m6A-related genes, such as m6A writer, eraser, and reader genes, has been reported in several types of cancers, including breast, lung, liver, ovarian, gastric, and pancreatic cancers^8^. However, whether m6A modification and m6A-related genes play oncogenic or tumor suppressive roles differs depending on the tumor type. For example, METTL14, the main writer gene that forms the methyltransferase complex, has been suggested as an oncogene and a tumor suppressor gene in breast cancer and liver cancer, respectively^9,10^. In addition, both m6A writer and eraser genes have been reported as oncogenes in lung cancer^11^, and chemical inhibitors of m6A writer (METTL3) and eraser (FTO) genes have shown similar anticancer effects on acute myeloid leukemia^12,13^. Therefore, the common roles and underlying molecular mechanisms of m6A modification and m6A-related genes in carcinogenesis are still obscure.

To identify the common features of alterations in m6A modification and m6A-related genes during carcinogenesis across cancer types, we analyzed the transcriptome data of 11 solid tumors from The Cancer Genome Atlas (TCGA) database and our in-house RNA sequencing data of a gastric cancer cohort. Because the functions of m6A target genes are determined by alterations in m6A levels and interactions with m6A readers^11^, we estimated the m6A levels and effects of readers by calculating writer (W), eraser (E), and reader (R) gene signatures. Our analyses suggest that an increased R gene signature is a common characteristic of carcinogenesis and is associated with poor patient outcomes across cancer types.

## Materials and methods

### Pan-cancer RNA sequencing data collection and calculation of m6A signatures

Analysis of the m6A gene signature and the relationship between copy number alterations and mRNA expression was accomplished using mRNA expression data [log_2_(TPM + 0.0001)] from the TCGA TARGET GTEx cohort (https://xenabrowser.net/datapages/). A total of 17 m6A regulators were selected, including nine genes in the writer complex (METTL3, METTL14, WTAP, METTL16, RBM15, RBM15B, CBLL1, KIAA1429, and ZC3H13), two erasers (ALKBH5 and FTO), and six readers (YTHDF1, YTHDF2, YTHDF3, IGF2BP1, IGF2BP2, and IGF2BP3). Each W, E, R1, and R2 signature was calculated by the arithmetic means of writers, erasers, YTHDFs, and IGF2BPs. W-E signatures were calculated by subtracting E signatures from W signatures on a log scale, resulting in the ratio of W and E signatures. We computed the Pearson correlation between the W and E signatures and visualized it using the ‘ggplot2’ package (https://ggplot2.tidyverse.org/). Gene set enrichment analysis (GSEA) and CIBERSORT analyses were accomplished using log_2_(RPKM + 1) from the GDC TCGA cohort for each tumor type (https://xenabrowser.net/datapages/). All genes were mapped to Ensembl IDs (60,483 genes).

### Differential gene expression between tumor and normal tissues

All mRNA expression data were extracted from the TCGA TARGET GTEx cohort in UCSC Xena (https://xena.ucsc.edu/). The fold change (FC) in the comparative analysis of tumors and normal tissues was calculated as follows:

$$FC = \frac{{{{{\mathrm{Mean}}}}\,{{{\mathrm{expression}}}}_{{{{\mathrm{tumor}}}}\,{{{\mathrm{tissue}}}}}}}{{{{{\mathrm{Mean}}}}\,{{{\mathrm{expression}}}}_{{{{\mathrm{normal}}}}\,{{{\mathrm{tissue}}}}}}}$$

We generated a heatmap for the FC data using the ‘pheatmap’ package (https://cran.r-project.org/web/packages/pheatmap/index.html). The red and blue colors in the heatmap represent genes that were more highly expressed in tumor and normal tissues, respectively.

### Estimation of m6A signatures in inflammatory tissues, precancerous lesions and cancer tissues

To determine whether alterations in m6A levels and R signatures were associated with carcinogenesis, we analyzed a dataset from the Gene Expression Omnibus (GEO; https://www.ncbi.nlm.nih.gov/geo/; GSE55696)^14^ to compare the expression of m6A-related genes between gastric inflammatory tissues, precancerous lesions and cancer tissues. For the comparison of 4 group means (chronic gastritis, low-grade gastric intraepithelial neoplasia (GIN), high-grade GIN and early adenocarcinoma), one-way analysis of variance (ANOVA) was performed.

### Correlation between copy number alterations (CNAs) and mRNA expression

All mRNA expression data were extracted from the TCGA TARGET GTEx cohort in UCSC Xena (https://xena.ucsc.edu/), and all CNA data were extracted from the TCGA PanCancer Atlas in cBioPortal for Cancer Genomics (https://www.cbioportal.org/). The putative CNA data were in discrete form (−2 = homozygous deletion; −1 = hemizygous deletion; 0 = neutral/no change; 1 = gain; 2 = high-level amplification). The correlation between CNA and mRNA expression levels was evaluated with the eta coefficient test and marked with an asterisk if the coefficient was greater than 0.3 by R language (version 4.1.0, http://www.R-project.org).

### Evaluation of m6A signatures and CNAs in cancer cell lines

The mRNA expression and copy number alteration data of cancer cell lines were obtained from DepMap cellular model expression data (https://depmap.org/portal/download/). Pearson’s correlation analyses and P values were calculated using log_2_(RSEM + 1) values from the Cancer Cell Line Encyclopedia (CCLE) for each tumor type. For the analysis of copy number alterations, we stratified the GISTIC2 score into 3 classes in the same way suggested in the National Cancer Institute Genomic Data Commons (https://docs.gdc.cancer.gov/Data/Bioinformatics_Pipelines/CNV_Pipeline/; deletion = GISTIC2 score below −0.3, diploid = GISTIC2 score between −0.3 and 0.3, and amplification = GISTIC2 score above 0.3). The correlation between CNA and mRNA expression levels was evaluated with the eta coefficient test and marked with an asterisk if the coefficient was greater than 0.3 by R language (version 4.1.0, http://www.R-project.org).

### Survival analysis according to the m6A gene signature

Survival data were extracted from TCGA and TARGET Pan-Cancer (PANCAN). Because the average levels and distribution of the m6A signature were diverse among the cancer types and datasets, we applied maximally selected rank statistics with several p value approximations using R (‘maxstat’ package) to discriminate tumor samples into two groups for cutoff optimization in the m6A signature^15^ (https://cran.r-project.org/web/packages/maxstat/index.html). The difference in survival between the high and low signature groups was calculated by the Kaplan–Meier method with a two-sided log rank test, and the CondMC method was used to estimate P values. A Kaplan–Meier plot was used to estimate the median overall survival of each group with the ‘survival’ (https://cran.r-project.org/web/packages/survival/index.html) and ‘survminer’ packages (https://cran.r-project.org/web/packages/survminer/index.html).

For validation datasets, we screened datasets from GEO under the following conditions. First, being exclusive from TCGA data, the dataset should provide RNA expression in the form of microarray analysis. Second, since 17 m6A-related genes were used for further evaluation, the microarray chip set should cover all 17 m6A-related genes. Third, clinical data should include patient survival data. Finally, to perform meaningful statistical analysis, at least 20 samples were required in each group. After screening, we used GSE65194 (breast cancer)^16^, GSE38832 (colon adenocarcinoma)^17^, GSE7696 (glioblastoma multiforme)^18^, GSE31210 (lung adenocarcinoma)^19^, GSE157010 (lung squamous cell carcinoma)^20^, GSE26193 (ovarian epithelial cancer)^21^, and GSE15459 (stomach adenocarcinoma)^22^. Each Affymetrix dataset was background-adjusted and normalized by the Robust Multichip Average (RMA) algorithm in the ‘Affy’ package using R language (version 4.1.0, http://www.R-project.org)^23^. The difference in survival between the high and low signature groups based on the median value was calculated by the Kaplan–Meier method with a two-sided log rank test.

### Multivariate Cox regression

Phenotypic data were extracted from TCGA PANCAN in UCSC Xena (https://xena.ucsc.edu/). Major covariates (sex, age, stage, and numbers of immune cells) were considered for multivariate analysis. The numbers of immune cells were estimated by the CIBERSORT package (https://cibersortx.stanford.edu/). Glioblastoma stage and uterine and ovarian cancer sex were not considered. The ‘Coxph’ function was used to estimate beta coefficients. The Cox hazard ratio was plotted with the ‘ggforest’ function.

### Gene set enrichment analysis

GSEA was performed to elucidate the biological pathways that were associated with the altered gene signature. The software and data were downloaded from the GSEA website (https://www.gsea-msigdb.org/gsea/index.jsp). Hallmark gene sets and normalized P values were used to investigate the enriched gene set in the high or low signature groups. Each cell of heatmaps stands for -log_10_(P value), and Euclidean distance was used for clustering.

### Profiling immune cell proportions in tumor tissues

The CIBERSORT package was used to explore the degree of immune cell infiltration by assessing the proportions of 22 immune cell subtypes with the LM22 matrix and TCGA samples. The analysis was performed in absolute mode on the CIBERSORTx website (https://cibersortx.stanford.edu/). Student’s *t* test was used to compare the differences between the high and low signature groups.

### Gastric cancer patient sample collection and RNA sequencing

This study was approved by the Institutional Review Board of the Seoul National University Hospital (No. C-1402-054-555) in accordance with the Declaration of Helsinki. All samples were obtained with informed consent at the Seoul National University Hospital. Samples of gastric cancer tissues and normal gastric tissues were obtained from individuals who underwent gastrectomy at Seoul National University Hospital between 2014 and 2017. RNA extraction from tissues was performed using TRIzol™ (Invitrogen). Samples with an RNA integrity number greater than five were further processed. The 101-bp paired-end libraries were constructed with the TruSeq RNA Sample Prep Kit v2 (Illumina) using 1 µg of RNA. Whole-transcriptome sequencing was performed on an Illumina HiSeq 2000 instrument. Raw FASTQ files were aligned with the human reference genome (GRCh37), and sequenced reads were aligned using a STAR aligner^24^. The sorting and marking of duplicates were performed by Picard tools. After processing the binary aligned and mapping (BAM) files, gene expression levels were quantified by fragments per kilobase of exon per million mapped reads (FPKM). The sequencing data have been deposited in the European Nucleotide Archive (ENA) repository under accession code PRJEB40936.

### Cancer single-cell RNA sequencing data collection and calculation of m6A signatures

To assess the cell-type specificity underlying m6A signatures, we analyzed single-cell RNA sequencing (scRNA-seq) datasets publicly available in the GEO database: breast cancer (GSE180286)^25^, non-small-cell lung cancer (GSE148071)^26^, gastric cancer (GSE183904)^27^, and renal cancer (GSE159115)^28^. From these, we obtained the feature-barcode matrices of tumor or normal samples for each cancer type. To exclude low-quality cells and genes, we followed the same filtering criteria from the original studies as to the number of genes per cell, the percentage of mitochondrial gene expression and unique molecular identifier (UMI) counts. For each cell, the raw gene expression counts were normalized by the total UMI count and log-transformed using the NormalizeData function of the ‘Seurat’ package. To identify the cell-type clusters, we integrated the scRNA-seq dataset of individual patients into the single dataset for each cancer type using Harmony^29^ and performed dimension reduction and clustering using the ‘Seurat’ package^30^. The identified clusters were annotated using the cell type markers listed in the original papers. After removing cells with zero expression, we calculated arithmetic means of the m6A signature genes and evaluated statistical significance for the group difference in gene expression using the ‘ggpubr’ (https://rpkgs.datanovia.com/ggpubr/) and ‘rstatix’ packages (https://rpkgs.datanovia.com/rstatix/).

## Results

### Alterations in m6A writer, eraser, and reader signatures across cancer types

**Fig. 1:**
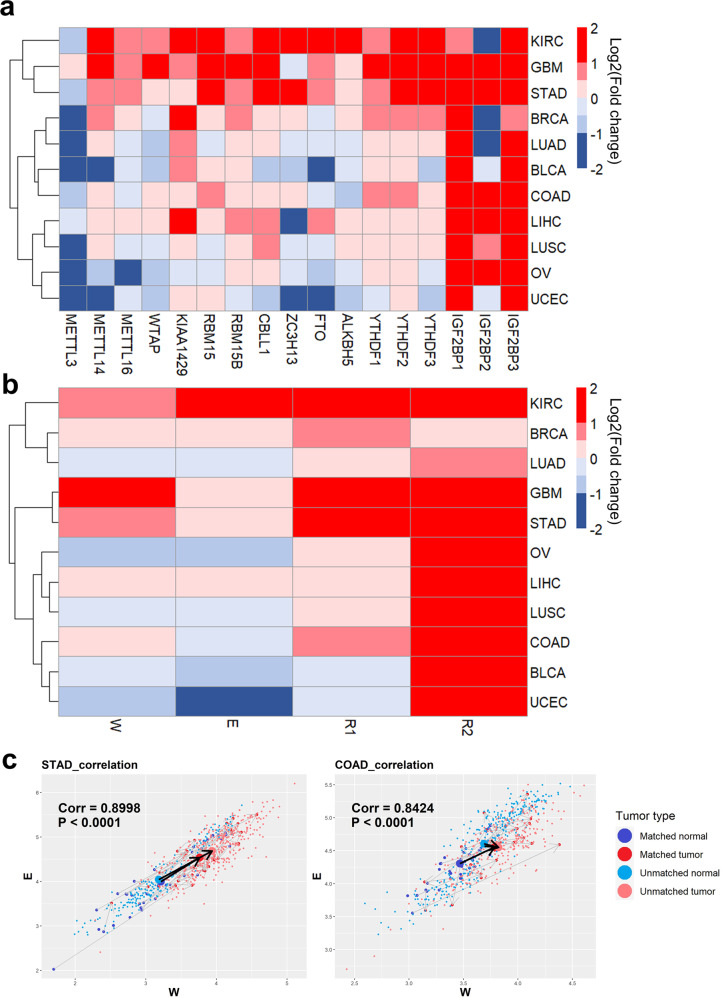
Alterations in m6A writer, eraser, and reader gene signatures across cancer types. **a** Heatmap of alterations in m6A-related gene expression in 11 cancer types. Each column represents an individual m6A-related gene, and each row denotes a cancer type. Colors show the fold change in gene expression in cancer tissues compared to normal tissues from the TCGA database. Key: KIRC, kidney renal clear cell carcinoma; GBM, glioblastoma multiforme; STAD, stomach adenocarcinoma; BRCA, breast invasive carcinoma; LUAD, lung adenocarcinoma; BLCA, bladder urothelial carcinoma; COAD, colon adenocarcinoma; LIHC, liver hepatocellular carcinoma; LUSC, lung squamous cell carcinoma; OV, ovarian serous cystadenocarcinoma; UCEC, uterine corpus endometrial carcinoma. **b** Heatmap of alterations in m6A writer (W), eraser (E), and reader (R) gene signatures in 11 cancer types. Each column represents an individual m6A gene signature, and each row denotes a cancer type. Colors show the fold change in the gene signature in cancer tissues compared to normal tissues from the TCGA database. **c** Scatter plots of the m6A W and E signatures in log scales. Blue dots are normal tissue samples adjacent to the tumor samples, which are marked as red dots. Sky blue and pink dots are normal tissue and tumor samples, respectively, that are not matched. Pearson correlation coefficients (Corr) and *P* values are written on the plot.

To investigate the alterations in RNA m6A modification during carcinogenesis across cancer types, we compared the expression of m6A-related genes between normal and cancer tissues in 11 solid cancer types by analyzing the TCGA database using the USCS Xena platform (https://xenabrowser.net/datapages/)^31^. In the case of m6A writer genes, the expression of METTL3, which is a catalytic subunit in the m6A methyltransferase complex^32^, is decreased in most cancer types compared with normal tissues. This decrease was especially notable in breast invasive carcinoma (BRCA), lung adenocarcinoma (LUAD), bladder urothelial carcinoma (BLCA), lung squamous cell carcinoma (LUSC), ovarian serous cystadenocarcinoma (OV), and uterine corpus endometrial carcinoma (UCEC; Fig. 1a). In contrast, the expression of METTL14 was upregulated in most cancer types, except BLCA, OV, and UCEC (Fig. 1a). The expression of other subunits in the m6A methyltransferase complex and m6A eraser genes varied according to the cancer type (Fig. 1a). However, the expression levels of m6A reader genes in cancer tissues were higher than those in normal tissues regardless of the cancer type (Fig. 1a). Interestingly, the expression of IGF2BP1 and IGF2BP3 increased more than twofold in most cancer types (Fig. 1a). Overall, kidney renal papillary cell carcinoma (KIRC), glioblastoma multiforme (GBM), stomach adenocarcinoma (STAD), and BRCA showed increases in writer, eraser, and reader gene expression. In contrast, LUSC, OV, and UCEC showed downregulation of writer and eraser gene expression.

Total cellular RNA m6A levels are dynamically regulated through the interactions of m6A writer, eraser, and reader proteins. To understand the alteration in overall m6A levels during cancer development, we estimated the m6A W, E, R1 and R2 signatures based on the expression of writer-associated genes (METTL3, METTL14, METTL16, WTAP, KIAA1429, RBM15, RBM15B, CBLL1, and ZC3H13), eraser-associated genes (FTO and ALKBH5), and reader-associated genes (R1: YTHDF1, 2, and 3; R2: IGF2BP1, 2, and 3). Interestingly, the W signature levels were generally associated with the E signature levels (Fig. 1b). For example, compared with normal tissues, KIRC, BRCA, GBM, and STAD displayed higher values in both the W and E signatures simultaneously. In contrast, compared with normal tissues, OV, LUSC, BLCA, and UCEC tissues exhibited a concurrent decrease in the W and E signatures (Fig. 1b). The scatter plot results show that each sample had a strong positive correlation between the W and E signatures across cancer types (Fig. 1c and Supplementary Fig. 1). The only exception, colon adenocarcinoma (COAD), which had a higher W signature and a lower E signature than normal tissues (Fig. 1b), had a similar inclination when unpaired samples were excluded and paired normal and cancer samples from the same patient were examined (Fig. 1c). The positive correlation between the W and E signatures was reproduced in the cancer cell line data of 10 cancer types from Cancer Cell Line Encyclopedia (CCLE; https://sites.broadinstitute.org/ccle/) except liver hepatocellular carcinoma (LIHC) (*R* = 0.38, *P* = 0.078; Supplementary Fig. 2). These data suggest that changes in writer and eraser expression can cancel each other out and sustain homeostasis at the cellular m6A level.

As expected for the R signatures, the R1 (YTHDFs) signature showed higher expression in most cancers than in normal tissues (Fig. 1b), with the exceptions of BLCA and UCEC (Fig. 1b). Intriguingly, the R2 (IGF2BPs) signature was significantly increased in all 11 cancer types compared with normal tissues (*P* < 0.05; Fig. 1b). In addition, compared to chronic gastritis, which is normal gastric tissue with high infiltration of inflammatory cells, the R1 signature was significantly increased in high-grade intraepithelial neoplasia (HGIN), and the R2 signature was significantly increased in HGIN and early-stage gastric carcinoma (Supplementary Fig. 3). Therefore, augmentation of the R signature is a common characteristic across cancer types.

### Copy number amplification is associated with increased expression of R signature genes across cancer types

**Fig. 2:**
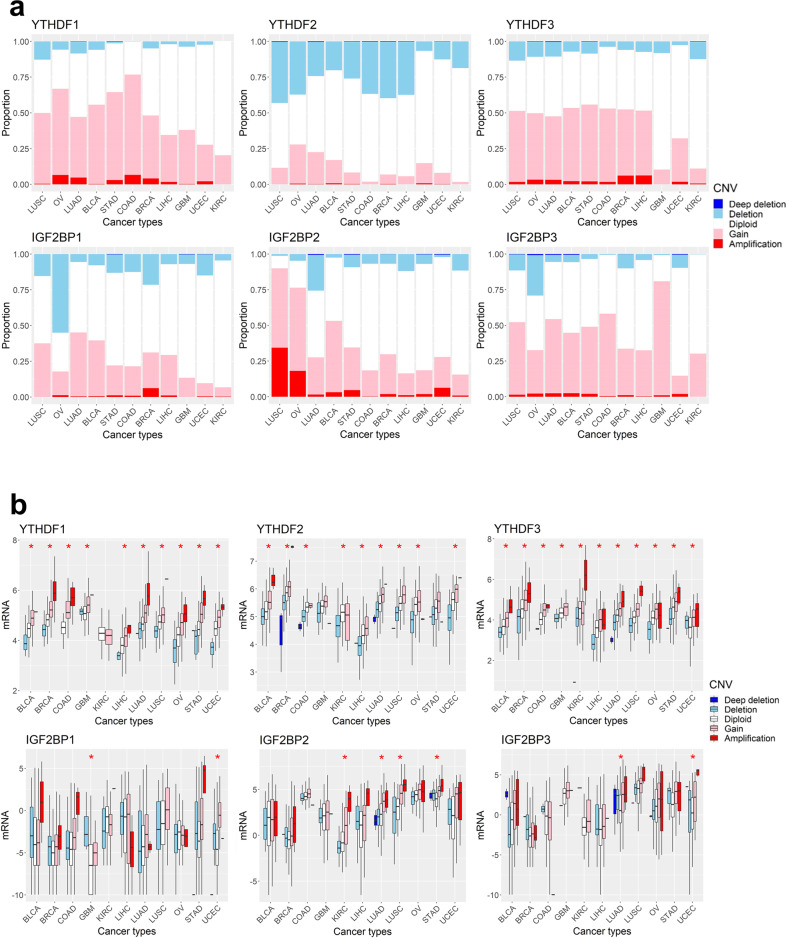
Copy number alterations in m6A reader genes across cancer types. **a** The proportion of patients with copy number alterations in m6A reader genes. From blue to red, each color represents ‘deep deletion’, ‘deletion’, ‘diploid’, ‘gain’, and ‘amplification’. The data were extracted from the putative GISTIC copy number variation (CNV) in cBioPortal (https://www.cbioportal.org/). **b** Correlation between copy number alterations and mRNA expression of m6A reader genes. mRNA expression is demonstrated on a log scale. The asterisk marks data for which the eta coefficient between mRNA expression and CNV is >0.3.

Next, we investigated whether the increased m6A R signature in cancers can be attributed to the alteration in copy numbers in m6A R signature genes. According to the TCGA data analyzed by Genomic Identification of Significant Targets in Cancer (GISTIC)^33^, more than half of the tumor samples showed copy number alterations in R signature genes (Fig. 2a). There were more frequent amplifications than deletions in all 11 types of tumors. Of note are the increases in copy numbers detected in YTHDF1 and 3 and IGF2BP2 and 3 (Fig. 2a). These data are consistent with a those of a previous study that analyzed TCGA data with GISTIC copy number alterations in 33 cancer types^34^. LUSC, OV, LUAD, and BRCA showed a higher proportion of copy number amplifications in R signature genes than other cancer types (Fig. 2a). Copy number amplifications of R signature genes were also observed in cancer cell line data from CCLE (Supplementary Fig. 4a). Observing the relationship between copy number alterations and gene expression, the expression of R1 and R2 signature genes showed positive linear associations with copy number alterations (Fig. 2b and Supplementary Fig. 4b), suggesting that the increase in the R signature is partly due to copy number amplifications of the reader genes.

For writer and eraser genes, analysis of copy number alterations exhibited a larger number of deletions than amplifications, except for KIAA1429 and CBLL1, which showed more amplifications than deletions (Supplementary Fig. 5). The mRNA expression levels of W and E signature genes were positively correlated with copy number alterations in most cancer types (Supplementary Fig. 6). These data suggest that the copy number alterations and expression of W and E signature genes were heterogeneous according to the gene and cancer type.

### Prognostic effect of RNA m6A levels across cancer types

**Fig. 3:**
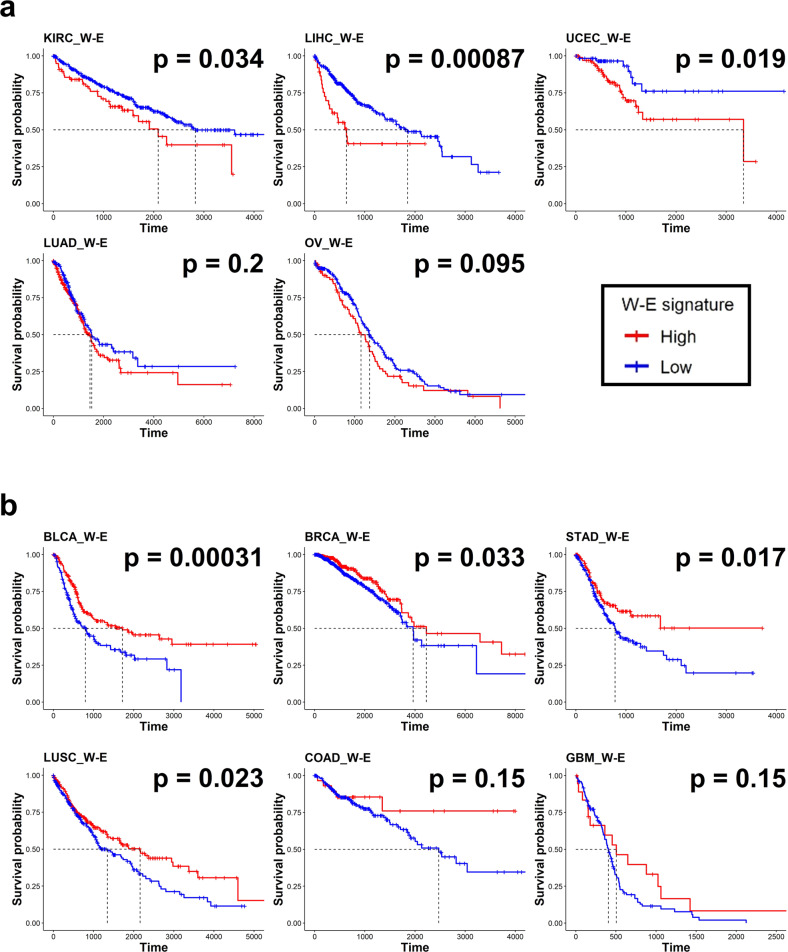
Survival analysis according to the m6A W-E signature across cancer types. **a**, **b** Kaplan–Meier plots for the overall survival of patients with high and low W-E signatures. Red and blue lines represent samples with high and low W-E signatures, respectively. Each median survival and *P* value, determined by the log rank test, is shown. Tumors that have worse survival in high W-E signatures are shown in **a**, and tumors that have favorable survival in high W-E signatures are shown in **b**.

Total cellular RNA m6A levels are regulated by the counteracting actions of writer and eraser proteins. To investigate the effect of m6A levels on cancer patient prognosis, we assumed that RNA m6A levels can be estimated by the ratio of the W and E signatures. The W-E signature represents the ratio of writer and eraser gene expression on a log scale. A high W-E signature means a high W signature and a low E signature, which also represents samples with high m6A levels. A low W-E signature means the opposite. The effect of the W-E signature on overall survival was diverse depending on the tumor type (Fig. 3a, b). KIRC, LIHC, and UCEC patients with a high W-E signature had significantly worse overall survival than patients with a low W-E signature (Fig. 3a). Patients with LUAD and OV showed the same tendency but without statistical significance (Fig. 3a). Consistent with these data, UCEC patients with a high W-E signature were highly associated with an advanced stage of disease, and KIRC and LIHC patients showed a similar but nonsignificant tendency (Supplementary Fig. 7). Multivariate analysis considering age, sex, disease stage, and numbers of immune cells showed that OV patients with a high W-E signature had a worse prognosis (Supplementary Fig. 8a). In the validation cohorts, LUAD and OV patients with a high W-E signature showed a tendency toward poor prognosis (Supplementary Fig. 9a).

In contrast, BLCA, BRCA, STAD, and LUSC patients with a high W-E signature had significantly longer survival times than those with a lower W-E signature (Fig. 3b). Patients with COAD and GBM showed a similar tendency, but the P values were greater than 0.05. Among these cancer types, BLCA, BRCA, and STAD patients with a low W-E signature tended to more frequently have metastasis or locally advanced disease stages (stage III or IV) than patients with a high W-E signature (Supplementary Fig. 7). In multivariate analysis, a low W-E signature was an independent prognostic factor associated with poor prognosis in BLCA and STAD patients (Supplementary Fig. 8b). In the validation cohorts, BLCA, BRCA, STAD, LUSC, and GBM patients with a high W-E signature showed a tendency toward better prognosis (Supplementary Fig. 9b). These data suggest that total RNA m6A levels have different associations with prognosis depending on the cancer type.

### Differential prognostic effect of m6A levels is associated with epithelial–mesenchymal transition and immune cell infiltration

**Fig. 4:**
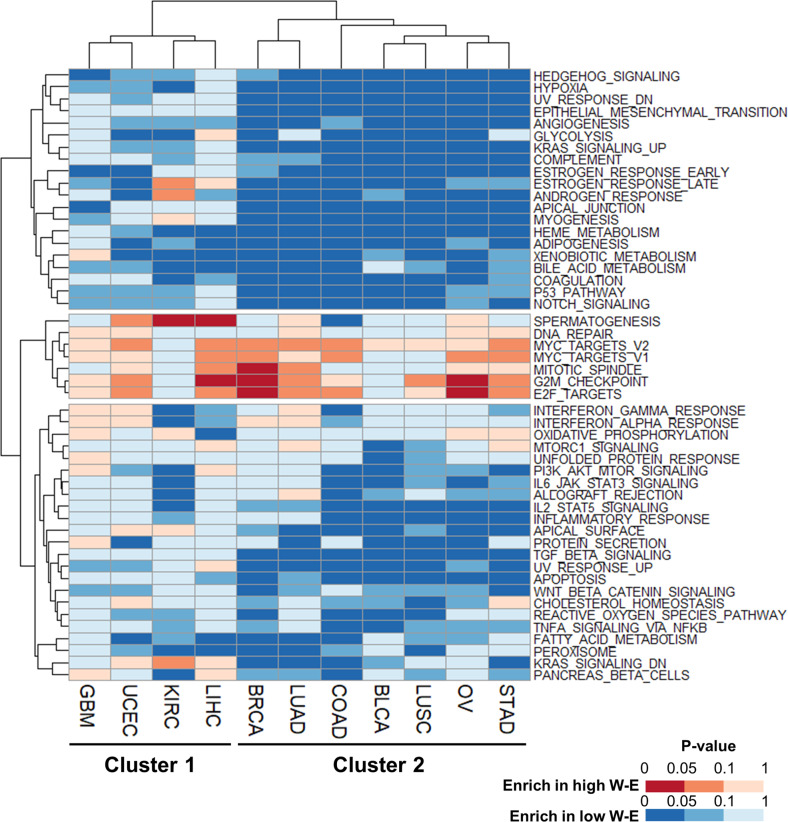
Heatmap of gene set enrichment analysis (GSEA) between patients with high and low W-E signatures. Each column represents an individual cancer type, and each row denotes an enriched hallmark gene set from the Molecular Signatures Database (https://www.gsea-msigdb.org/gsea/msigdb/index.jsp). Gene sets that were enriched in the high W-E signature group are colored red, and gene sets that were enriched in the low W-E signature group are colored blue. The lower the *P* value is, the deeper the color that is shown.

To understand the biological roles of RNA m6A levels across cancer types, we performed GSEA by comparing patients with high and low W-E signatures. When hallmark gene sets from the Molecular Signatures Database (https://www.gsea-msigdb.org/gsea/msigdb/index.jsp) were sorted according to P values, 11 tumor types were divided into two clusters (Fig. 4). The cell cycle-related gene sets (e.g., G2/M checkpoint, mitotic spindle, E2F targets, and MYC targets) were commonly enriched in both clusters with few exceptions (Fig. 4 and Supplementary Fig. 10). Cluster 1 (GBM, UCEC, KIRC, and LIHC), with the exception of GBM, showed poorer survival in patients with high W-E signatures (Fig. 3a). Most of Cluster 2 (COAD, STAD, LUAD, LUSC, BRCA, BLCA, and OV) showed the opposite phenotype (Fig. 3b). The difference between these two clusters was whether the gene sets with epithelial–mesenchymal transition (EMT) and multiple pathways in immune reactions (including inflammatory response, IL2-STAT5 signaling, IL6-JAK-STAT3 signaling, and TGF-β signaling) were significantly enriched (Fig. 4). The association of a more advanced stage and poorer overall survival with a low W-E signature in most Cluster 2 cancer types (Fig. 3b) is probably attributed to the enrichment of EMT and TGF-β signaling in patients with a low W-E signature (Fig. 4 and Supplementary Fig. 11a, b) because these pathways have been associated with poor prognosis in several cancer types^35–37^. In addition, the expression of characteristic markers of EMT (VIM, CHD2, and FN1)^38^ was negatively correlated with the W-E signature in all cancer types of Cluster 2 (Supplementary Fig. 11c, d).

Multiple signaling pathways in immune reactions, such as the IL6, TNFα, and IL2 pathways, were commonly downregulated in Cluster 2 patients with a high W-E signature (Fig. 4). Because these pathways critically regulate the overall immune reaction of the tumor microenvironment (TME), we can speculate that tumors in Cluster 2 have a negative correlation between the W-E signature and immune cell infiltration. To estimate immune cell infiltration in the TME, we performed CIBERSORTx analysis with the LM22 matrix in absolute mode. The first difference between clusters was that Cluster 2, but not Cluster 1, had a higher immune cell count in low W-E signatures (Supplementary Fig. 12). Specifically, Cluster 2 showed enrichment of M2 macrophages, mast cells, and dendritic cells in patients with low W-E signatures. High expression levels of IL-10 and TGF-β, like M2 macrophages, are known to downregulate the immune response and promote metastasis and angiogenesis^39^. Because M2 macrophages are induced by IL-6/STAT3 signals^40^, patients with a low W-E signature in Cluster 2 tumors (BLCA, BRCA, COAD, LUSC, and STAD) had more M2 macrophages in tumor tissues, which is associated with poor prognosis. In contrast, Cluster 1 showed enrichment of eosinophils and M0 macrophages in patients with a high W-E signature. Because eosinophils are known to modulate tumor progression in some tumor types^41^, the enrichment of eosinophils in Cluster 1 tumors may explain the faster cancer progression and poorer survival of patients with a high W-E signature. Therefore, the diverse effects of m6A levels on patient prognosis are highly associated with the immune cell profile in the TME.

### An increased R signature is associated with poor prognosis across cancer types

**Fig. 5:**
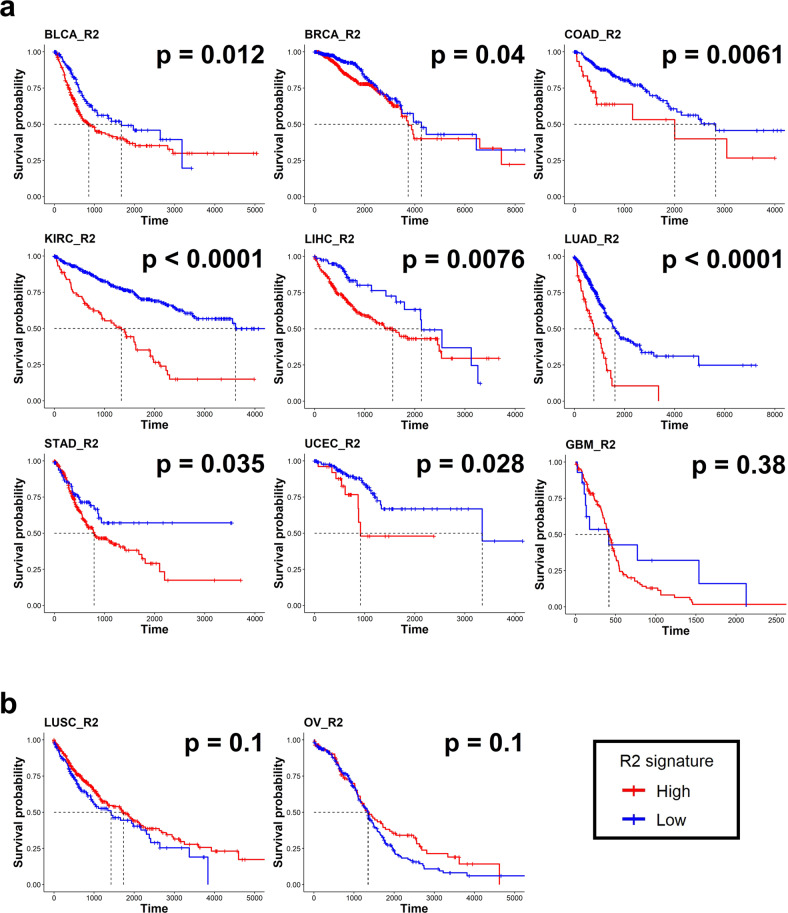
Survival analysis according to the m6A R2 signature across cancer types. **a**, **b** Kaplan–Meier plots for the overall survival of patients with high and low R2 signatures. Red and blue lines represent samples with high and low R2 signatures, respectively. Each median survival and *P* value, determined by the log rank test, is shown. Tumors that have worse survival in the high R2 signature group are shown in **a**, and tumors that do not show a significant difference between the high and low R2 signature groups are shown in **b**.

We next explored the effect of the increased m6A reader signature on patient prognosis by dividing the patients with each cancer type into two groups according to the level of the R1 or R2 signature. We found that a high R1 signature was correlated with a worse prognosis for patients with BRCA, LUSC, LIHC, and UCEC and a better prognosis for patients with BLCA, COAD, KIRC, and STAD. The R1 signature did not significantly alter the overall survival of patients with GBM, LUAD, or OV (Supplementary Fig. 13). When the patients were divided into two groups based on the R2 signature, those with a high R2 signature exhibited a significantly worse prognosis in most cancer types, except LUSC, OV and GBM (Fig. 5). Associations between a high R2 signature and poor prognosis were also observed in the validation cohorts of BLCA, BRCA, COAD, LUAD, STAD, and OV (Supplementary Fig. 14). These results are in accordance with those of previous studies indicating that IGF2BPs play roles in oncogenesis and regulate metastasis^42^.

Multivariate analyses with covariates, including sex, age, stage, and numbers of immune cells predicted by CIBERSORT, indicated that patients with a high R2 signature had a worse prognosis than patients with a low R2 signature in BLCA, BRCA, KIRC, LIHC, LUAD, and STAD (Supplementary Fig. 15). LUSC was the only cancer type in which patients with a high R2 signature showed a better prognosis. In addition, in BLCA, KIRC, and UCEC patients, a high R2 signature was significantly associated with advanced stages of disease (Supplementary Fig. 16). In summary, after excluding the covariate effects, these results indicate that an increased R2 signature is a significant unfavorable factor for patient survival in most cancer types.

### An increased R signature is associated with enrichment of cell cycle- and epithelial–mesenchymal transition (EMT)-related gene sets across cancer types

**Fig. 6:**
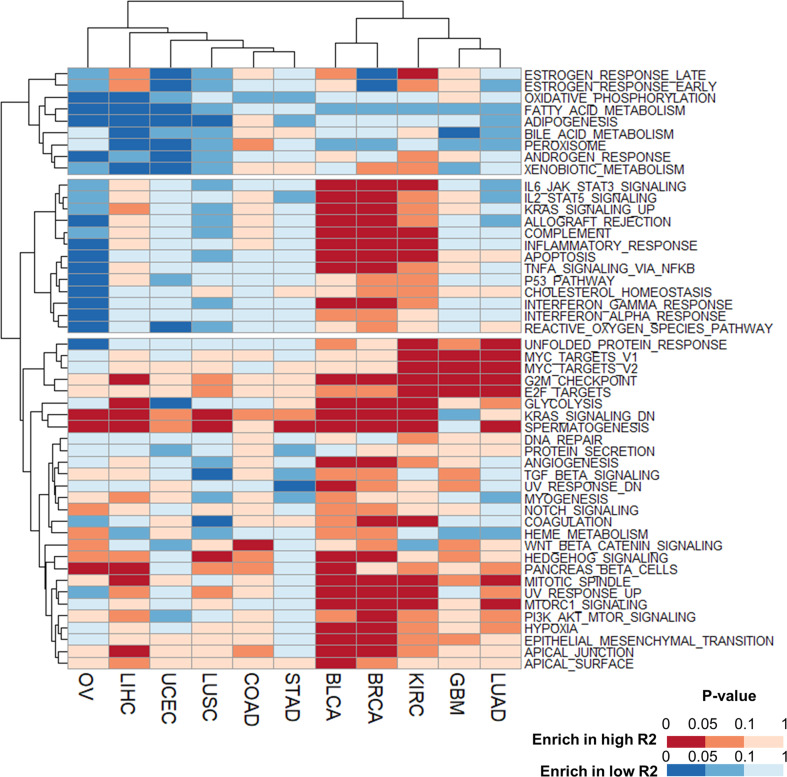
Heatmap of gene set enrichment analysis (GSEA) between patients with high and low R2 signatures. Each column represents an individual cancer type, and each row denotes an enriched hallmark gene set from the Molecular Signatures Database (https://www.gsea-msigdb.org/gsea/msigdb/index.jsp). Gene sets enriched in the high R2 signature group are colored red, and gene sets enriched in the low R2 signature group are colored blue. The lower the *P* value is, the deeper the color that is shown.

IGF2BPs have been suggested to have oncogenic roles, and they are highly expressed from embryonic to solid and hematological malignancies^43^. In light of our results showing the detrimental effect of the R2 signature on patient survival, we explored the underlying molecular mechanisms of IGF2BPs on patient prognosis. Patients with high and low R2 signatures in each cancer type were divided so that the maximum survival differences between the two groups in each cancer type could be revealed by performing GSEA with hallmark gene sets. Several gene sets were commonly enriched in the high R2 signature groups, including G2/M checkpoint, E2F targets, and MYC targets (Fig. 6 and Supplementary Fig. 17). The enrichment of gene sets of the G2/M checkpoint, E2F targets, and MYC targets in samples with a high R2 signature was also observed in the gene expression data of cancer cell lines from CCLE (Supplementary Fig. 18). These gene sets are highly associated with the cell cycle and oncogenesis. MYC itself and several components in the proliferative signaling pathway have been suggested as regulatory targets of IGF2BPs^44,45^.

EMT is strongly related to higher stages and poorer prognosis in many tumors^46^. The EMT gene set was enriched in the high R2 signature group (Fig. 6 and Supplementary Fig. 19). In addition, several EMT-related gene sets, including ‘hypoxia’, ‘apical junction’, and ‘Wnt beta catenin signaling’, were enriched in the high R2 signature group across the cancer types (Fig. 6). The data showing the tendency of patients with a high R2 signature to have more advanced cancer stages and worse overall survival than patients with a lower R2 signature (Supplementary Fig. 16) suggest that the enrichment of EMT-related gene sets is one of the molecular mechanisms associated with poor prognosis in the high R2 signature group.

Among several gene sets associated with the R2 signature, the ‘estrogen response late’ and ‘estrogen response early’ gene sets were negatively correlated with the R2 signature in BRCA, UCEC, and OV (Fig. 6 and Supplementary Fig. 20a, b). These tumor types are related to hormone receptors. The phenomenon that higher R2 signature groups expressed fewer estrogen and progesterone receptors suggests that the R2 signature is highly associated with triple-negative breast cancer. These data are compatible with those of previous studies showing that high R2 signature genes, especially IGF2BP1, induce negative expression of hormone receptors and result in the development of triple-negative breast cancer^47,48^. In cases of ovarian cancer, higher expression of IGF2BPs is associated with a tendency to lose estrogen receptor expression and is associated with higher grade, stage and poorer survival^49,50^. Therefore, our data suggest that IGF2BPs regulate hormone responses in breast and gynecologic cancers, which gives an additional stage-independent risk to patients.

The enrichment of immunologic gene sets, such as IL2-STAT5 signaling, IL6-JAK-STAT3 signaling, and inflammatory responses, varied across the cancer types, resulting in two tumor clusters (Fig. 6). BRCA, BLCA, KIRC, and LIHC showed high enrichment of immunologic gene sets with a high R2 signature, and OV, UCEC, LUSC, and LUAD exhibited the opposite feature with a high R2 signature (Fig. 6 and Supplementary Fig. 21). To investigate the relationship between the enrichment of immunologic gene sets and immune cell infiltration, we performed CIBERSORTx analyses in absolute mode and Student’s *t* test to find differences in immune cell proportions in each cancer type. KIRC, BLCA, and BRCA showed common enrichment of most immune cell types except naïve CD4 T cells (Supplementary Fig. 22). On the other hand, LUSC, LUAD, UCEC, and OV were commonly deficient in immune cells (Supplementary Fig. 22). Therefore, the difference in immune cell infiltration may partially explain the different enrichment of immune gene sets between the two tumor clusters.

### Validation of the effect of the W-E and R2 signatures in the gastric cancer cohort

To validate the alterations in the W-E and R2 signatures and their associations with hallmark gene sets in the TCGA database, we analyzed in-house RNA sequencing data from a gastric cancer cohort. The gastric cancer cohort was established in Seoul National University Hospital and consists of 63 primary tumor samples and 40 normal tissue samples corresponding to primary tumor samples.

**Fig. 7:**
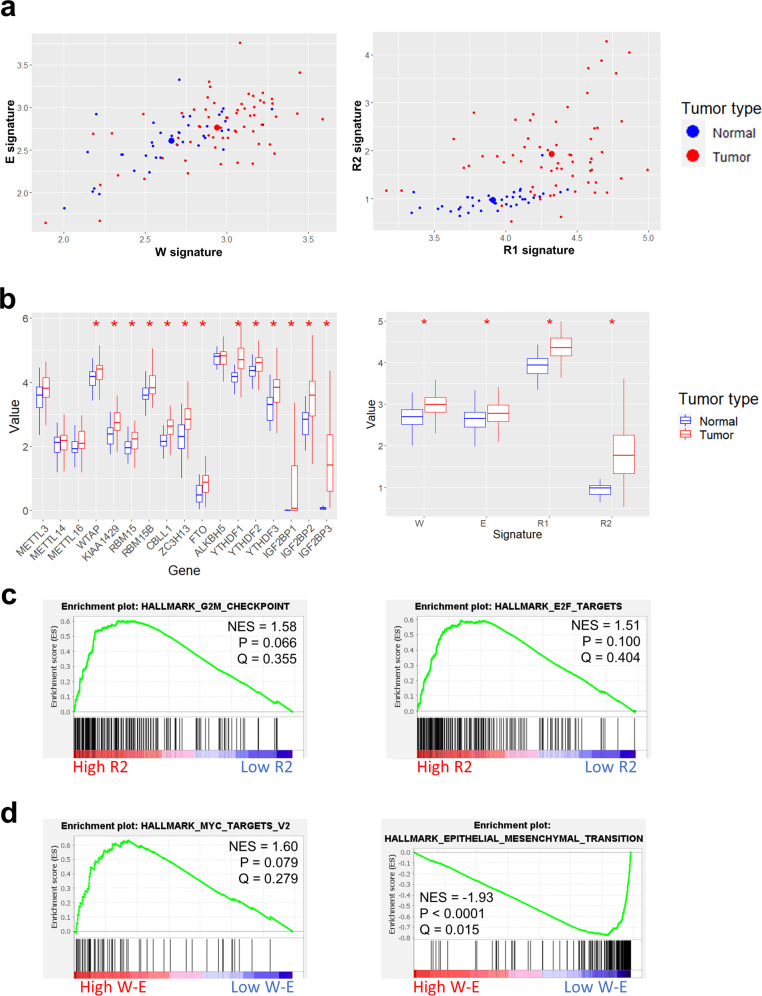
Analysis of m6A signatures in an in-house gastric cancer cohort. **a** Scatter plot of m6A signatures. The X and Y axes represent writer (W), eraser (E), and reader (R) signatures. Blue dots represent normal samples, and red dots represent tumor samples. **b** Box plot of the mRNA expression of each m6A-related gene and m6A signature. A red asterisk indicates significant differences between normal and tumor samples in the m6A gene expression or signature (*P* value < 0.05, estimated by *t* test). **c** Representative GSEA plots between the high and low R2 signature groups in the gastric cancer cohort. The G2M and E2F gene sets showed a tendency toward enrichment in the high R2 signature group. **d** Representative GSEA plots between the high and low W-E signature groups in the gastric cancer cohort. The Myc target gene set showed a tendency to be enriched in the high W-E signature group, and the EMT gene set was highly enriched in the low W-E signature group.

The expression of m6A-related genes in normal and tumor tissues in this cohort is consistent with several results in the TGCA database. First, we compared the W, E, and R signatures from cancer and normal tissues individually. The W signature showed a positive correlation with the E signature in both normal and cancer tissues (*R* = 0.645, *P* < 0.0001; Fig. 7a). In addition, the W and E signatures increased simultaneously in cancer tissues compared with normal tissues (Fig. 7b). The R2 signature was homogenously and significantly increased in cancer samples (Fig. 7a, b). The downregulation of the R1 signature in normal tissue was not as strong as that of R2, but a relative increase in the R1 signature in cancer tissue was observed as it was with the TCGA data (Fig. 1b, Fig. 7a, b). Investigation of the expression of individual m6A-related genes revealed that our gastric cancer samples had relatively increased expression of most m6A-related genes compared with normal gastric tissues, except for METTL3, METTL14, METTL16, and ALKBH5 (Fig. 7b), which are important genes for determining cellular m6A levels. These data were all compatible with the data from the TCGA stomach cohort (Fig. 1a) and suggest that an increase in the R2 signature, rather than alteration of m6A levels, is highly associated with gastric cancer development.

Next, we performed GSEA with our gastric cancer cohort and investigated whether there was a similar tendency with the GSEA results from the TCGA data. Dividing the tumor samples in the cohort into two groups based on high and low levels of the R2 signature or W-E signature, we analyzed the enriched gene sets between the groups. Analysis of the R2 signature showed the tendency of enrichment of gene sets such as G2/M checkpoint (normalized enrichment score (NES) = 1.58, *P* = 0.066) and E2F targets (NES = 1.51, *P* = 0.100) in the high R2 signature group (Fig. 7c). The R2 signature was significantly correlated with the expression of MKI67 (*R* = 0.333, *P* < 0.0001) (Supplementary Fig. 23a). In addition, the R2 signature showed a positive association with the EMT signature (*R* = 0.17, *P* = 0.082), which was estimated by the arithmetic means of EMT marker genes (CDH2, FN1, and VIM), but without statistical significance (Supplementary Fig. 23b). Therefore, the R2 signature in gastric cancer is correlated with cell proliferation and is probably associated with cancer development and progression.

On the other hand, GSEA of the high and low W-E signature groups showed that the MYC target gene set showed a tendency of enrichment in the high W-E signature group (NES = 1.60, *P* = 0.079), and the EMT gene set was significantly enriched in the low W-E signature group (NES = −1.93, *P* < 0.0001; Fig. 7d). These results are in agreement with the previous analyses of TCGA data (Fig. 4). An enriched EMT gene set in gastric cancer patients with a low W-E signature was associated with poor prognosis in these patients (Figs. 3 and 4).

### Cell type specificity underlying the W, E, and R signatures across cancer types

**Fig. 8:**
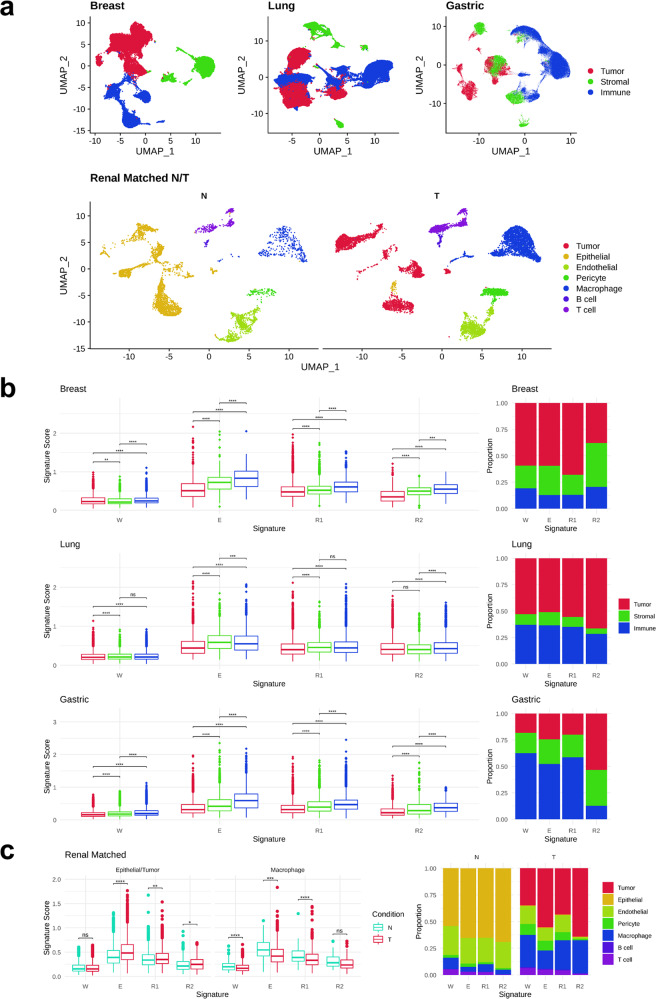
Single-cell RNA sequencing analysis of m6A-related signatures across cancer cell types. **a** UMAP representation of breast, lung, and gastric cancer single-cell datasets (top) and renal matched normal-tumor datasets (bottom). **b** Comparison of m6A writer (W), eraser (E), and reader signatures (R1 and R2) of breast, lung, and gastric cancer (left). Asterisks indicate adjusted P value outputs from the pairwise Wilcoxon rank sum test with Benjamini–Hochberg correction (ns: *P* ≥ 0.05; *P < 0.05; **P < 0.01; ***P < 0.001; ****P < 0.0001). The proportion of each cell type in the overall m6A signature expression (right). **c** Comparison of m6A writer (W), eraser (E), and reader signatures (R) of matched tumor (T) and normal (N) samples from the renal cancer dataset (left). Asterisks indicate *P* value outputs from the pairwise binomial test (ns: *P* ≥ 0.05; *P < 0.05; **p < 0.01; ***p < 0.001; ****p < 0.0001). The proportion of cells in the overall m6A signature expression by cell type (right).

To evaluate the cell types associated with m6A modification in cancer tissues, we compared the cell-type-specific expression of m6A-related genes using the scRNA-seq data of tumors obtained from patients. First, we analyzed the scRNA-seq datasets of breast cancer, non-small-cell lung cancer, and gastric cancer, which are publicly available in the GEO database, and identified clusters for tumor, stromal and immune cells (Fig. 8a). In general, m6A-related genes showed higher expression in nontumor cells than in tumor cells in all three cancer types (Fig. 8b). However, there was a striking difference in the cellular proportion of m6A-related gene expression (Fig. 8b), which was calculated by the proportion of the sum of the m6A signatures in each cell type to the sum of m6A signatures from total cells. Over half of all m6A signatures were derived from tumor cells in lung cancer, and the W, E, and R1 signatures were derived from tumor cells in breast cancer (Fig. 8b). For gastric cancer, the majority of the W, E, and R1 signatures were from immune cells. However, for the R2 signature, tumor cells were the main cells with the expression of signature genes (Fig. 8b). Despite the gene expression at the individual cell level, m6A-related genes seem to have pervasive regulation in tumor cells rather than nontumor cells.

Next, we identified m6A-related genes with cell-type specificity on tumor cells. In breast cancer, eight genes (METTL3, KIAA1429, ZC3H13, FTO, YTHDF1, YTHDF2, IGF2BP2, and IGF2BP3) were significantly enriched in tumor cells compared to immune or stromal cells (Supplementary Table 1). YTHDF1 and ZC3H13 were also enriched in nontumor cells, but the percentage of cells with gene expression and the average fold-changes were higher in tumor cells than in nontumor cells. Lung cancer and gastric cancer each had a single gene (YTHDF2 and IGF2BP2, respectively) enriched in tumor cells (Supplementary Table 1).

We further delineated the pattern of m6A-related gene expression using tumor tissues and matched normal tissues. We obtained scRNA-seq data from the latest study of renal clear cell cancer, which contained paired tumor and normal samples from the same individual, and identified cell-type clusters for tumor, stromal and immune cells (Fig. 8a). To evaluate the expression of the m6A signature genes, we analyzed tumor cells in tumor samples against epithelial cells in matched normal samples. The E, R1, and R2 signatures were significantly higher in tumor cells than in normal epithelial cells in renal cancer (Fig. 8c), which is consistent with the bulk tissue RNA sequencing of KIRC (Fig. 1b). When comparing macrophages from tumor samples to macrophages from normal samples, normal macrophages showed higher m6A signatures than tumor macrophages (Fig. 8c). In all m6A-related signatures, cancer cells comprised the largest proportion of the total m6A signature (Fig. 8c).

We further investigated whether the R2 signature was correlated with proliferation markers in tumor cells (Supplementary Fig. 24). We found that the expression of MKI67 (encodes Ki-67) is significantly correlated with the R2 signature in tumor cells (*P* = 2.4E-4, breast cancer; *P* < 2.2E-16, lung and gastric cancer), implicating the R2 signature in tumor progression. Consistent with this, the EMT genes showed a significant positive correlation with the R2 signatures in tumor cells (*P* = 7.1E-4, breast cancer; *P* = 1.6E-11, lung cancer).

## Discussion

The dysregulation of cellular RNA m6A modification is one of the characteristics of carcinogenesis because alterations in m6A levels and m6A-related genes result in the remodeling of gene expression at the posttranscriptional level^8^. However, whether the alterations in m6A levels and m6A-related genes are oncogenic or tumor-suppressive varies according to the cancer type. Therefore, the common underlying mechanism of RNA m6A methylation responsible for carcinogenesis across cancer types is not fully understood. In this study, we investigated the common features of RNA m6A alterations during carcinogenesis by analyzing the transcriptomes of 11 solid tumors.

RNA m6A modification-mediated gene expression regulation is determined by two factors: (1) the m6A levels of target genes, which are balanced by the addition of m6A (mediated by the RNA m6A methyltransferase complex, writers) and the removal of m6A (mediated by RNA m6A demethylases, erasers); and (2) m6A-binding proteins (readers), which are responsible for m6A-mediated mRNA metabolism, including splicing, transport, translation, and degradation^2^. To understand the general changes in these two factors during carcinogenesis, we estimated the W, E, and R signatures in normal and cancer tissues. We speculated that cellular m6A levels are estimated by the ratio of the W and E signatures. Although we could not validate this assumption due to a lack of m6A level data in the TCGA database, there are some points that support the simultaneous consideration of writer and eraser genes. First, the expression levels of not only major components of methyltransferase (WTAP, METTL3, and METTL14) and demethylase (FTO and ALKBH5) but also appendix genes (KIAA1429, RBM15, RBM15B, CBLL1, and ZC3H13) have been shown to have a significant effect on m6A levels^51–53^. In addition, individual m6A-related genes regulate methylation status at different sites. For example, considering only FTO in the W-E signature cannot represent m6A states in specific targets of ALKBH5^54^. However, the correlation between cellular m6A levels and the W-E signature needs to be validated experimentally.

Because RNA m6A-binding proteins have diverse functions in RNA metabolism, we adopted two R signatures: the R1 signature, consisting of YTHDF1–3, and the R2 signature, consisting of IGF2BP1–3. Previous studies suggested that YTHDF1 enhances mRNA translation^55^, while YTHDF2 promotes mRNA degradation^56^. YTHDF3 was reported to be involved in both mRNA translation and degradation through YTHDF1 and YTHDF2, respectively^57^. However, recent studies suggest that the function of YTHDF1–3 to mediate mRNA degradation is redundant^58,59^ based on sequence and structural similarity. Considering the redundant functions of YTHDF1–3, we evaluated the role of YTHDF proteins across cancer types by calculating the R1 signature based on the expression of YTHDF1–3. We also estimated the R2 signature based on the expression of IGF2BP1-3 because IGF2BPs show redundant functions by enhancing mRNA stability^60^. Some studies claim functional dissimilarity of IGF2BPs owing to their target specificities^61^. However, IGF2BPs have a common KH domain and a similar distribution in cells^60^. In addition, each IGF2BP binds to more than 3,000 mRNA transcripts, and more than 2,000 mRNAs, including MYC, FSCN1, TK1 and MARCKSL1, are targeted by all three IGF2BPs^60^. Thus, the resemblance of target genes, structure, position, and function of IGF2BPs support our integrated analysis based on the R2 signature.

Previous studies showed that inhibition of IGF2BPs, which form the R2 signature, decreased cancer aggressiveness. Knockdown or knockout of IGF2BPs inhibited cell proliferation, colony formation, migration and invasion in cervical carcinoma HeLa cells and hepatocellular carcinoma HepG2 cells^60^. Knockout of IGF2BPs also inhibited colony formation, migration, and in vivo tumor formation in renal cell carcinoma 786-0 and Caki-1 cells^62^. In addition, a small molecule inhibitor of IGF2BP1 suppressed cell migration and colony formation in soft agar in non-small-cell lung carcinoma H1299 cells^63^. We also found that knockdown of IGF2BPs with siRNA decreased cell proliferation in breast cancer MDA-MB-231 cells and gastric cancer MKN1 cells (Supplementary Fig. 25). These data suggest that increased levels of IGF2BPs play crucial roles in cancer progression and aggressiveness.

When we estimated the global RNA m6A levels based on the W-E signature, we found that the W and E signatures were positively correlated in all 11 tumor types (Fig. 1c and Supplementary Fig. 1), suggesting that writers and erasers regulate each other’s effect on the cellular homeostasis of RNA m6A levels^64^. In addition, the prognostic effect of RNA m6A levels estimated by the W-E signature was diverse according to the cancer type (Fig. 3). Therefore, we could not find a common increase or survival effect of RNA m6A levels across cancer types, which suggests that simple increases or decreases in m6A levels are not associated with cancer development and prognosis.

At the molecular pathway level, a high W-E signature promoted cell proliferation across cancer types via high expression of the G2/M checkpoint and E2F/MYC target genes (Fig. 4). Previous studies reported that METTL3 and 14 act as oncogenes in several types of cancer by regulating DRG1 and MYC, respectively^65,66^. However, in tumor types showing better prognosis in patients with a high W-E signature (Cluster 2; BLCA, BRCA, STAD, LUSC, and COAD), high W-E signature samples had lower expression of EMT genes and a relatively higher proportion of early-stage cancer (Fig. 4 and Supplementary Fig. 7). A previous study showed that METTL3 promotes EMT via m6A methylation in Snail mRNA when TGFβ was applied to HeLa and HepG2 cells^67^. In contrast, METTL14 inhibits GC cell invasiveness by deactivating Wnt and PI3K-Akt signaling^68^. Therefore, increased m6A levels are associated differently with EMT according to the tumor type. In addition, m6A-mediated differential regulation of the TME is also associated with patient prognosis. Patients in Cluster 2 with a low W-E signature had high levels of M2 macrophages (Supplementary Fig. 12). However, patients with a high W-E signature in Cluster 1 (KIRC, LIHC, and UCEC), who showed worse prognoses than patients without a high W-E signature, had high levels of M0 macrophages and eosinophils (Supplementary Fig. 12). Lower m6A levels in myeloid cells enhance cancer progression by blocking the YTHDF1-mediated translation of SPRED2, and knockout of METTL3 induced a higher count of M2 macrophages in the TME^69^. Therefore, m6A-mediated regulation of tumor-infiltrating myeloid cells is associated with patient prognosis in a tumor type-specific manner. However, the underlying molecular mechanism of these tumor type-specific effects of m6A levels on EMT and immune cell infiltration need to be further investigated.

Although the alterations in m6A levels had a diverse effect on patient prognosis across cancer types, augmentation of the R signature in tumor tissues was significant in most cancer types (Fig. 1a, b). In particular, the R2 signature and the expression of IGF2BPs were ubiquitously related to poor prognosis (Fig. 5). Several studies have shown that each IGF2BP is related to cell proliferation, MYC signaling, and the G2/M checkpoint^70,71^ and has an oncogenic effect in multiple cancers^34^. Consequently, the higher expressor of the R2 signature had a higher enrichment of not only the G2M, MYC, and E2F gene sets but also the EMT and apical junction gene sets in GSEA (Fig. 6). These data suggest that alterations in RNA m6A-binding proteins are more critical in carcinogenesis and patient prognosis than alterations in the m6A level itself. Therefore, targeting m6A reader proteins is a promising strategy for cancer treatment across cancer types.

## Supplementary information

Supplementary Materials
