# Supplementary material for: Augmentation of the RNA m6A reader signature is associated with poor survival by enhancing cell proliferation and EMT across cancer types
Source: Exp Mol Med. 2022 Jul 6;54(7):906–21. doi: 10.1038/s12276-022-00795-z (PMC9355997; doi:10.1038/s12276-022-00795-z)
Supplement: Supplementary file 1 — Supplementary Materials [file 12276_2022_795_MOESM1_ESM.pdf]

Supplementary Materials

Augmentation of RNA m6A reader signature is associated with poor survival by enhancing cell proliferation and EMT across cancer types

Jaeik Oh, Chanwoong Hwa, Dongjun Jang, Seungjae Shin, Soo-Jin Lee, Jiwon Kim, Sang Eun Lee, Hae Rim Jung, Yumi Oh, Giyong Jang, Obin Kwon, Joon-Yong An, Sung-Yup Cho

Supplementary Figures 1 – 25

Supplementary Table 1

Supplementary Figures

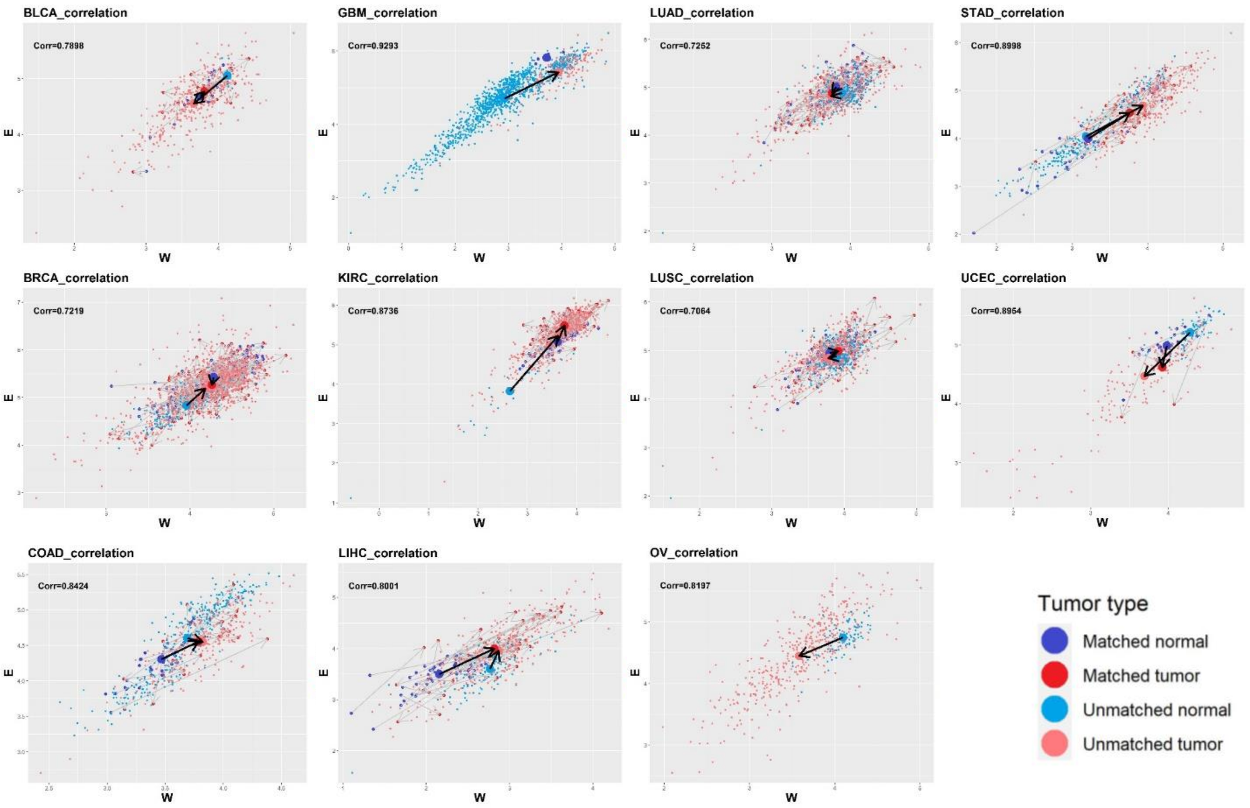

Supplementary Figure 1. Scatter plot of m6A writer and eraser signatures across cancer types.

Scatter plot of m6A W and E signatures in log scales. Blue dots are normal tissue sample that is adjacent to the tumor samples, which are marked as red dots. Sky blue and pink dots are normal tissue and tumor samples, respectively, that are not matched. Mean W and E signature are marked with larger dots. Pearson correlation coefficients are written on the plot. Arrows indicate each pair of samples and starts from normal tissue to tumor sample.

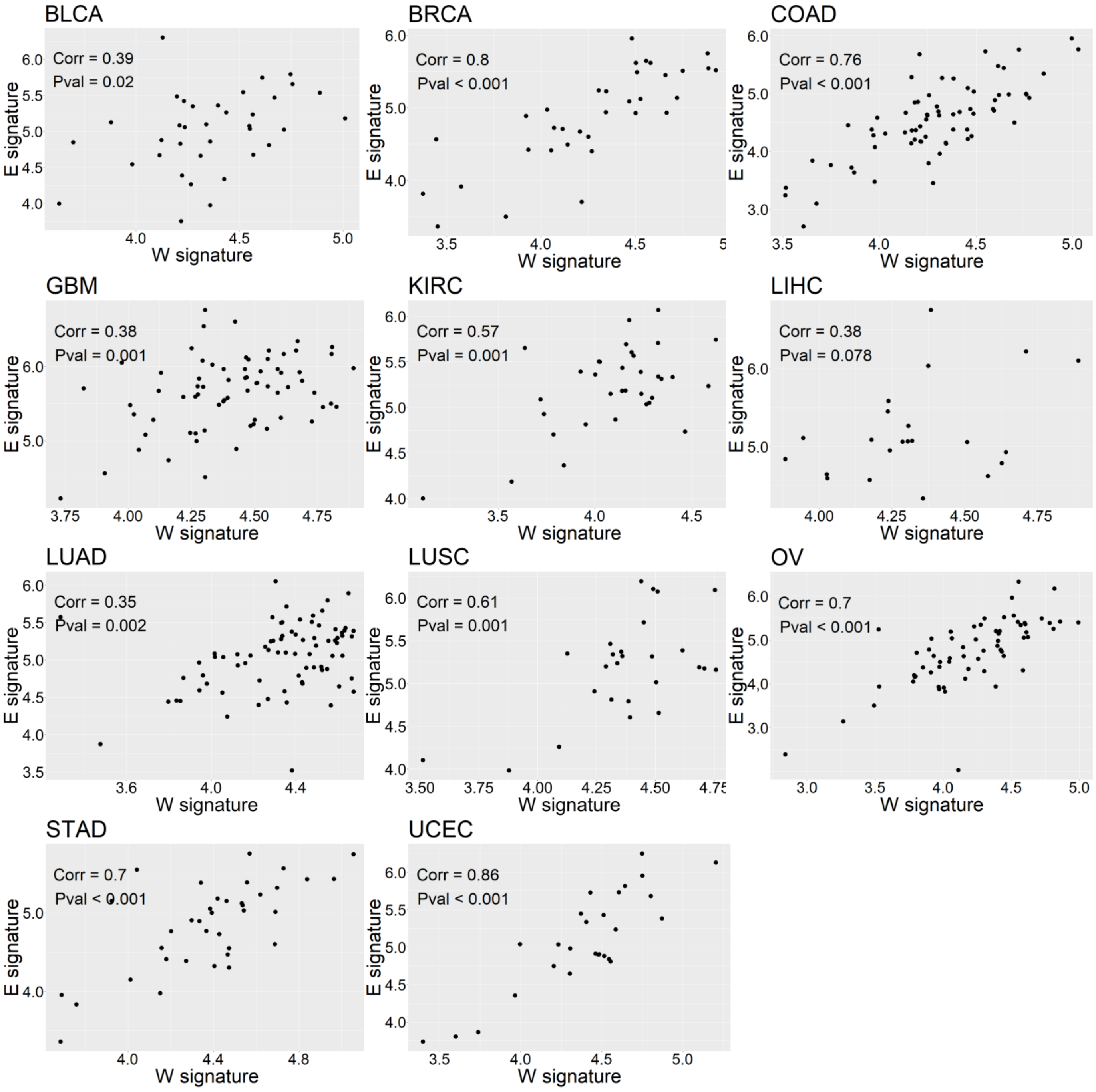

Supplementary Figure 2. Scatter plot of m6A writer and eraser signatures in cancer cell lines.

Scatter plot of m6A W and E signatures in log scales for 11 cancer types from CCLE cell line data. Pearson correlation coefficients (Corr) are written on the plot.

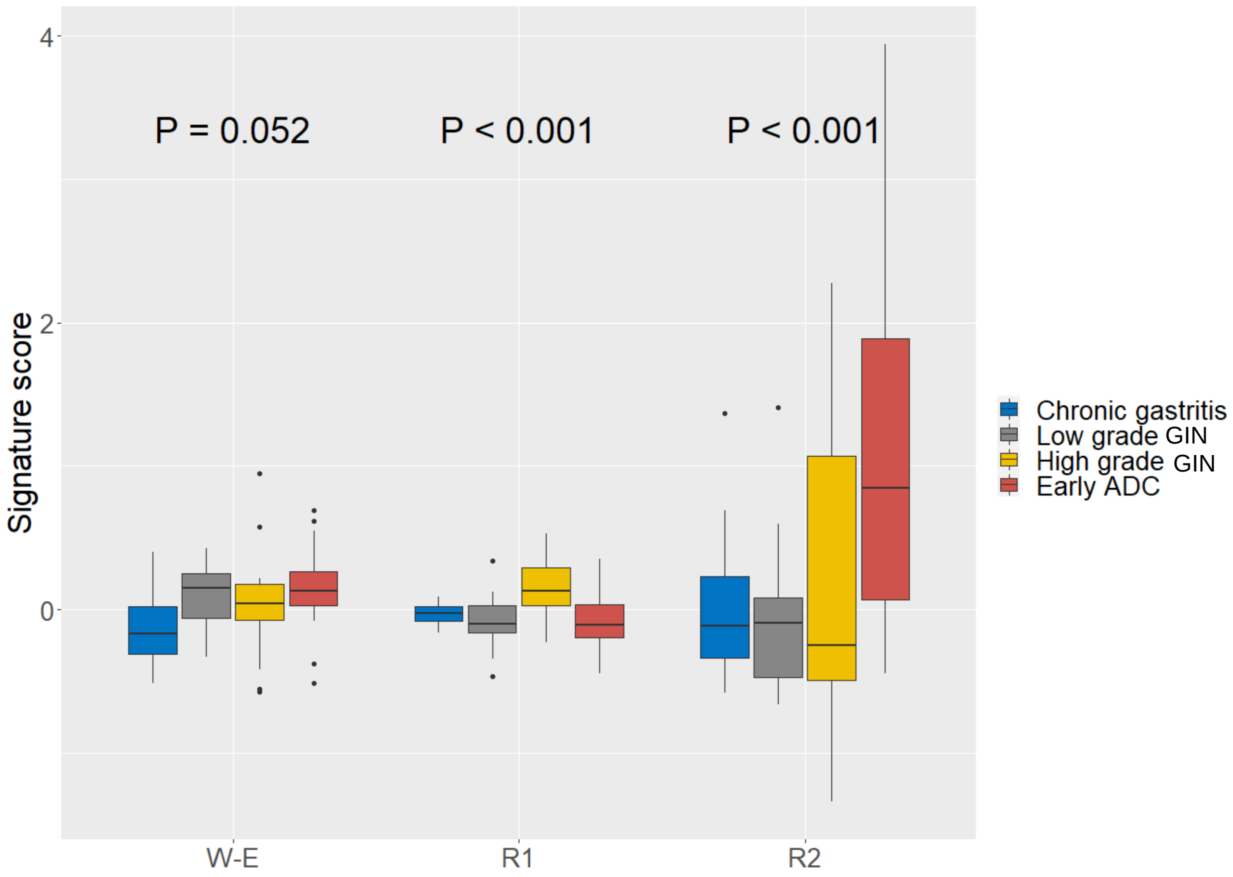

Supplementary Figure 3. Alterations of m6A W, E, and R signatures during gastric cancer carcinogenesis.

The m6A-related signatures were estimated using data set from Gene Expression Omnibus database (GSE55696). The W, E, and R signatures were demonstrated as box plot for chronic gastritis, low and high grade gastric intraepithelial neoplasia (GIN). P-values between tissue types are written on the plot estimated by one-way analysis of variance (ANOVA).

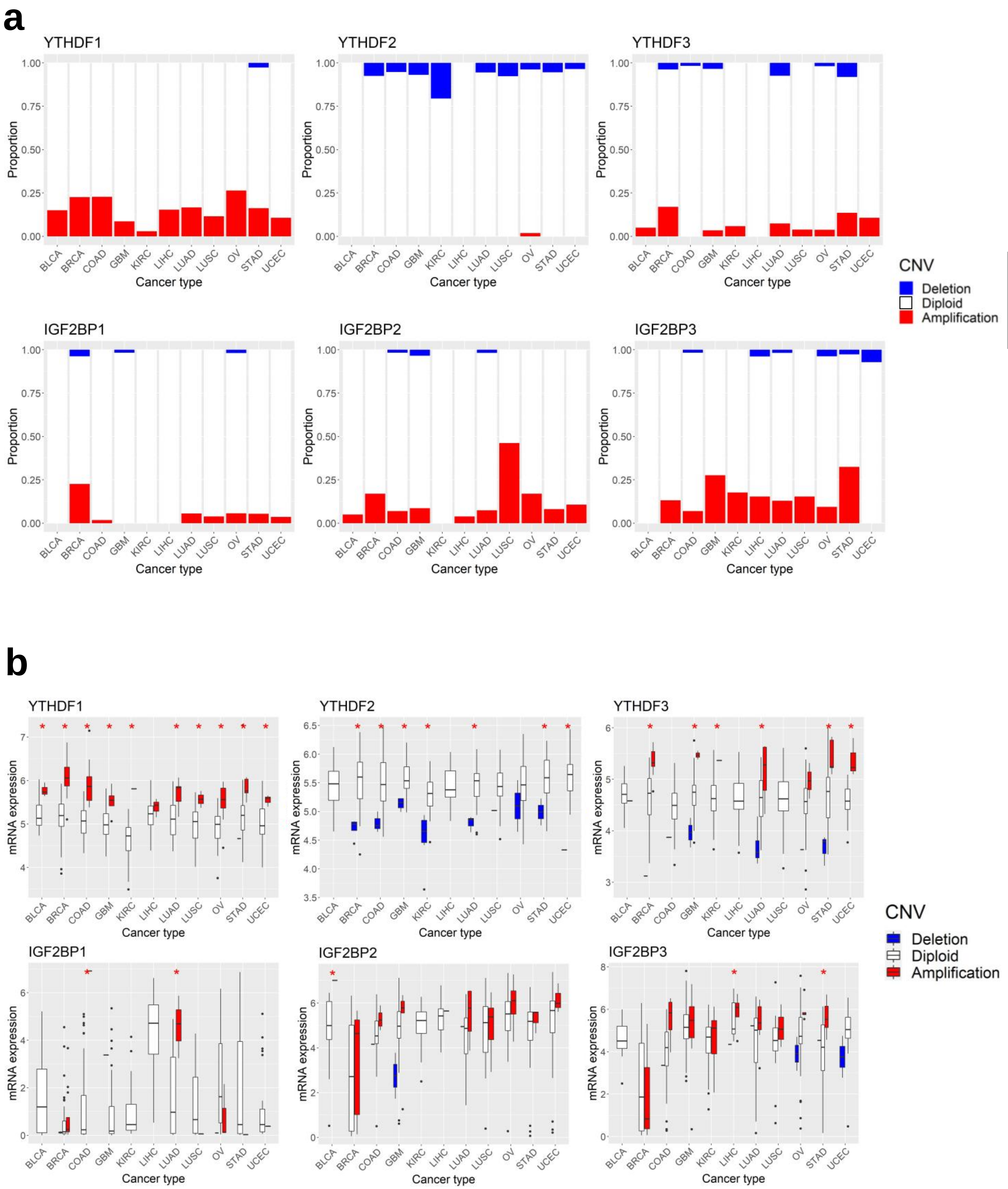

a
YTHDF1
YTHDF2
YTHDF3
IGF2BP1
IGF2BP2
IGF2BP3
Proportion
Cancer type
BLCA BRCA COAD GBM KIRC LIHC LUAD LUSC OV STAD UCEC
CNV
Deletion
Diploid
Amplification
b
YTHDF1
YTHDF2
YTHDF3
IGF2BP1
IGF2BP2
IGF2BP3
mRNA expression
Cancer type
CNV
Deletion
Diploid
Amplification

Supplementary Figure 4. Copy number alterations of m6A reader genes in cancer cell lines.

a The proportion of cell lines with copy number alteration of m6A reader genes across cancer types. From blue to red, each color stands for ‘deletion’, ‘diploid’, and ‘amplification’. Data were extracted from putative GISTIC2 score in DepMap cellular model expression data (https://depmap.org/portal/download/). Types of tumors in x axis are listed in alphabetical. **b** Correlation between copy number alteration and mRNA expressions of m6A reader genes. Each mRNA expression that correspond to its copy number is demonstrated from deletion to amplification. Each type of cancer is listed in x axis alphabetically and mRNA expression is demonstrated in log scale. The asterisk marks data for which the eta coefficient between mRNA expression and CNV is > 0.3.

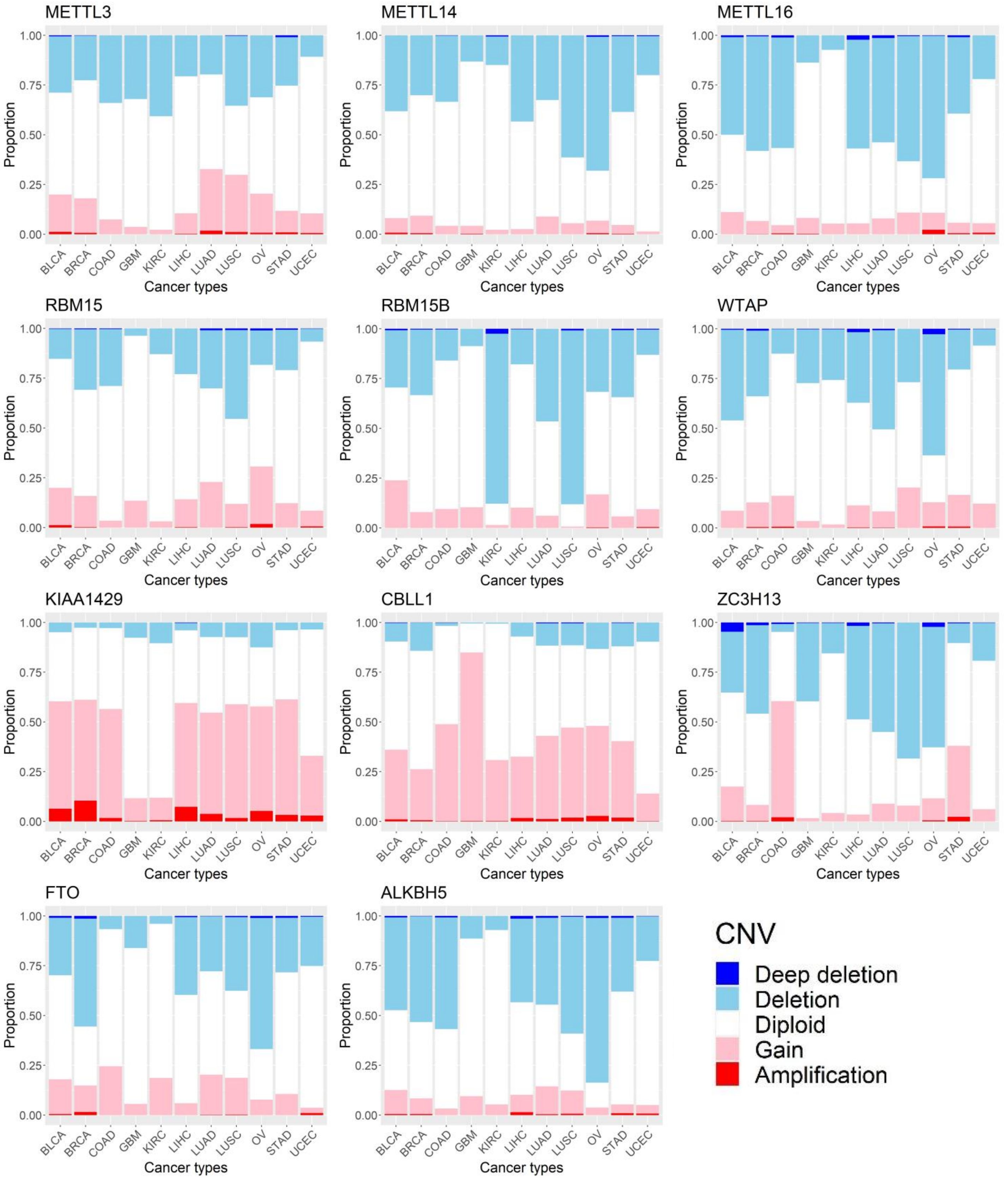
METTL3
METTL14
METTL16
RBM15
RBM15B
WTAP
KIAA1429
CBLL1
ZC3H13
FTO
ALKBH5
Proportion
0.00
0.25
0.50
0.75
1.00
BLCA
BRCA
COAD
GBM
KIRC
LIHC
LUAD
LUSC
OV
STAD
UCEC
Cancer types
CNV
Deep deletion
Deletion
Diploid
Gain
Amplification

Supplementary Figure 5. The proportion of patients with copy number alteration of m6A writer and eraser genes across cancer types.

From blue to red, each color stands for ‘deep deletion’, ‘deletion’, ‘diploid’, ‘gain’, and ‘amplification’. Data were extracted from putative GISTIC copy number variation (CNV) in cBioPortal (https://www.cbioportal.org/). Deletions are more frequent than gain or amplifications in all eraser genes and most of writer genes except KIAA1429 and CBLL1. Types of tumors in x axis are listed in alphabetical.

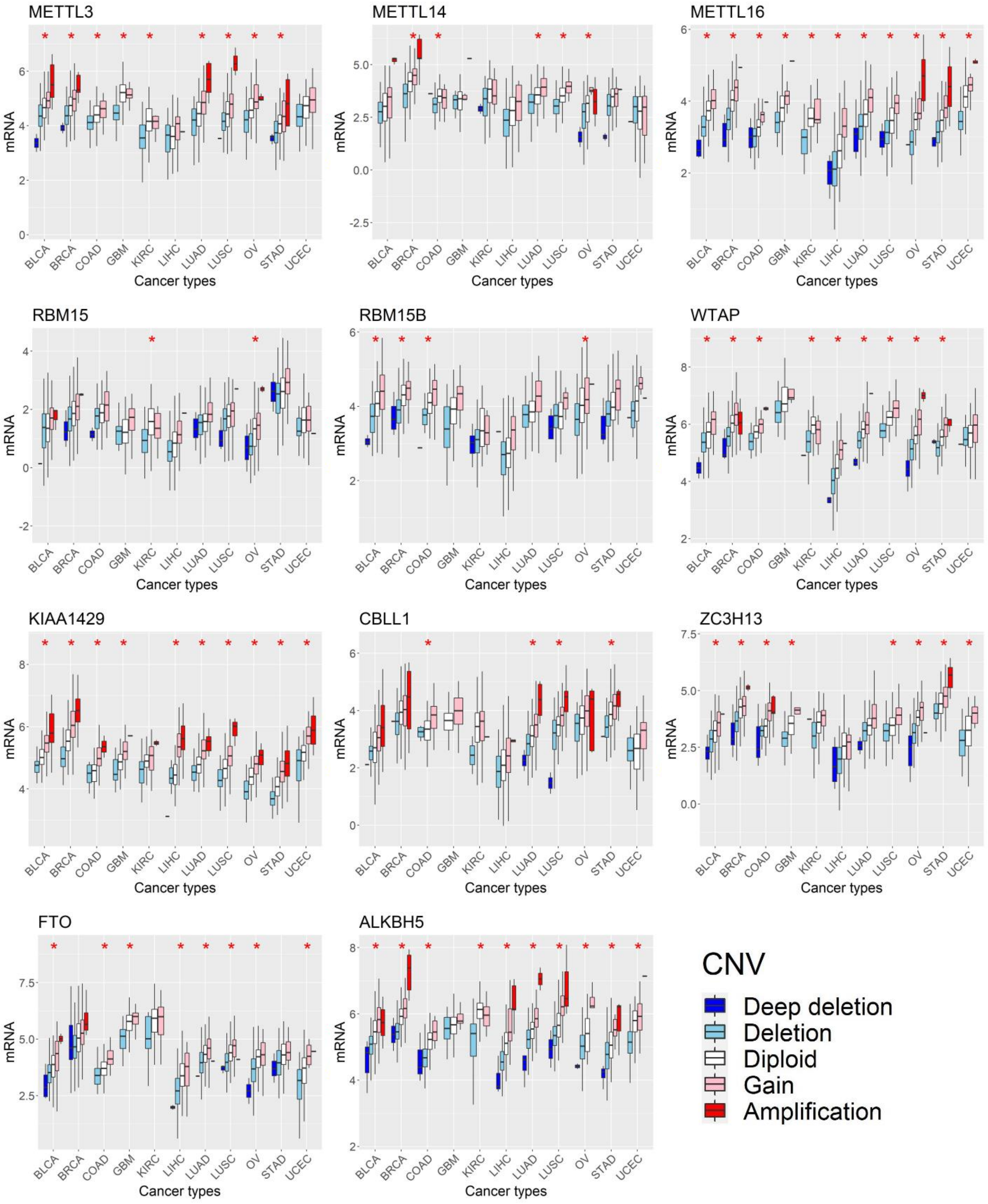
METTL3
METTL14
METTL16
RBM15
RBM15B
WTAP
KIAA1429
CBLL1
ZC3H13
FTO
ALKBH5
mRNA
Cancer types
BLCA
BRCA
COAD
GBM
KIRC
LIHC
LUAD
LUSC
OV
STAD
UCEC
CNV
Deep deletion
Deletion
Diploid
Gain
Amplification

Supplementary Figure 6. Correlation between copy number alteration and mRNA expressions of m6A writer and eraser genes.

Each mRNA expression that correspond to its copy number is demonstrated from deep deletion to amplification. Each type of cancer is listed in x axis alphabetically and mRNA expression is demonstrated in log scale. The asterisk marks data for which the eta coefficient between mRNA expression and CNV is > 0.3.

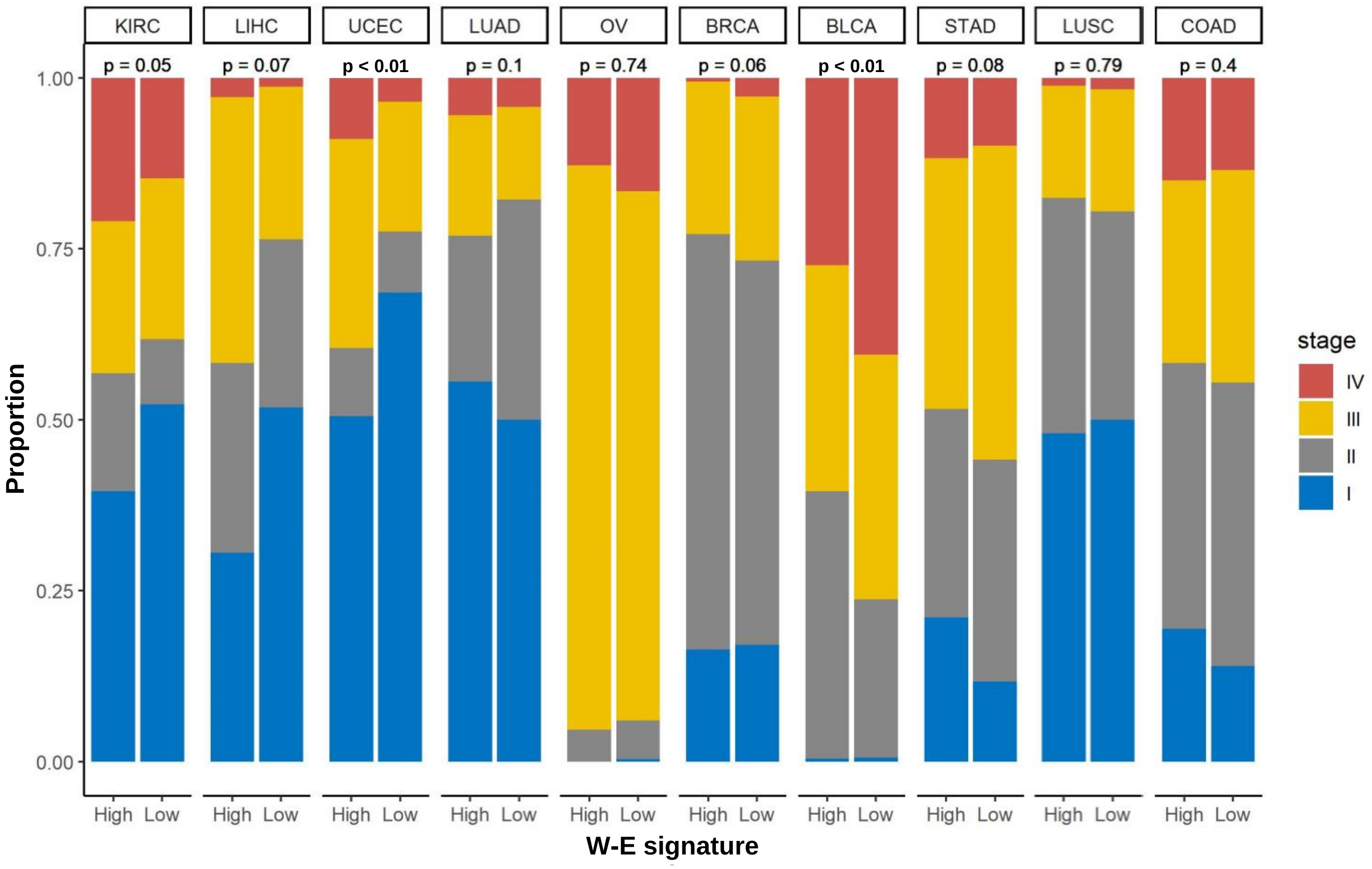

Supplementary Figure 7. Distribution of disease stage in high and low m6A W-E signatures across cancer types.

From the bottom of plot, blue, gray, yellow and red stand for stage I, II, III and IV. Substages are integrated. High W-E signature is on left side and low W-E signature is on right side of plot. P-values from chi square test are demonstrated at the top of plot.

a

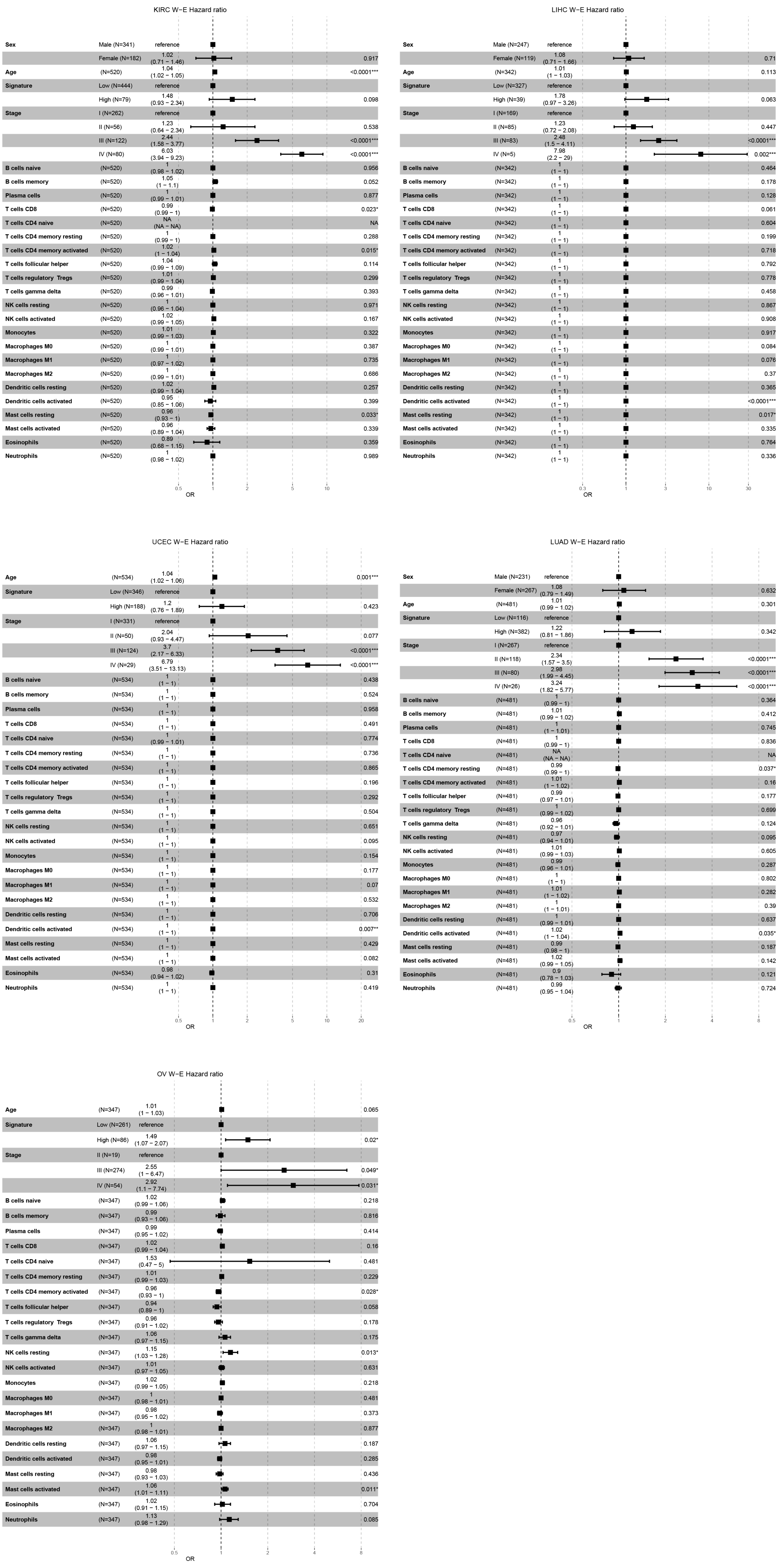
KIRC W−E Hazard ratio
LIHC W−E Hazard ratio
UCEC W−E Hazard ratio
LUAD W−E Hazard ratio
OV W−E Hazard ratio
OR

b

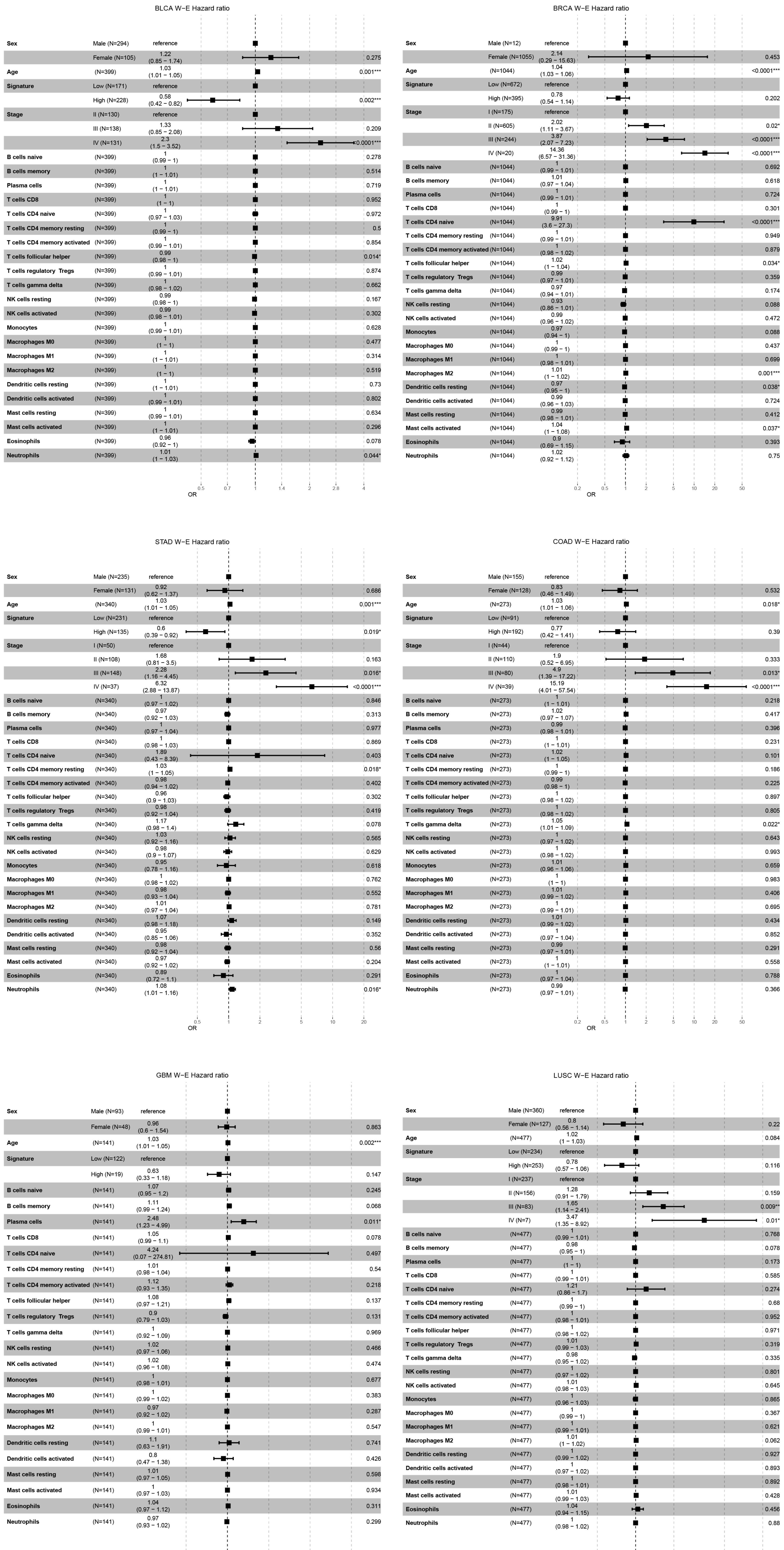
BLCA W−E Hazard ratio
BRCA W−E Hazard ratio
STAD W−E Hazard ratio
COAD W−E Hazard ratio
GBM W−E Hazard ratio
LUSC W−E Hazard ratio
OR

Supplementary Figure 8. Multivariate cox regression model between high and low m6A W-E signatures across cancer types.

a, b For multivariate cox regression model, we used sex, age, stage, the amounts of immune cells, and m6A W-E signature. Some variates that cannot include in analysis such as stage in GBM or gender in UCEC were excluded. Because there are only 19 and 1 patients with stage I bladder and ovarian cancer, respecively, stage I are excluded from the analysis in both types of tumor. Forrest plots of variates are demonstrated. Tumors that have worse survival in high W-E signature were demonstrated in (a), and tumors that have favorable survivals in high W-E signature were demonstrated in (b).

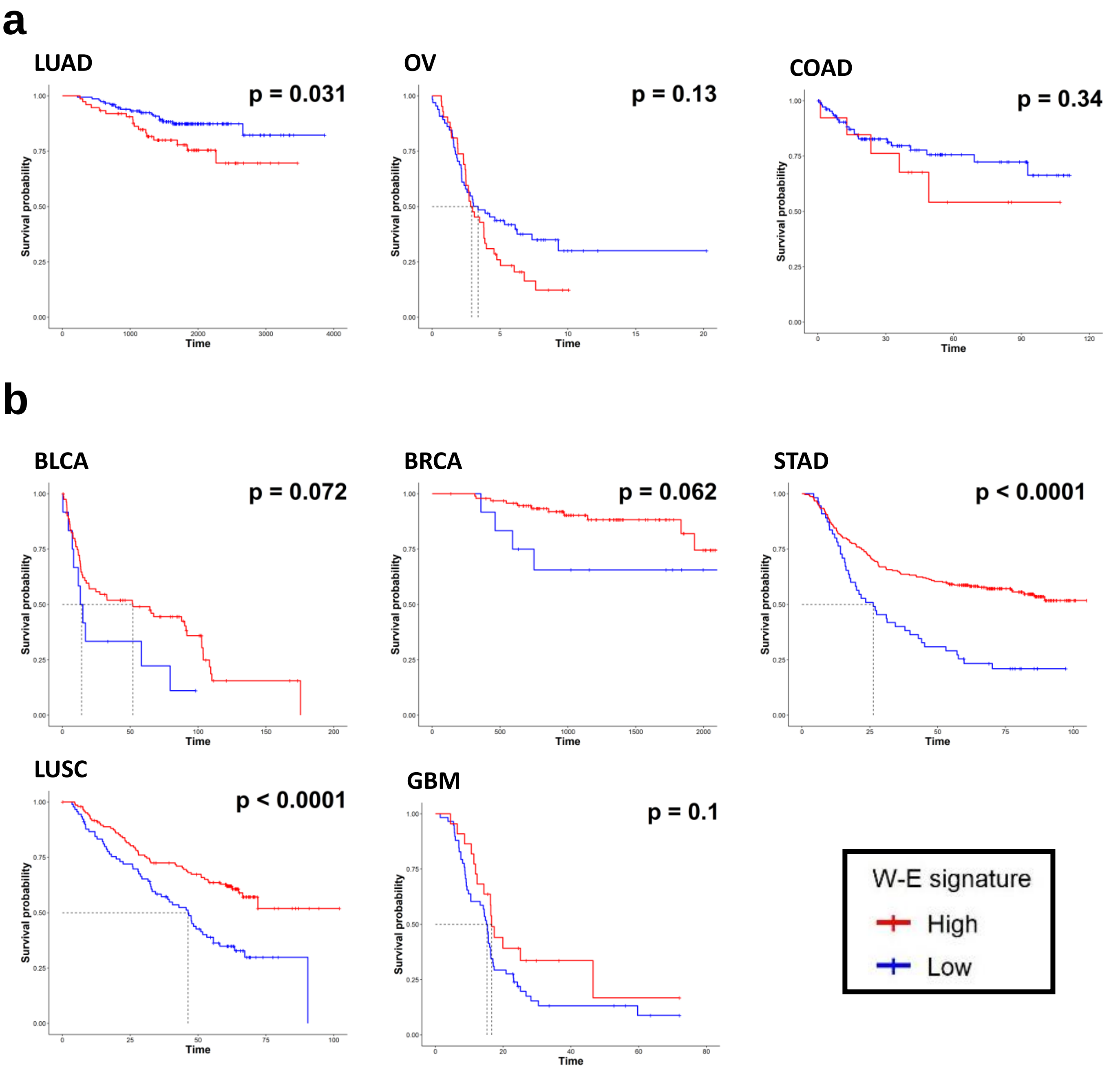

Supplementary Figure 9. Survival analysis according to m6A W-E signature across cancer types in validation cohort.

a, b Kaplan-Meier plots for overall survival of patients with high and low W-E signatures. Gene expression data were analyzed using Gene Expression Omnibus (GEO) database: GSE31684 (BLCA), GSE16446 (BRCA), GSE38832 (COAD), GSE7696 (GBM), GSE31210 (LUAD), GSE157010 (LUSC), GSE26193 (OV) and GSE62254 (STAD). Red and blue lines stand for samples with high and low W-E signature, respectively. Each median survival and P-value, determined by log rank test, is shown. Tumors that have worse survival in high W-E signatures are demonstrated in (a), and tumors that have favorable survivals in high W-E signature are demonstrated in (b).

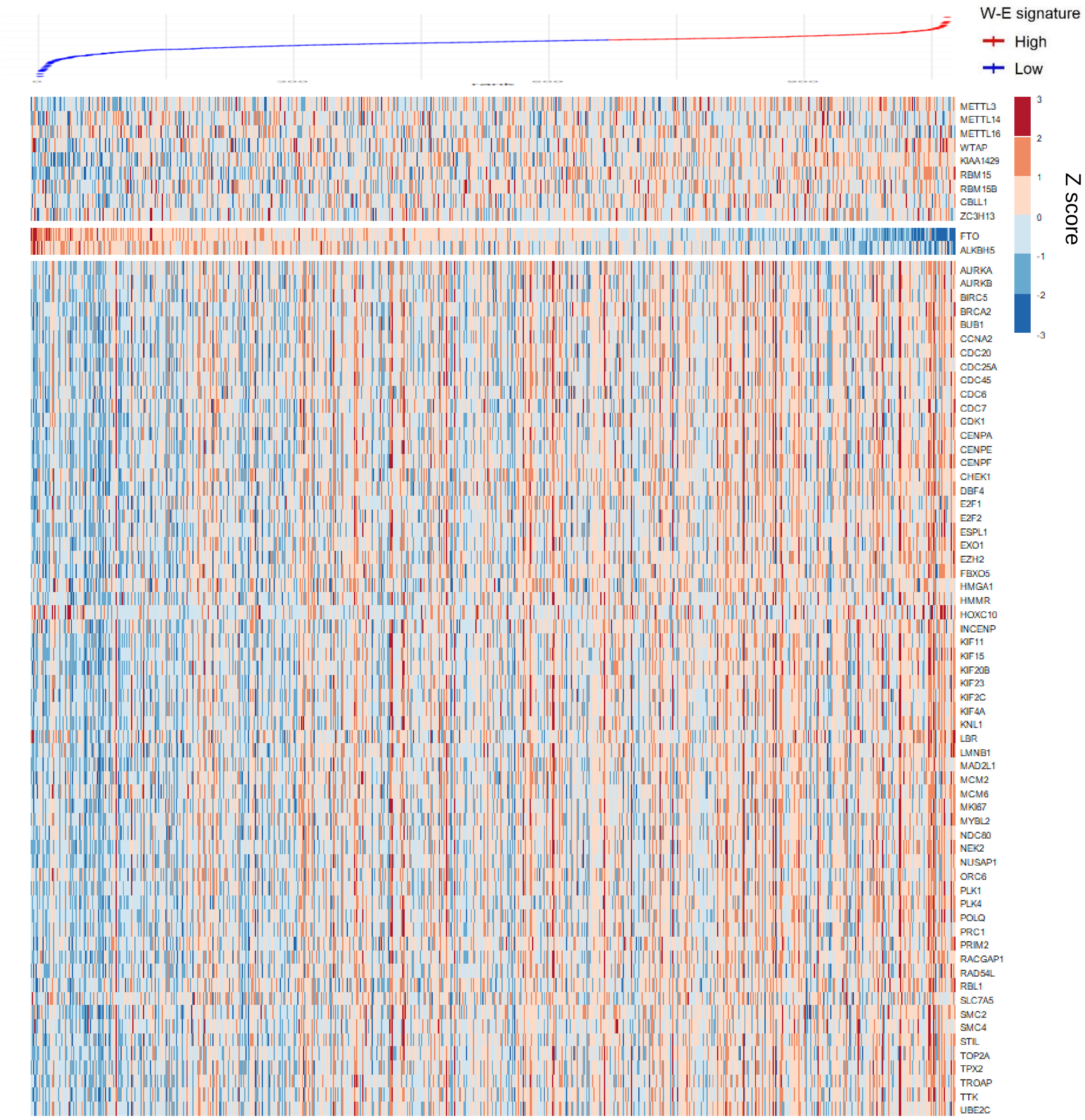

Supplementary Figure 10. Heatmap for mRNA expression of genes included in G2M checkpoint gene sets from breast cancer samples.

Each column stands for an individual cancer sample, and each row denotes a gene. BRCA samples are sorted from left to right by W-E signature in ascending order. At the top of heatmap, W-E signature is presented and discriminated into high and low groups by the color (red: high, blue: low). Among 200 genes in G2M checkpoint gene set, 62 genes are commonly enriched in LIHC, BRCA, LUAD, and OV. Each cell in heatmap indicates stratified Z score (standard deviation, from -3 to 3) of 9 writer genes, 2 eraser genes and 62 genes.

a

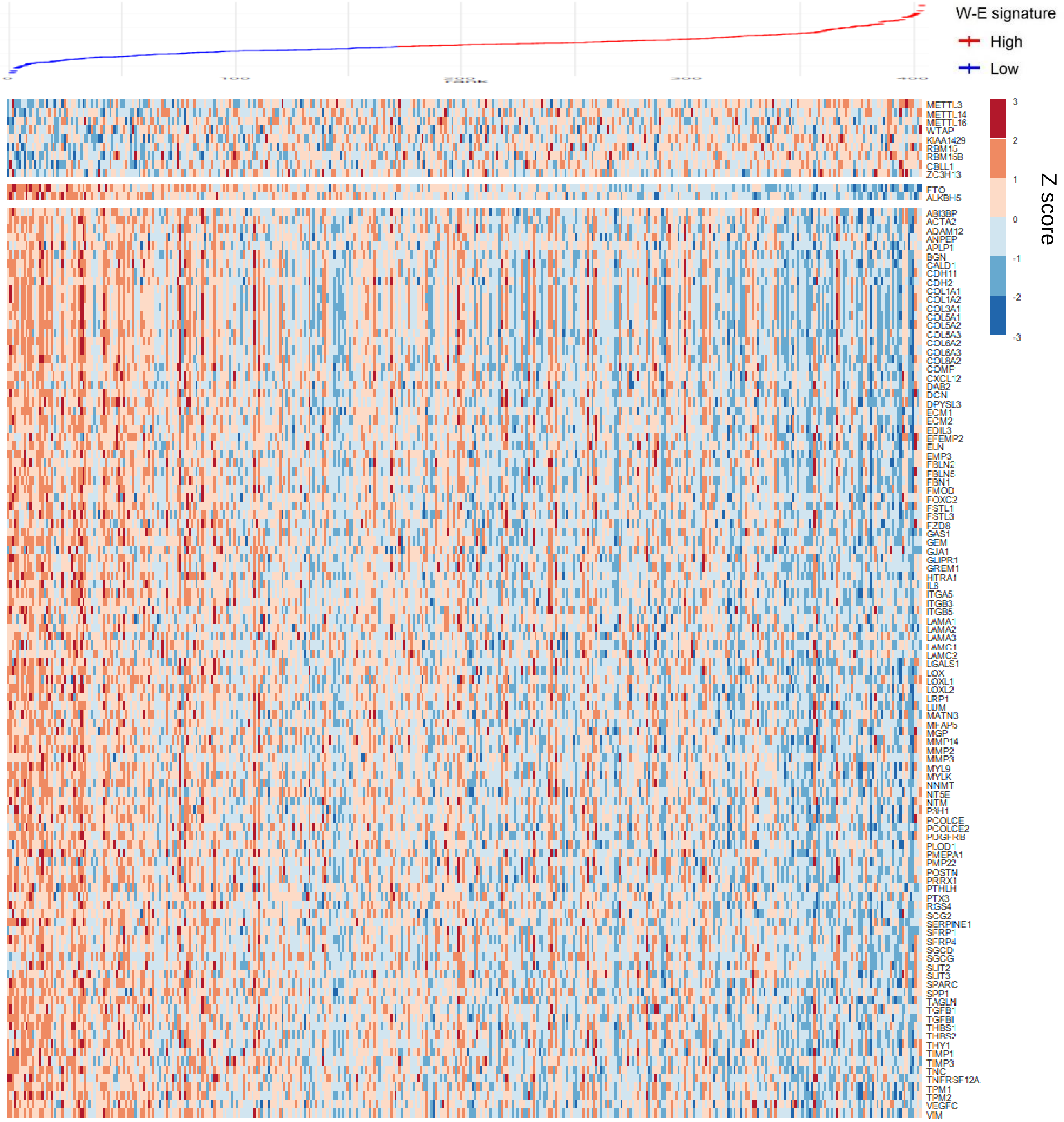
W-E signature
High
Low
METTL3
METTL14
METTL16
WTAP
KIAA1429
RBM15
RBM15B
CBLL1
ZC3H13
FTO
ALKBH5
ABI3BP
ACTA2
ADAM12
ANPEP
APLP1
BGN
CALD1
CDH11
CDH2
COL1A1
COL1A2
COL3A1
COL5A1
COL5A2
COL5A3
COL6A2
COL6A3
COL8A2
COMP
CXCL12
DAB2
DCN
DPYSL3
ECM1
ECM2
EDIL3
EFEMP2
ELN
EMP3
FBLN2
FBLN5
FBN1
FMOD
FOXC2
FSTL1
FSTL3
FZD8
GAS1
GEM
GJA1
GLIPR1
GREM1
HTRA1
IL6
ITGA5
ITGB3
ITGB5
LAMA1
LAMA2
LAMA3
LAMC1
LAMC2
LGALS1
LOX
LOXL1
LOXL2
LRP1
LUM
MATN3
MFAP5
MGP
MMP14
MMP2
MMP3
MYL9
MYLK
NNMT
NT5E
NTM
P3H1
PCOLCE
PCOLCE2
PDGFRB
PLOD1
PMEPA1
PMP22
POSTN
PRRX1
PTHLH
PTX3
RGS4
SCG2
SERPINE1
SFRP1
SFRP4
SGCD
SGCG
SLIT2
SLIT3
SPARC
SPP1
TAGLN
TGFB1
TGFBI
THBS1
THBS2
THY1
TIMP1
TIMP3
TNC
TNFRSF12A
TPM1
TPM2
VEGFC
VIM
3
2
1
0
-1
-2
-3
Z score

b

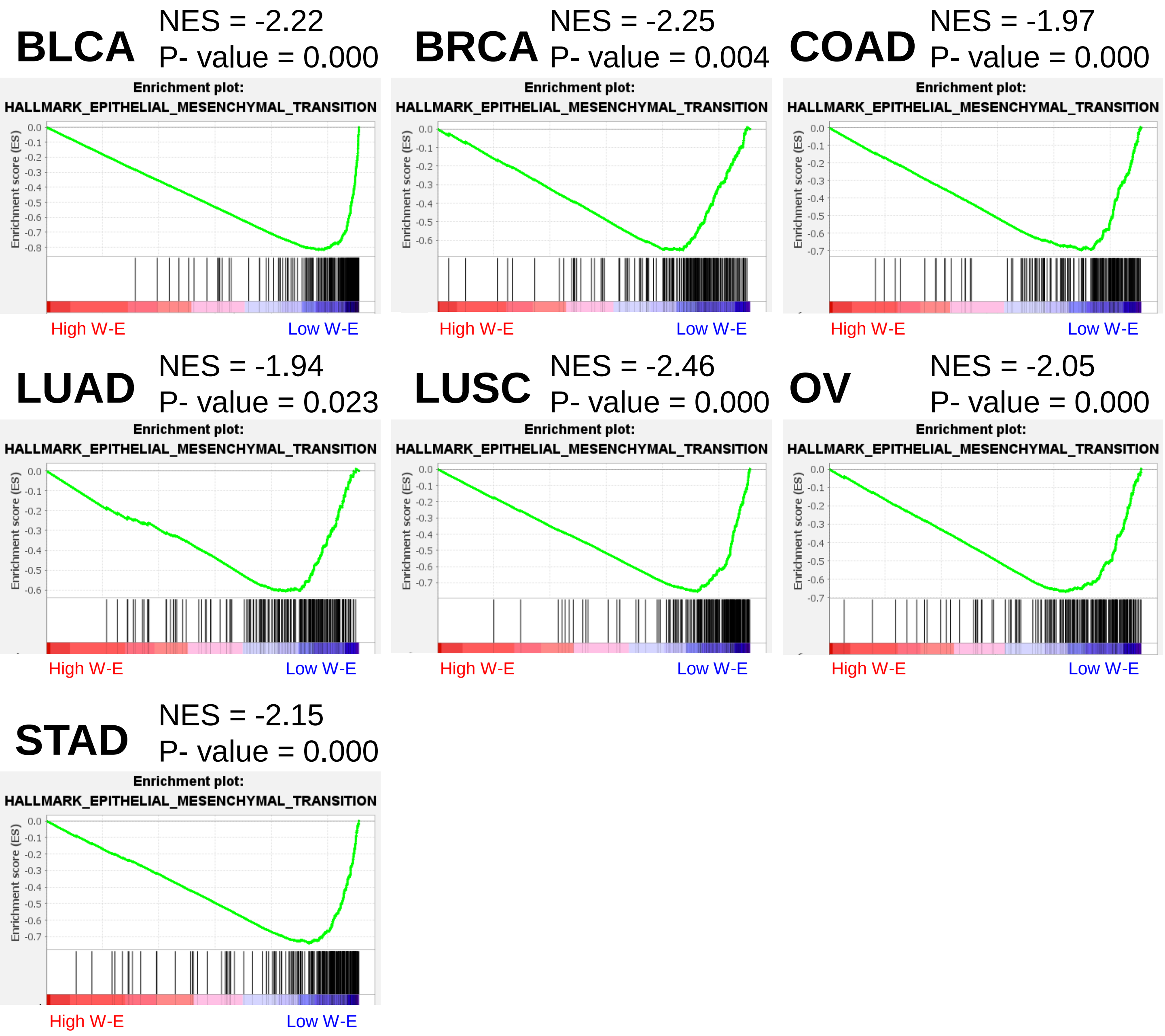

BLCA
NES = -2.22
P- value = 0.000
BRCA
NES = -2.25
P- value = 0.004
COAD
NES = -1.97
P- value = 0.000
LUAD
NES = -1.94
P- value = 0.023
LUSC
NES = -2.46
P- value = 0.000
OV
NES = -2.05
P- value = 0.000
STAD
NES = -2.15
P- value = 0.000
Enrichment plot:
HALLMARK_EPITHELIAL_MESENCHYMAL_TRANSITION
Enrichment score (ES)
High W-E
Low W-E

c

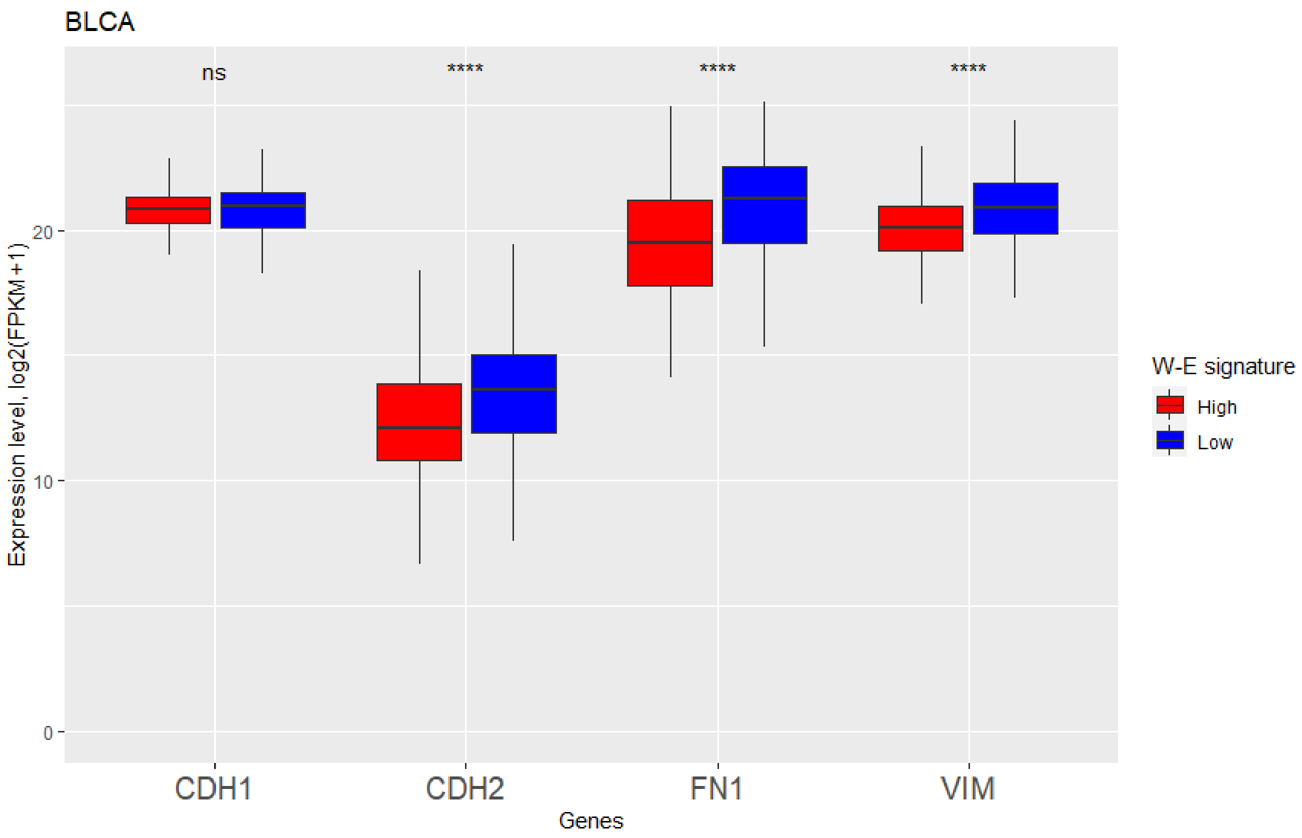
BLCA
ns
****
****
****
Expression level, log2(FPKM+1)
20
10
0
CDH1
CDH2
FN1
VIM
Genes
W-E signature
High
Low

d

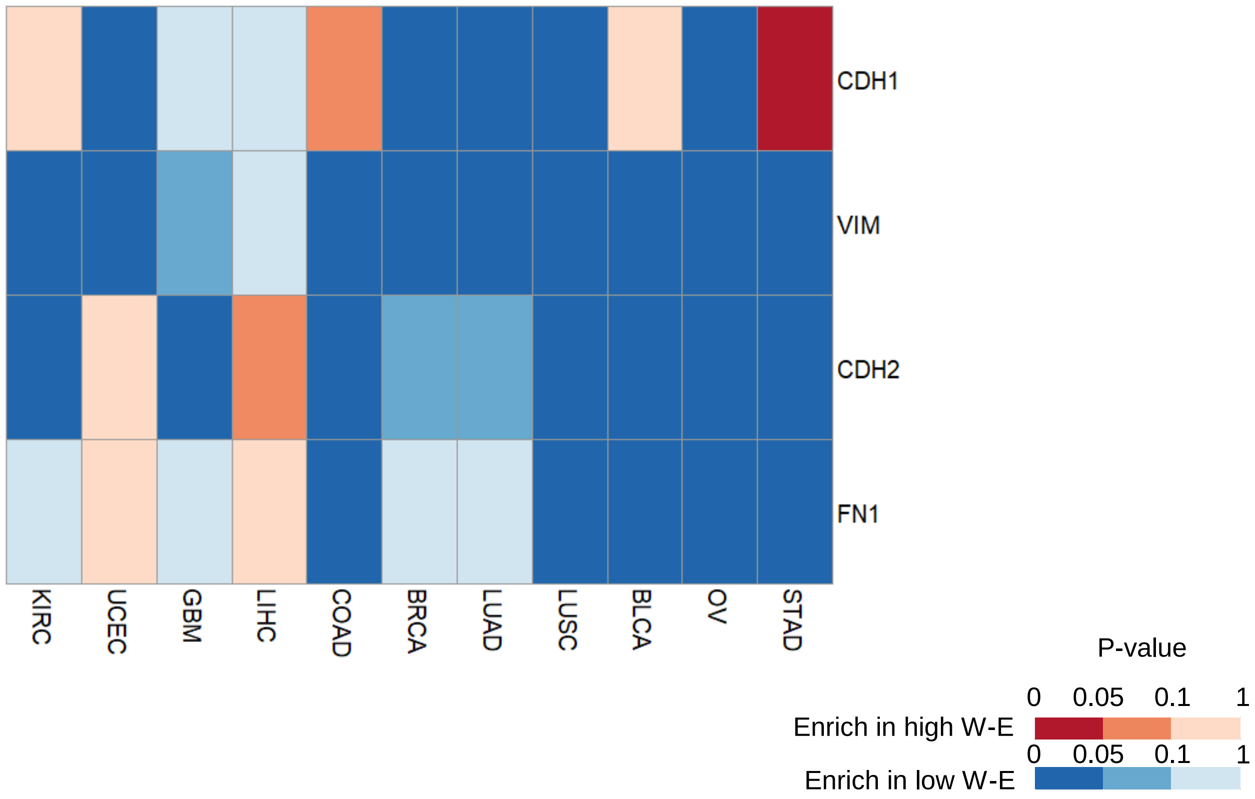
CDH1
VIM
CDH2
FN1
KIRC
UCEC
GBM
LIHC
COAD
BRCA
LUAD
LUSC
BLCA
OV
STAD
P-value
0
0.05
0.1
1
Enrich in high W-E
Enrich in low W-E

Supplementary Figure 11. Expression of genes included in EMT gene sets from bladder cancer samples.

a Heatmap for mRNA expression of genes included in EMT gene sets. Each column stands for an individual cancer sample, and each row denotes a gene. BLCA samples are sorted from left to right by W-E signature in ascending order. At the top of heatmap, W-E signature is presented and discriminated into high and low groups by the color (red: high, blue: low). Among 200 genes in EMT gene set, 105 genes are commonly enriched in BLCA, BRCA, COAD, and LUSC. Z-scores (standard deviation) of 105 genes and m6A writer and eraser are stratified in 6 colors (-3 to 3). **b** GSEA plots of EMT gene sets according to W-E signature in BLCA, BRCA, COAD, LUAD, LUSC, OV, and STAD. Each tumor type's normalized enrichment score and P-value is demonstrated. **c** The expressions of CDH1 (E-cadherin), CDH2 (N-cadherin), FN1 (fibronectin 1), and VIM (vimentin) in bladder cancer are shown in box plot. Red boxes represent samples with high W-E signature and blue boxes represent samples with low W-E signature. P-value from student t-test is shown at the top of plot. **d** Heatmap for the expressions of CDH1, CDH2, FN1, and VIM in 11 cancer types. Each cell represent for its -log(P-value) from student *t*-test. If high W-E signatures have higher gene expression, the color is red. If low W-E signature samples have higher gene expression, the color is blue.

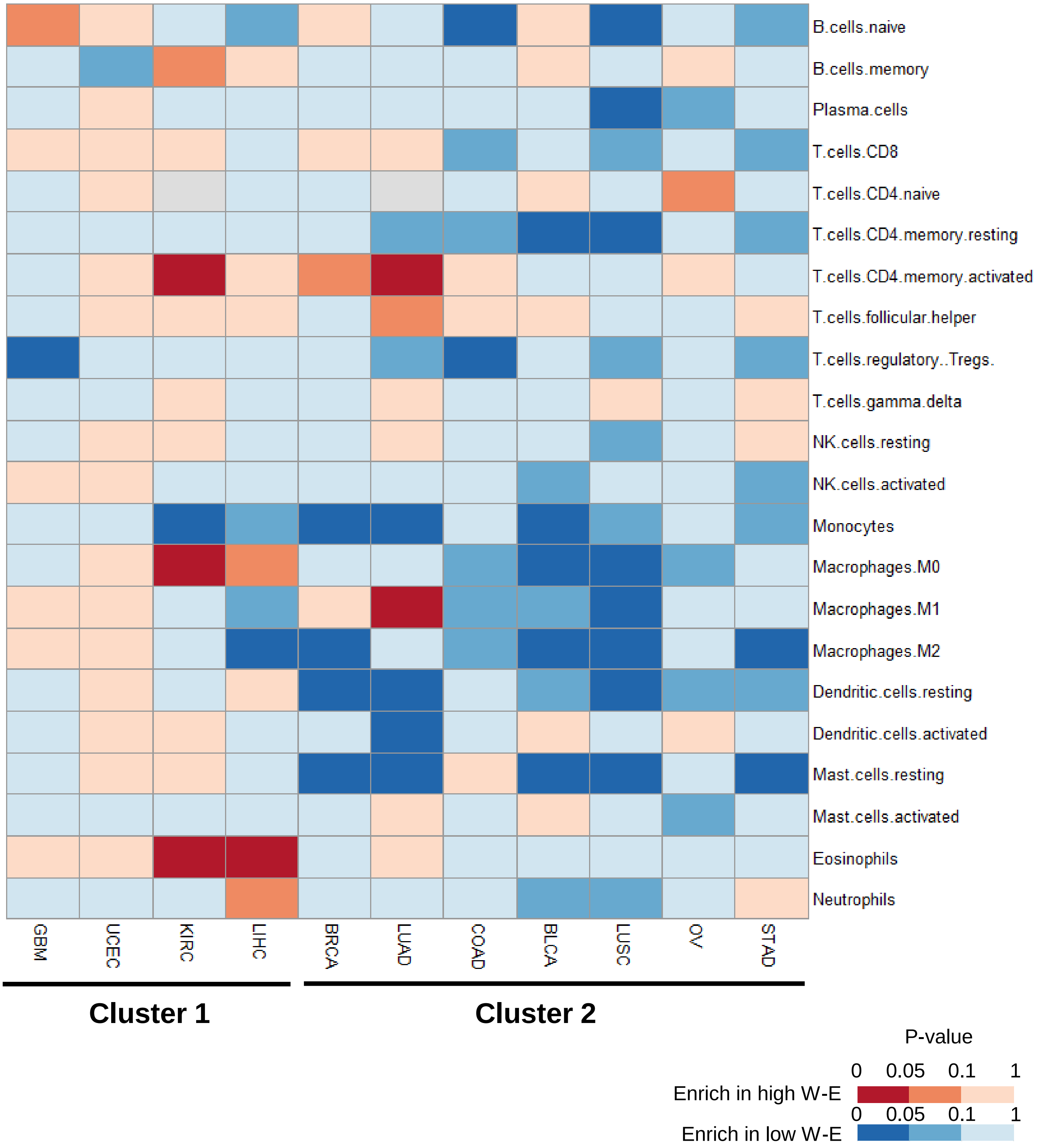
B.cells.naive
B.cells.memory
Plasma.cells
T.cells.CD8
T.cells.CD4.naive
T.cells.CD4.memory.resting
T.cells.CD4.memory.activated
T.cells.follicular.helper
T.cells.regulatory..Tregs.
T.cells.gamma.delta
NK.cells.resting
NK.cells.activated
Monocytes
Macrophages.M0
Macrophages.M1
Macrophages.M2
Dendritic.cells.resting
Dendritic.cells.activated
Mast.cells.resting
Mast.cells.activated
Eosinophils
Neutrophils
GBM
UCEC
KIRC
LIHC
BRCA
LUAD
COAD
BLCA
LUSC
OV
STAD
Cluster 1
Cluster 2
P-value
0
0.05
0.1
1
Enrich in high W-E
0
0.05
0.1
1
Enrich in low W-E

Supplementary Figure 12. Analyzing immune cell proportion in tumors by CIBERSORT.

Heatmap for the difference of immune cell count (absolute mode of CIBERSORT) between samples with high and low m6A W-E signature. Each column stands for an individual cancer type, and each row denotes an immune cell type. Each cell represents -log(P-value) from student *t*-test between groups with high and low W-E signatures. Red means higher count of immune cells in high W-E signature than low W-E signature. Blue means the opposite. Cluster 1 (GBM, UCEC, KIRC, and LIHC), with the exception of GBM, showed poorer survival in patients with high W-E signatures (Fig. 3A). Most of Cluster 2 (COAD, STAD, LUAD, LUSC, BRCA, BLCA, and OV) showed the opposite phenotype (Fig. 3B).

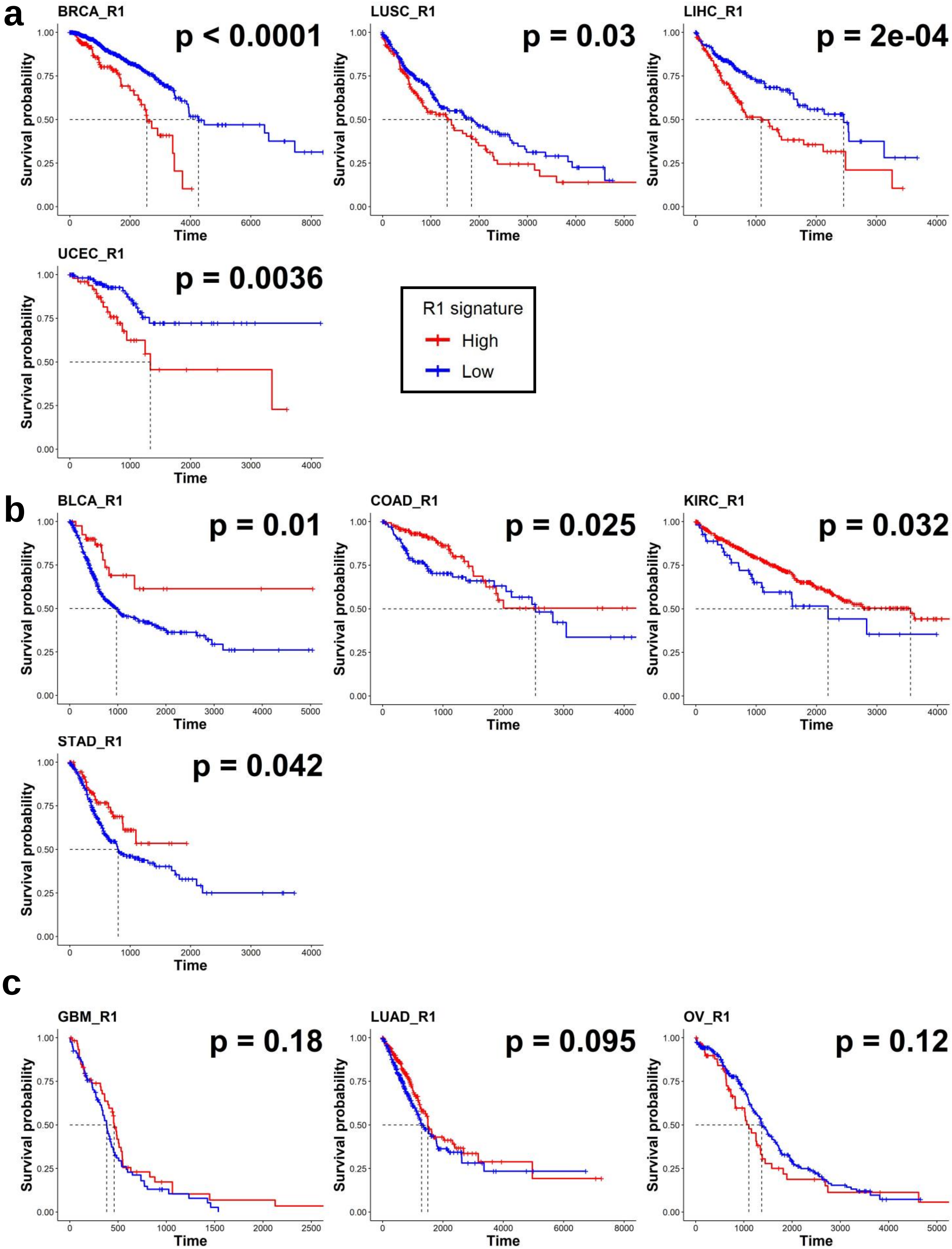
a
BRCA_R1
p < 0.0001
LUSC_R1
p = 0.03
LIHC_R1
p = 2e-04
UCEC_R1
p = 0.0036
R1 signature
High
Low
b
BLCA_R1
p = 0.01
COAD_R1
p = 0.025
KIRC_R1
p = 0.032
STAD_R1
p = 0.042
c
GBM_R1
p = 0.18
LUAD_R1
p = 0.095
OV_R1
p = 0.12
Survival probability
Time

Supplementary Figure 13. Survival analysis according to m6A R1 signature across cancer types.

a-c Kaplan-Meier plots for overall survival of patients with high and low R1 signatures. Red and blue lines represent samples with high and low R1 signatures, respectively. Each median survival and P-value, determined by log rank test, is demonstrated. Tumors that have worse survival in high R1 signatures are demonstrated in (a), tumors that have better survival in high R1 signatures are demonstrated in (b), and tumors that do not show significant difference between high and low R1 signature groups are demonstrated in (c).

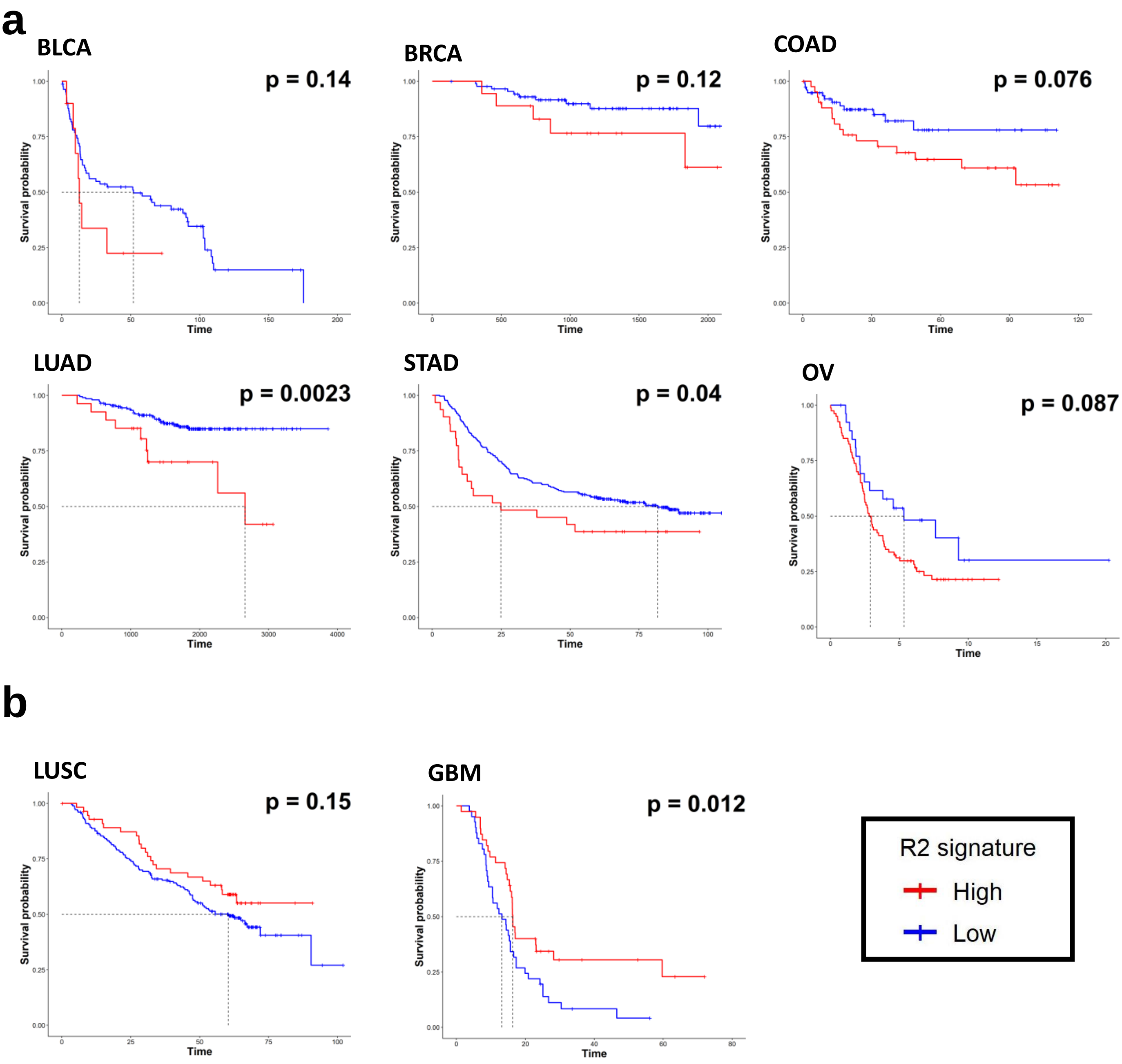

Supplementary Figure 14. Survival analysis according to m6A R2 signature across cancer types in validation cohort.

a, b Kaplan-Meier plots for overall survival of patients with high and low R2 signatures. Gene expression data were analyzed using Gene Expression Omnibus (GEO) database: GSE31684 (BLCA), GSE16446 (BRCA), GSE38832 (COAD), GSE7696 (GBM), GSE31210 (LUAD), GSE157010 (LUSC), GSE26193 (OV) and GSE62254 (STAD). Red and blue lines stand for samples with high and low R2 signature, respectively. Each median survival and P-value, determined by log rank test, is shown. Tumors that have worse survival in high R2 signatures are demonstrated in (a), and tumors that show the tendency of favorable survivals in high R2 signature are demonstrated in (b).

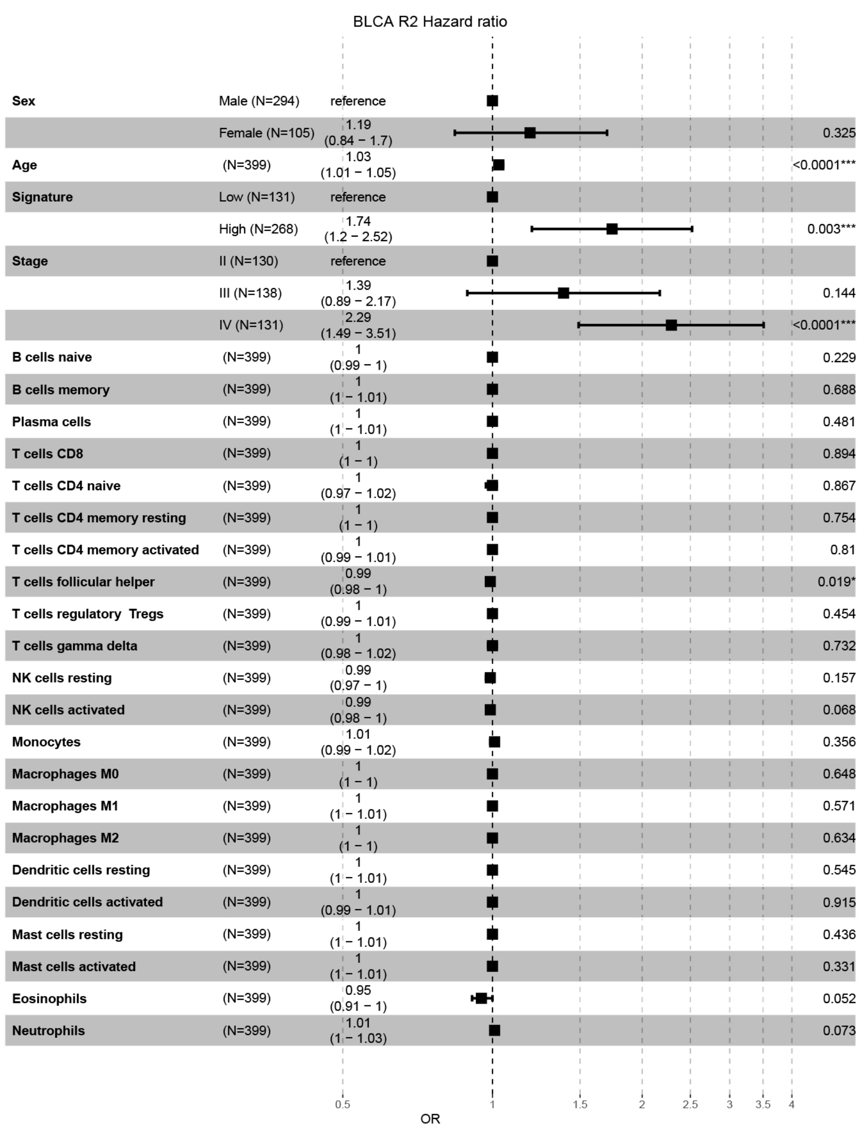
BLCA R2 Hazard ratio
OR

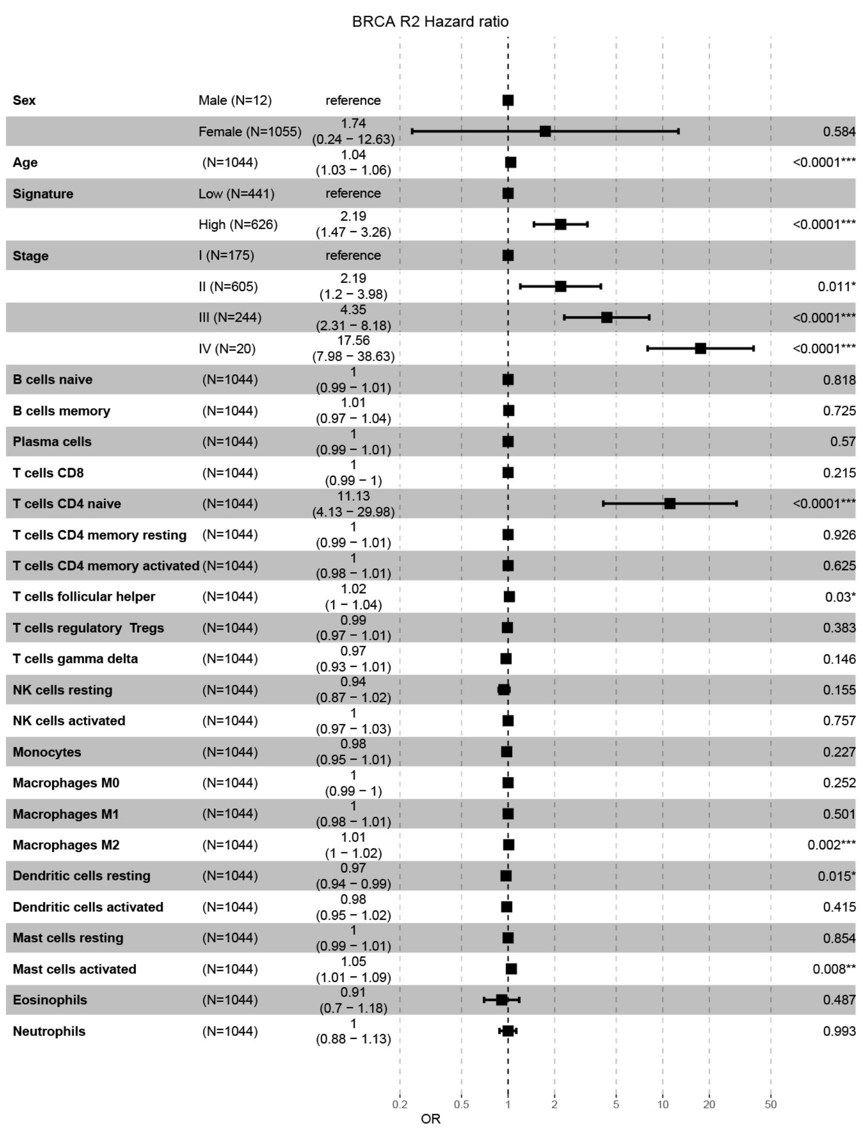
BRCA R2 Hazard ratio
OR

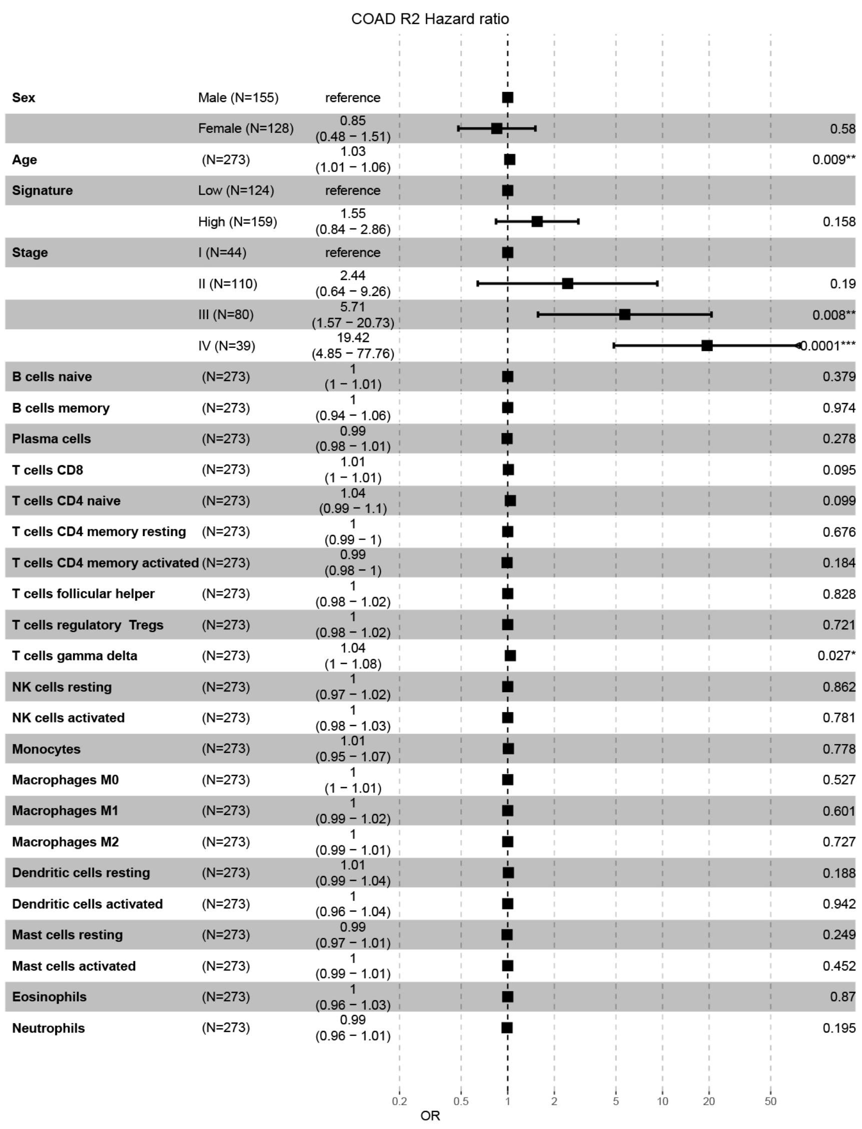
COAD R2 Hazard ratio
OR

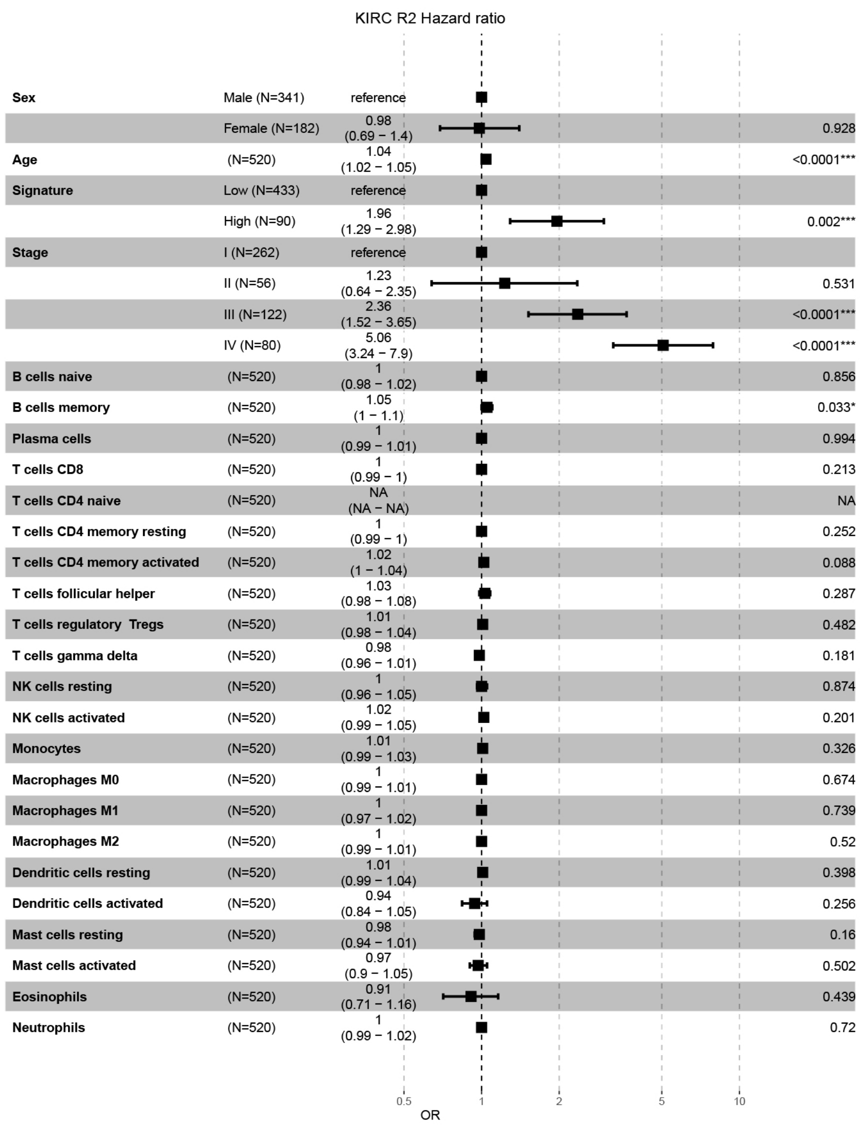
KIRC R2 Hazard ratio
OR

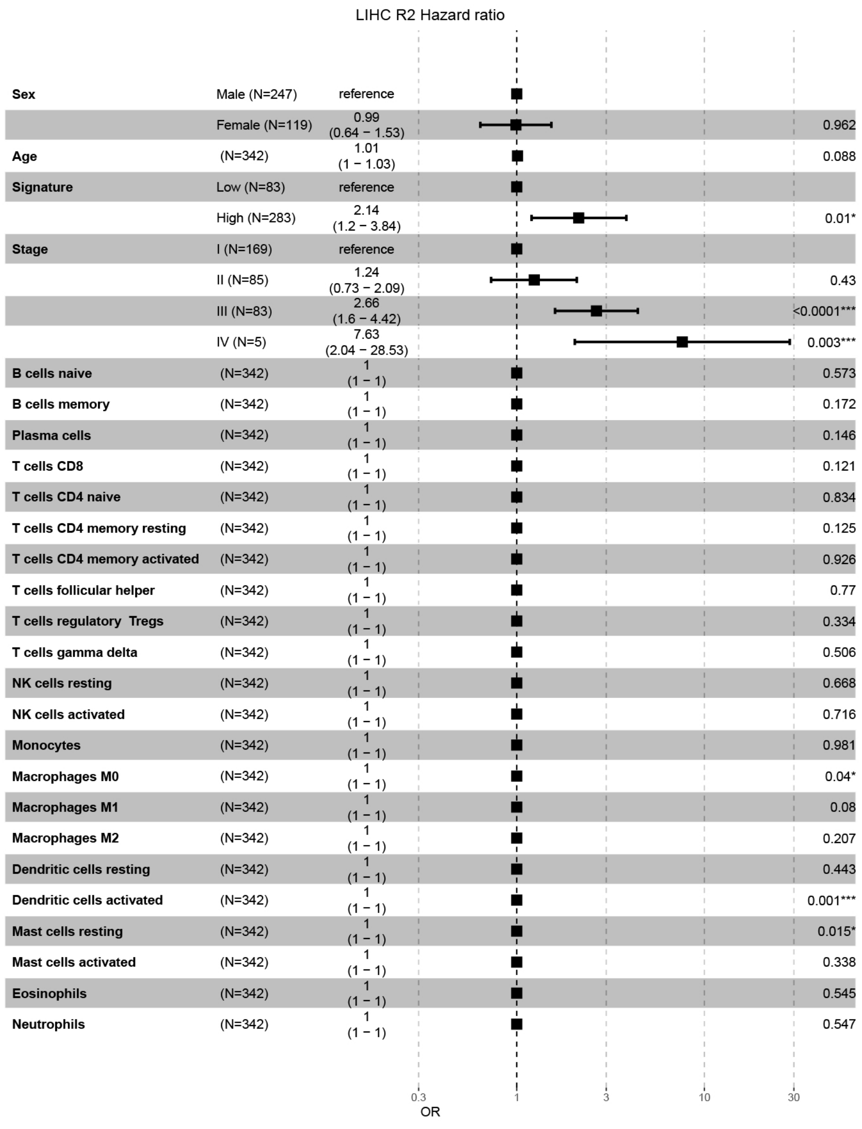
LIHC R2 Hazard ratio
OR

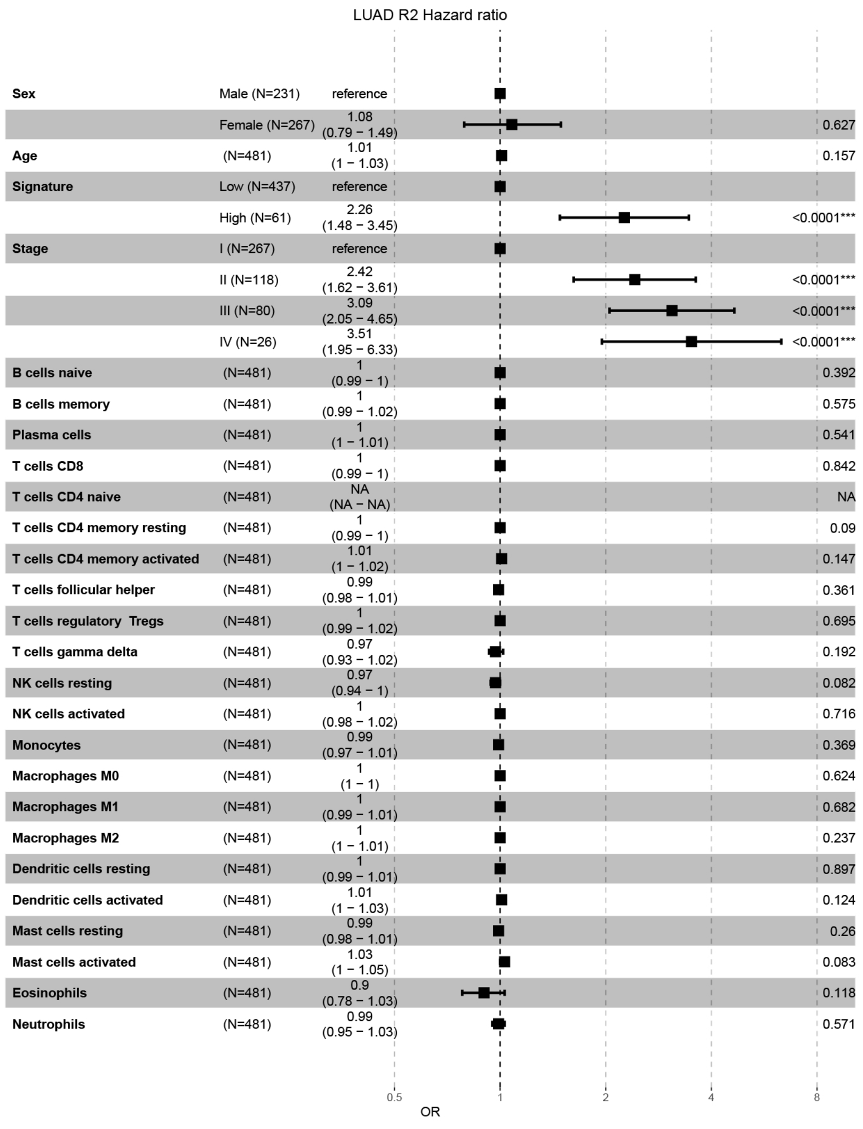
LUAD R2 Hazard ratio
OR

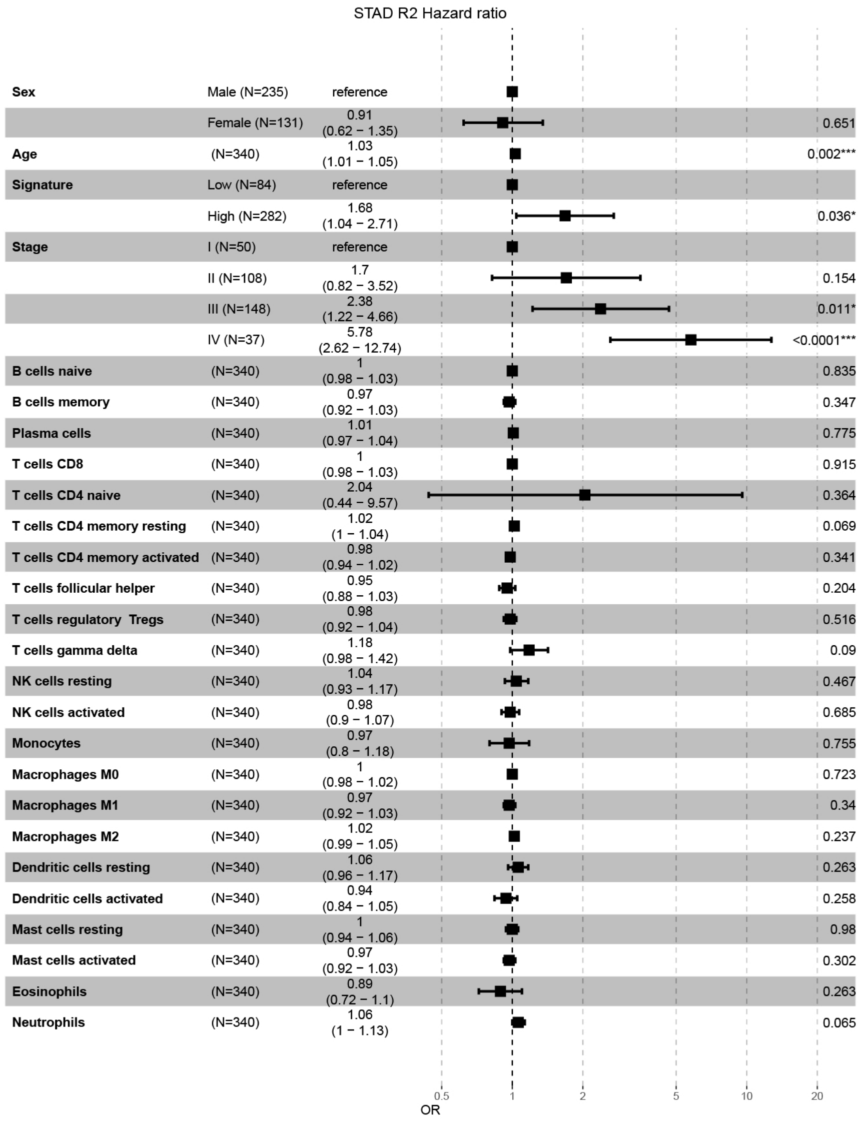
STAD R2 Hazard ratio
Sex Male (N=235) reference
Female (N=131) 0.91 (0.62 − 1.35) 0.651
Age (N=340) 1.03 (1.01 − 1.05) 0.002***
Signature Low (N=84) reference
High (N=282) 1.68 (1.04 − 2.71) 0.036*
Stage I (N=50) reference
II (N=108) 1.7 (0.82 − 3.52) 0.154
III (N=148) 2.38 (1.22 − 4.66) 0.011*
IV (N=37) 5.78 (2.62 − 12.74) <0.0001***
B cells naive (N=340) 1 (0.98 − 1.03) 0.835
B cells memory (N=340) 0.97 (0.92 − 1.03) 0.347
Plasma cells (N=340) 1.01 (0.97 − 1.04) 0.775
T cells CD8 (N=340) 1 (0.98 − 1.03) 0.915
T cells CD4 naive (N=340) 2.04 (0.44 − 9.57) 0.364
T cells CD4 memory resting (N=340) 1.02 (1 − 1.04) 0.069
T cells CD4 memory activated (N=340) 0.98 (0.94 − 1.02) 0.341
T cells follicular helper (N=340) 0.95 (0.88 − 1.03) 0.204
T cells regulatory Tregs (N=340) 0.98 (0.92 − 1.04) 0.516
T cells gamma delta (N=340) 1.18 (0.98 − 1.42) 0.09
NK cells resting (N=340) 1.04 (0.93 − 1.17) 0.467
NK cells activated (N=340) 0.98 (0.9 − 1.07) 0.685
Monocytes (N=340) 0.97 (0.8 − 1.18) 0.755
Macrophages M0 (N=340) 1 (0.98 − 1.02) 0.723
Macrophages M1 (N=340) 0.97 (0.92 − 1.03) 0.34
Macrophages M2 (N=340) 1.02 (0.99 − 1.05) 0.237
Dendritic cells resting (N=340) 1.06 (0.96 − 1.17) 0.263
Dendritic cells activated (N=340) 0.94 (0.84 − 1.05) 0.258
Mast cells resting (N=340) 1 (0.94 − 1.06) 0.98
Mast cells activated (N=340) 0.97 (0.92 − 1.03) 0.302
Eosinophils (N=340) 0.89 (0.72 − 1.1) 0.263
Neutrophils (N=340) 1.06 (1 − 1.13) 0.065
0.5 1 2 5 10 20
OR

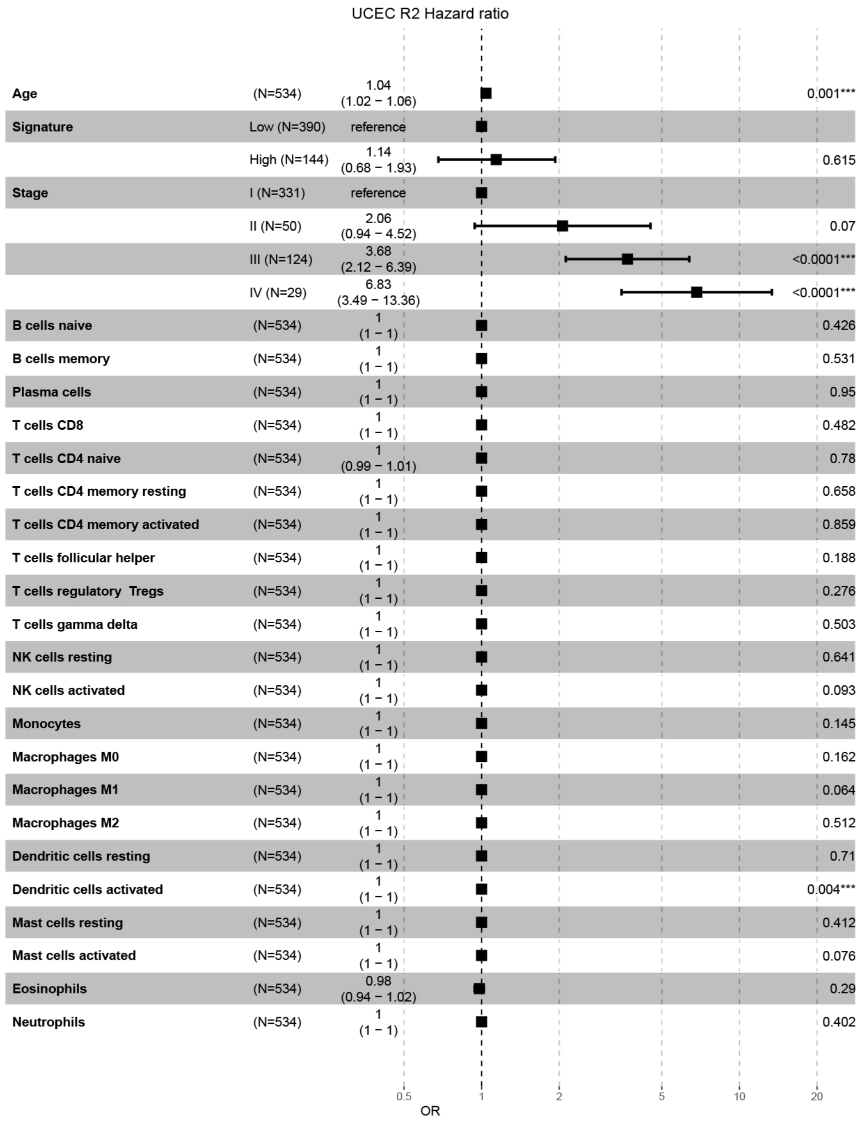
UCEC R2 Hazard ratio
Age (N=534) 1.04 (1.02 − 1.06) 0.001***
Signature Low (N=390) reference
High (N=144) 1.14 (0.68 − 1.93) 0.615
Stage I (N=331) reference
II (N=50) 2.06 (0.94 − 4.52) 0.07
III (N=124) 3.68 (2.12 − 6.39) <0.0001***
IV (N=29) 6.83 (3.49 − 13.36) <0.0001***
B cells naive (N=534) 1 (1 − 1) 0.426
B cells memory (N=534) 1 (1 − 1) 0.531
Plasma cells (N=534) 1 (1 − 1) 0.95
T cells CD8 (N=534) 1 (1 − 1) 0.482
T cells CD4 naive (N=534) 1 (0.99 − 1.01) 0.78
T cells CD4 memory resting (N=534) 1 (1 − 1) 0.658
T cells CD4 memory activated (N=534) 1 (1 − 1) 0.859
T cells follicular helper (N=534) 1 (1 − 1) 0.188
T cells regulatory Tregs (N=534) 1 (1 − 1) 0.276
T cells gamma delta (N=534) 1 (1 − 1) 0.503
NK cells resting (N=534) 1 (1 − 1) 0.641
NK cells activated (N=534) 1 (1 − 1) 0.093
Monocytes (N=534) 1 (1 − 1) 0.145
Macrophages M0 (N=534) 1 (1 − 1) 0.162
Macrophages M1 (N=534) 1 (1 − 1) 0.064
Macrophages M2 (N=534) 1 (1 − 1) 0.512
Dendritic cells resting (N=534) 1 (1 − 1) 0.71
Dendritic cells activated (N=534) 1 (1 − 1) 0.004***
Mast cells resting (N=534) 1 (1 − 1) 0.412
Mast cells activated (N=534) 1 (1 − 1) 0.076
Eosinophils (N=534) 0.98 (0.94 − 1.02) 0.29
Neutrophils (N=534) 1 (1 − 1) 0.402
0.5 1 2 5 10 20
OR

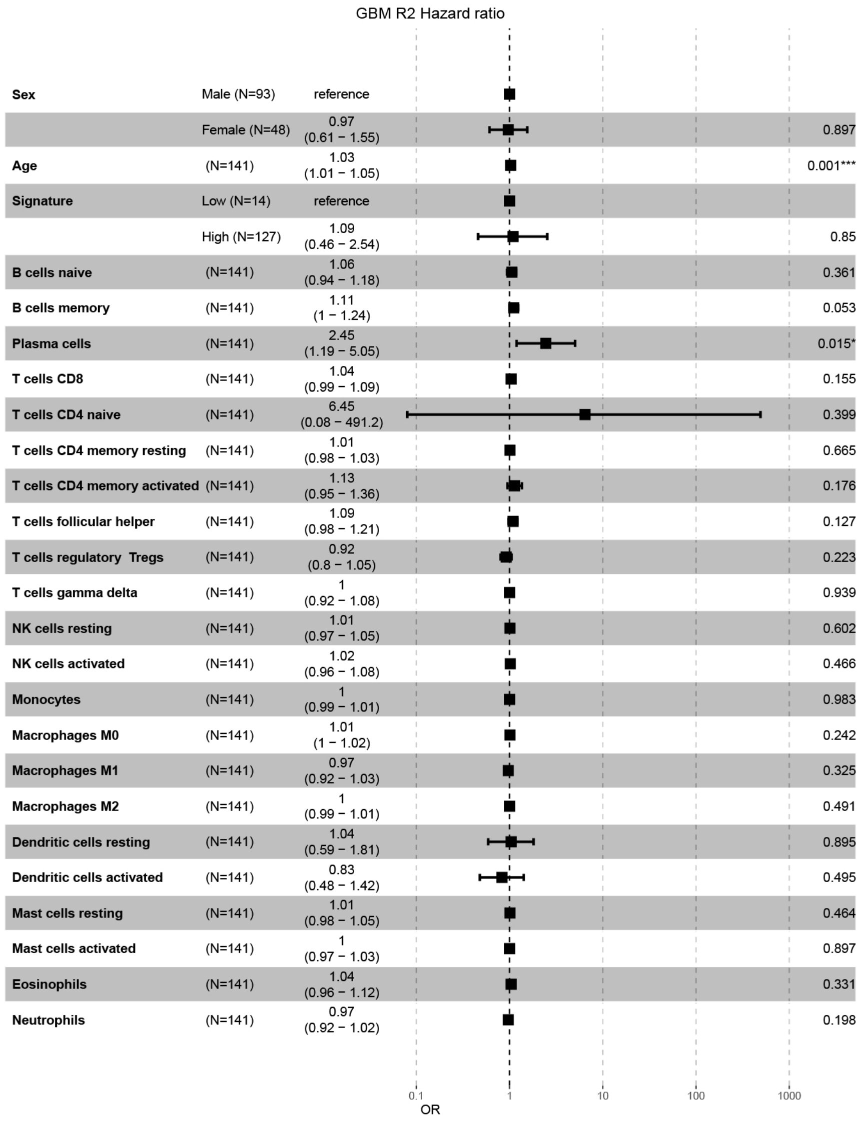
GBM R2 Hazard ratio
Sex Male (N=93) reference
Female (N=48) 0.97 (0.61 − 1.55) 0.897
Age (N=141) 1.03 (1.01 − 1.05) 0.001***
Signature Low (N=14) reference
High (N=127) 1.09 (0.46 − 2.54) 0.85
B cells naive (N=141) 1.06 (0.94 − 1.18) 0.361
B cells memory (N=141) 1.11 (1 − 1.24) 0.053
Plasma cells (N=141) 2.45 (1.19 − 5.05) 0.015*
T cells CD8 (N=141) 1.04 (0.99 − 1.09) 0.155
T cells CD4 naive (N=141) 6.45 (0.08 − 491.2) 0.399
T cells CD4 memory resting (N=141) 1.01 (0.98 − 1.03) 0.665
T cells CD4 memory activated (N=141) 1.13 (0.95 − 1.36) 0.176
T cells follicular helper (N=141) 1.09 (0.98 − 1.21) 0.127
T cells regulatory Tregs (N=141) 0.92 (0.8 − 1.05) 0.223
T cells gamma delta (N=141) 1 (0.92 − 1.08) 0.939
NK cells resting (N=141) 1.01 (0.97 − 1.05) 0.602
NK cells activated (N=141) 1.02 (0.96 − 1.08) 0.466
Monocytes (N=141) 1 (0.99 − 1.01) 0.983
Macrophages M0 (N=141) 1.01 (1 − 1.02) 0.242
Macrophages M1 (N=141) 0.97 (0.92 − 1.03) 0.325
Macrophages M2 (N=141) 1 (0.99 − 1.01) 0.491
Dendritic cells resting (N=141) 1.04 (0.59 − 1.81) 0.895
Dendritic cells activated (N=141) 0.83 (0.48 − 1.42) 0.495
Mast cells resting (N=141) 1.01 (0.98 − 1.05) 0.464
Mast cells activated (N=141) 1 (0.97 − 1.03) 0.897
Eosinophils (N=141) 1.04 (0.96 − 1.12) 0.331
Neutrophils (N=141) 0.97 (0.92 − 1.02) 0.198
0.1 1 10 100 1000
OR

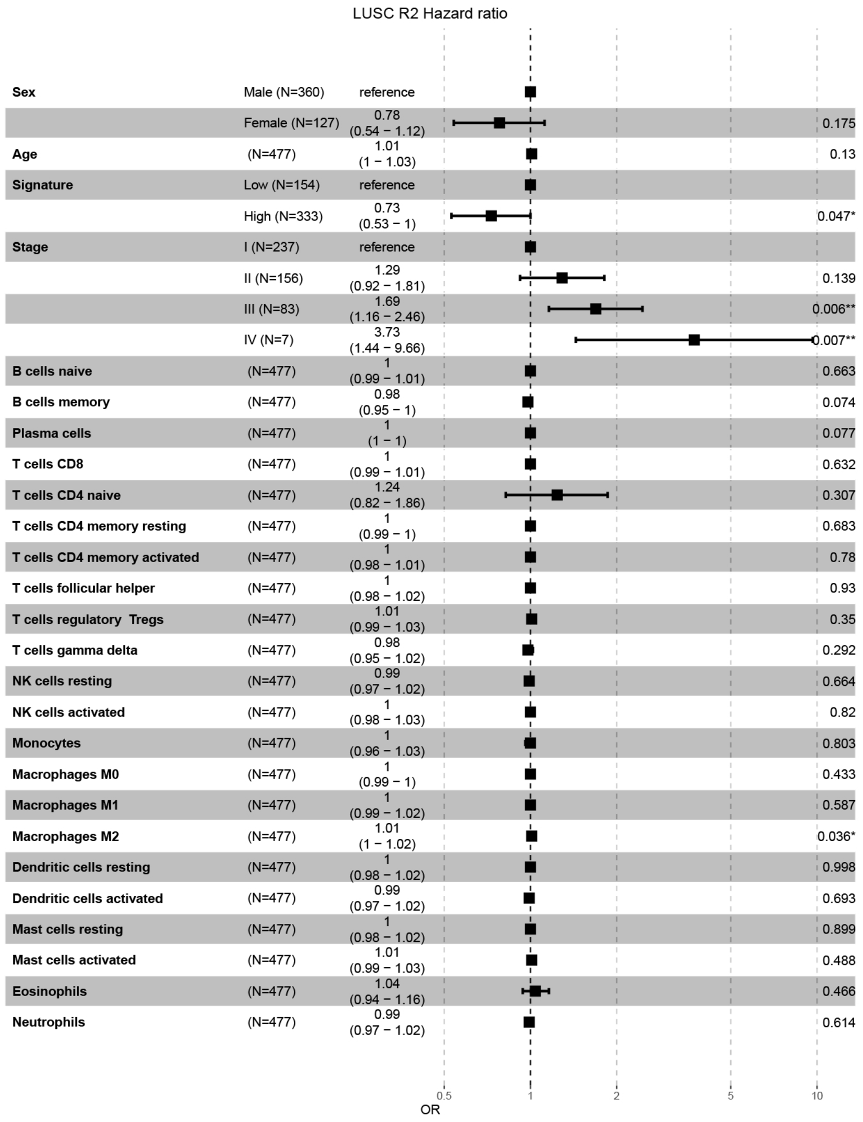
LUSC R2 Hazard ratio
Sex Male (N=360) reference
Female (N=127) 0.78 (0.54 − 1.12) 0.175
Age (N=477) 1.01 (1 − 1.03) 0.13
Signature Low (N=154) reference
High (N=333) 0.73 (0.53 − 1) 0.047*
Stage I (N=237) reference
II (N=156) 1.29 (0.92 − 1.81) 0.139
III (N=83) 1.69 (1.16 − 2.46) 0.006**
IV (N=7) 3.73 (1.44 − 9.66) 0.007**
B cells naive (N=477) 1 (0.99 − 1.01) 0.663
B cells memory (N=477) 0.98 (0.95 − 1) 0.074
Plasma cells (N=477) 1 (1 − 1) 0.077
T cells CD8 (N=477) 1 (0.99 − 1.01) 0.632
T cells CD4 naive (N=477) 1.24 (0.82 − 1.86) 0.307
T cells CD4 memory resting (N=477) 1 (0.99 − 1) 0.683
T cells CD4 memory activated (N=477) 1 (0.98 − 1.01) 0.78
T cells follicular helper (N=477) 1 (0.98 − 1.02) 0.93
T cells regulatory Tregs (N=477) 1.01 (0.99 − 1.03) 0.35
T cells gamma delta (N=477) 0.98 (0.95 − 1.02) 0.292
NK cells resting (N=477) 0.99 (0.97 − 1.02) 0.664
NK cells activated (N=477) 1 (0.98 − 1.03) 0.82
Monocytes (N=477) 1 (0.96 − 1.03) 0.803
Macrophages M0 (N=477) 1 (0.99 − 1) 0.433
Macrophages M1 (N=477) 1 (0.99 − 1.02) 0.587
Macrophages M2 (N=477) 1.01 (1 − 1.02) 0.036*
Dendritic cells resting (N=477) 1 (0.98 − 1.02) 0.998
Dendritic cells activated (N=477) 0.99 (0.97 − 1.02) 0.693
Mast cells resting (N=477) 1 (0.98 − 1.02) 0.899
Mast cells activated (N=477) 1.01 (0.99 − 1.03) 0.488
Eosinophils (N=477) 1.04 (0.94 − 1.16) 0.466
Neutrophils (N=477) 0.99 (0.97 − 1.02) 0.614
0.5 1 2 5 10
OR

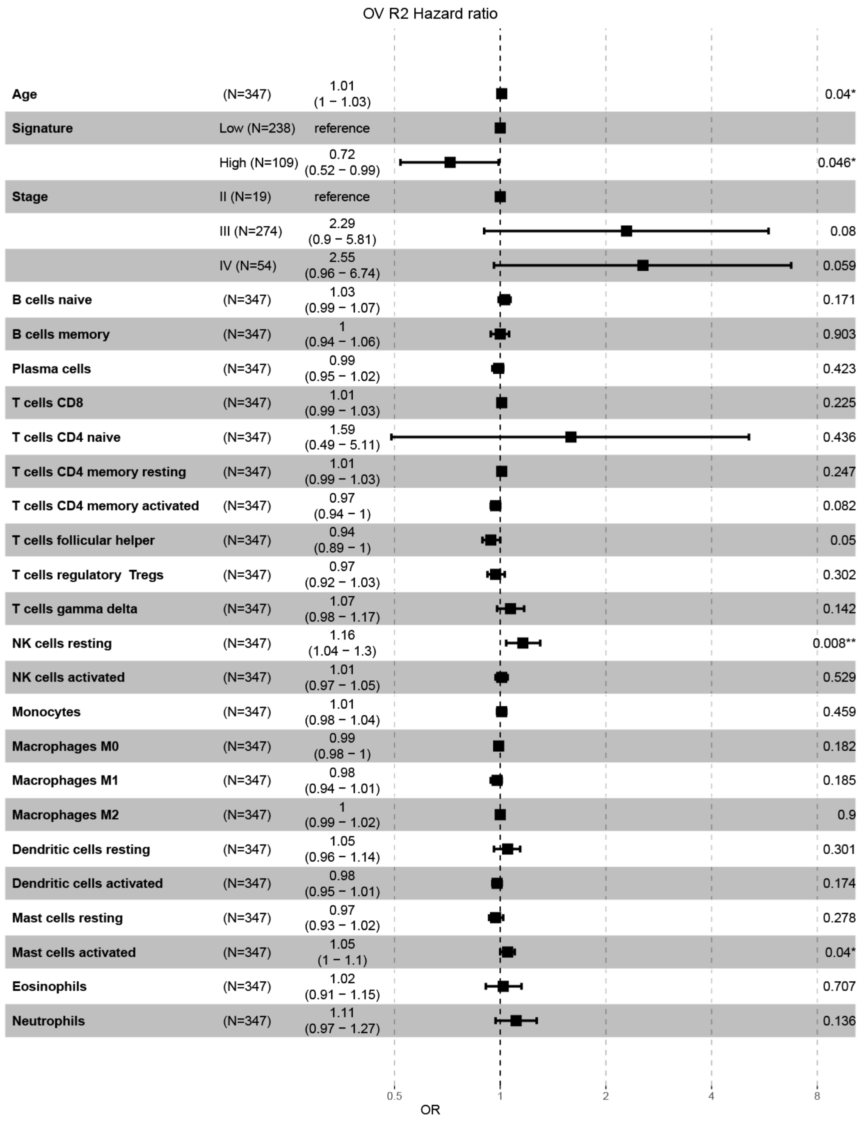
OV R2 Hazard ratio
Age (N=347) 1.01 (1 − 1.03) 0.04*
Signature Low (N=238) reference
High (N=109) 0.72 (0.52 − 0.99) 0.046*
Stage II (N=19) reference
III (N=274) 2.29 (0.9 − 5.81) 0.08
IV (N=54) 2.55 (0.96 − 6.74) 0.059
B cells naive (N=347) 1.03 (0.99 − 1.07) 0.171
B cells memory (N=347) 1 (0.94 − 1.06) 0.903
Plasma cells (N=347) 0.99 (0.95 − 1.02) 0.423
T cells CD8 (N=347) 1.01 (0.99 − 1.03) 0.225
T cells CD4 naive (N=347) 1.59 (0.49 − 5.11) 0.436
T cells CD4 memory resting (N=347) 1.01 (0.99 − 1.03) 0.247
T cells CD4 memory activated (N=347) 0.97 (0.94 − 1) 0.082
T cells follicular helper (N=347) 0.94 (0.89 − 1) 0.05
T cells regulatory Tregs (N=347) 0.97 (0.92 − 1.03) 0.302
T cells gamma delta (N=347) 1.07 (0.98 − 1.17) 0.142
NK cells resting (N=347) 1.16 (1.04 − 1.3) 0.008**
NK cells activated (N=347) 1.01 (0.97 − 1.05) 0.529
Monocytes (N=347) 1.01 (0.98 − 1.04) 0.459
Macrophages M0 (N=347) 0.99 (0.98 − 1) 0.182
Macrophages M1 (N=347) 0.98 (0.94 − 1.01) 0.185
Macrophages M2 (N=347) 1 (0.99 − 1.02) 0.9
Dendritic cells resting (N=347) 1.05 (0.96 − 1.14) 0.301
Dendritic cells activated (N=347) 0.98 (0.95 − 1.01) 0.174
Mast cells resting (N=347) 0.97 (0.93 − 1.02) 0.278
Mast cells activated (N=347) 1.05 (1 − 1.1) 0.04*
Eosinophils (N=347) 1.02 (0.91 − 1.15) 0.707
Neutrophils (N=347) 1.11 (0.97 − 1.27) 0.136
0.5 1 2 4 8
OR

Supplementary Figure 15. Multivariate cox regression model between high and low m6A R2 signatures across cancer types.

For multivariate cox regression model, we used sex, age, stage, the amounts of immune cells, and m6A R2 signature. Some variates that cannot include in analysis such as stage in GBM or gender in UCEC were excluded. Because there are only 19 and 1 patients with stage I bladder and ovarian cancer, respectively, stage I are excluded from the analysis in both types of tumor. Forrest plots of variates are demonstrated.

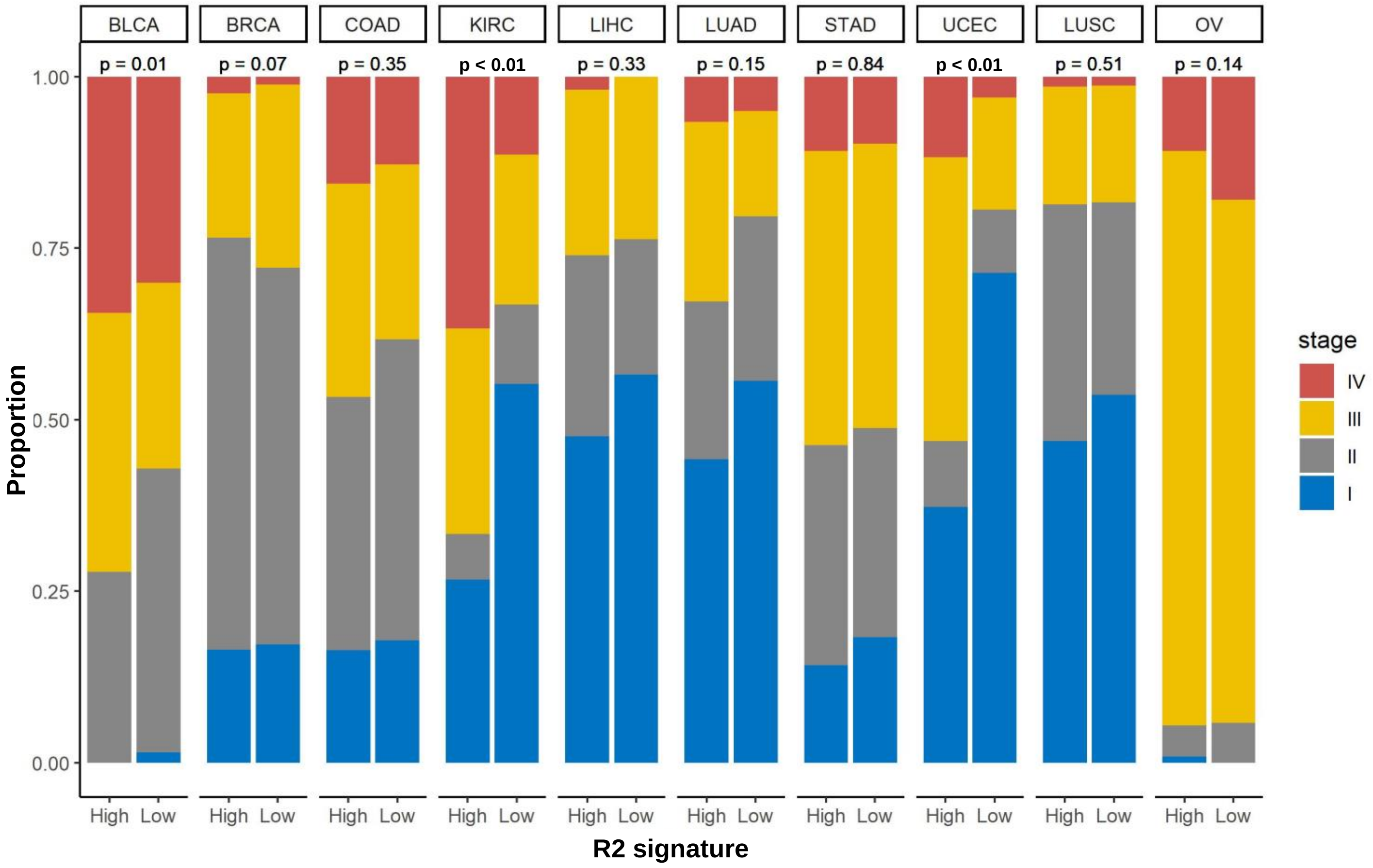

Supplementary Figure 16. Distribution of disease stage in high and low m6A R2 signatures across cancer types.

From the bottom of plot, blue, gray, yellow, and red stand for stage I, II, III and IV. Substages are integrated. High R2 signature is on left side and low R2 signature is on right side of plot. P-values from chi square test are demonstrated at the top of plot.

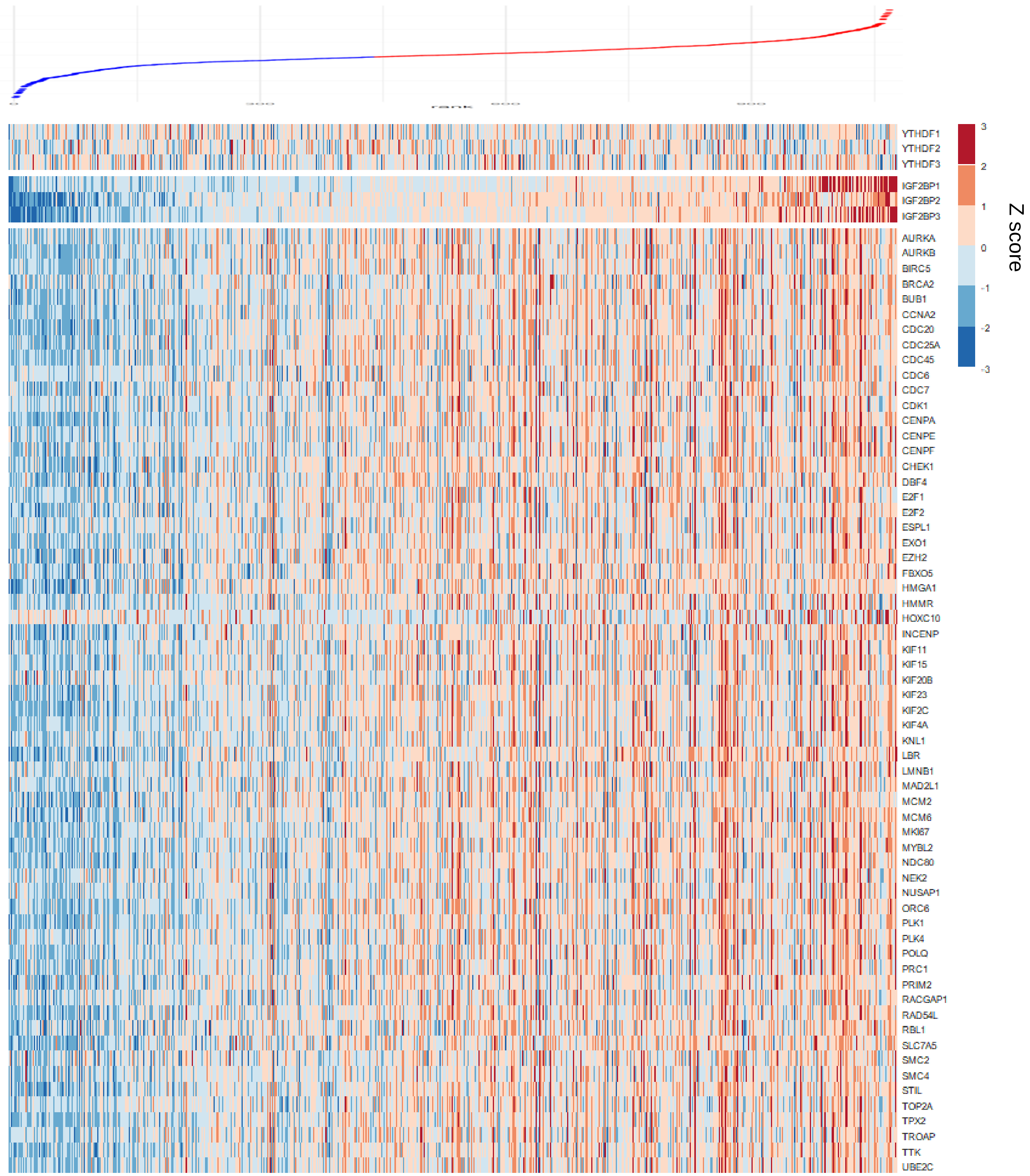

rank
YTHDF1
YTHDF2
YTHDF3
IGF2BP1
IGF2BP2
IGF2BP3
AURKA
AURKB
BIRC5
BRCA2
BUB1
CCNA2
CDC20
CDC25A
CDC45
CDC6
CDC7
CDK1
CENPA
CENPE
CENPF
CHEK1
DBF4
E2F1
E2F2
ESPL1
EXO1
EZH2
FBXO5
HMGA1
HMMR
HOXC10
INCENP
KIF11
KIF15
KIF20B
KIF23
KIF2C
KIF4A
KNL1
LBR
LMNB1
MAD2L1
MCM2
MCM6
MKI67
MYBL2
NDC80
NEK2
NUSAP1
ORC6
PLK1
PLK4
POLQ
PRC1
PRIM2
RACGAP1
RAD54L
RBL1
SLC7A5
SMC2
SMC4
STIL
TOP2A
TPX2
TROAP
TTK
UBE2C
3
2
1
0
-1
-2
-3
Z score

Supplementary Figure 17. Heatmap for mRNA expression of genes included in G2M checkpoint gene sets from breast cancer samples.

Each column stands for an individual cancer sample, and each row denotes a gene. Among 200 genes in G2M checkpoint gene set, 62 genes are commonly enriched in BRCA, BLCA, KIRC, and LUAD. Z-scores (standard deviation) of 62 genes and m6A reader genes are stratified in 6 colors (-3 to 3). At the top of heatmap, R2 signature is presented and discriminated into high and low groups by the color (red: high, blue: low). From left to right, breast cancer samples are sorted by R2 signatures in ascending order.

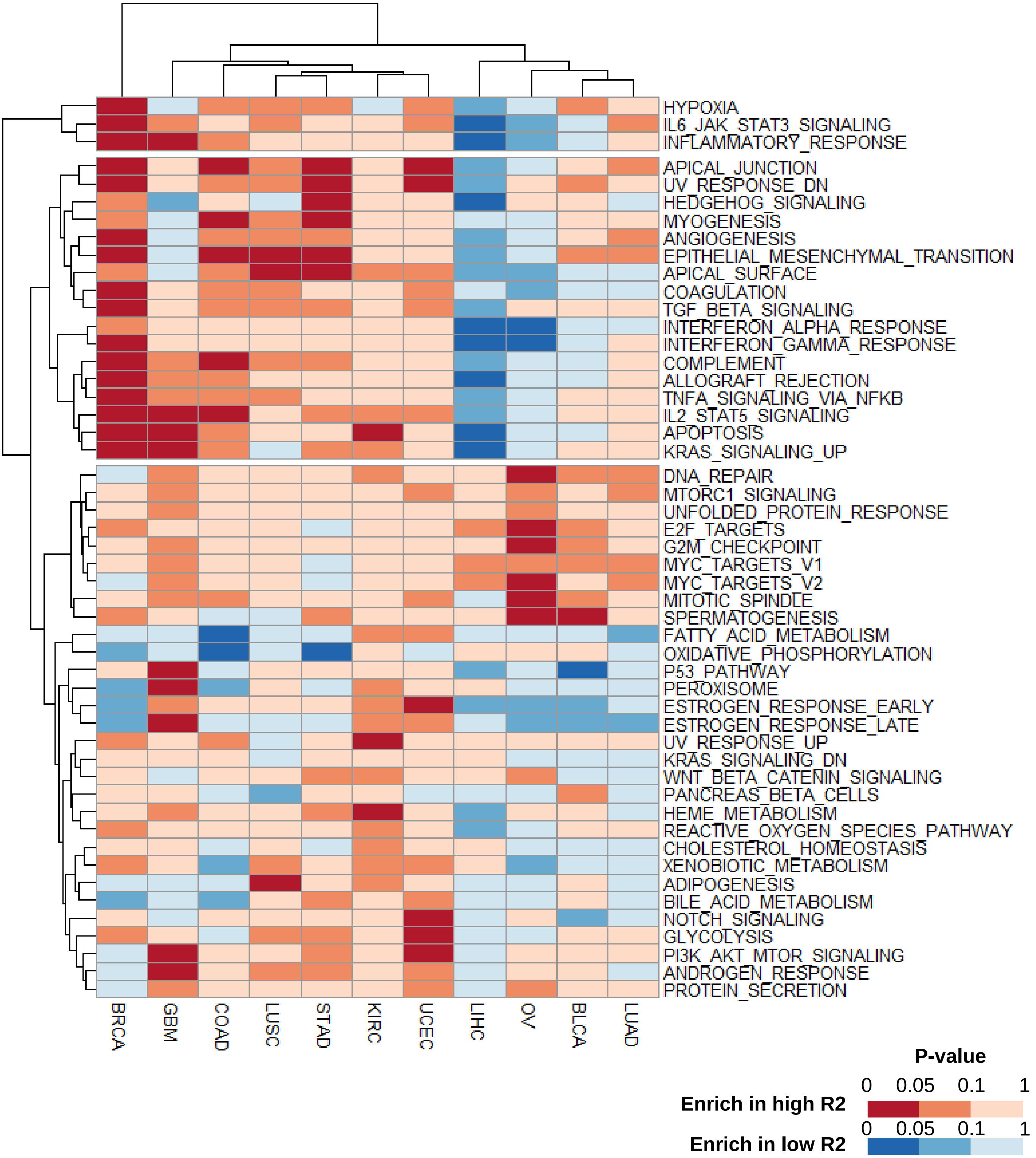

Supplementary Figure 18. Heatmap of gene set enrichment analysis (GSEA) between cell lines with high and low R2 signatures.

Each column stands for an individual cancer type, and each row denotes an enriched hallmark gene set from Molecular Signatures Database (https://www.gsea-msigdb.org/gsea/msigdb/index.jsp). Gene sets enriched with high R2 signature groups are colored with red, and gene sets that are enriched with low R2 signature groups are colored with blue. The lower the P-value, the deeper the color that is shown.

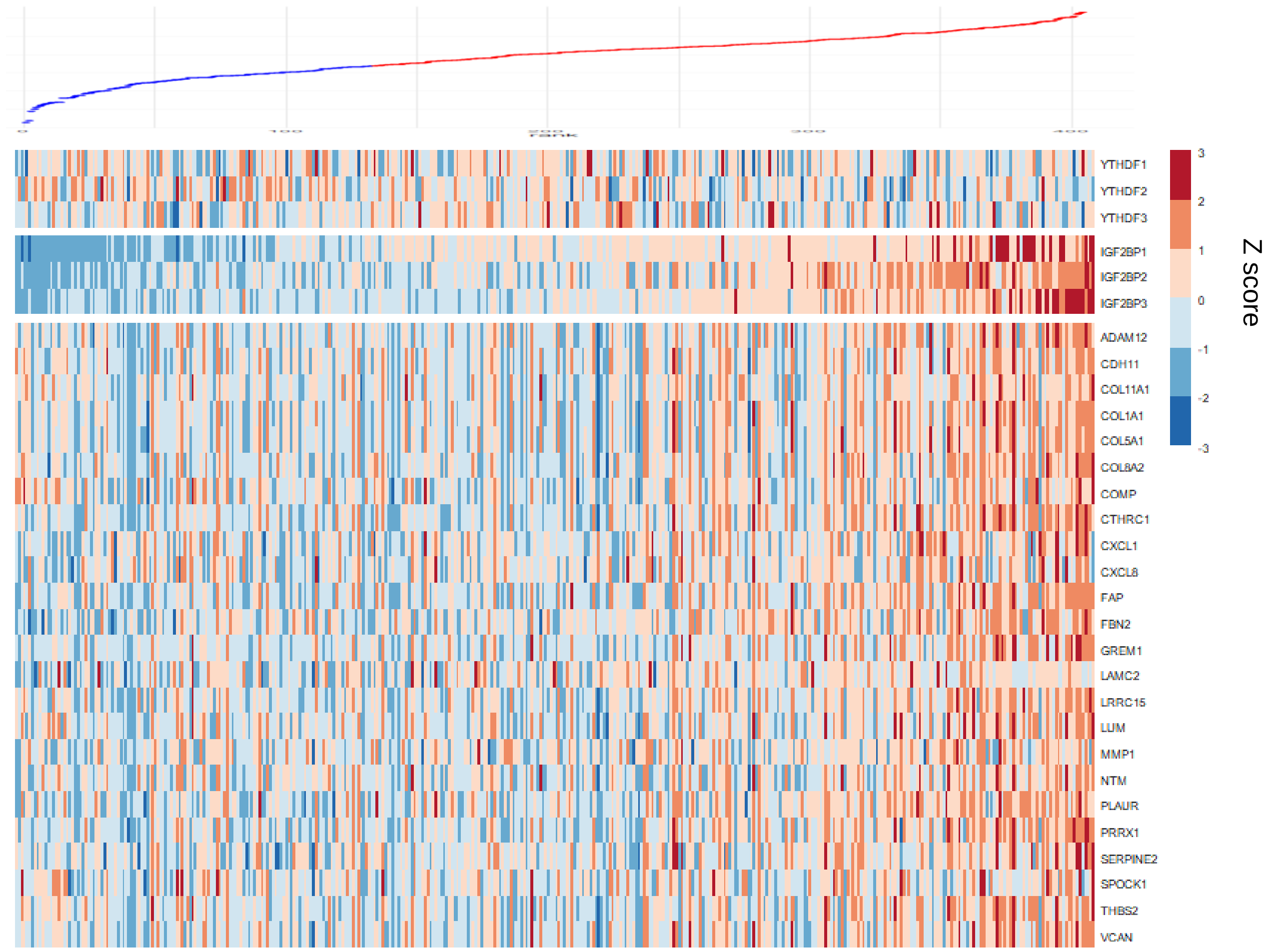

Supplementary Figure 19. Heatmap for mRNA expression of genes included in EMT gene sets from bladder cancer samples.

Each column stands for an individual cancer sample, and each row denotes a gene. Among 200 genes in EMT gene set, 24 genes are commonly enriched in BLCA, BRCA, and KIRC. Z-scores (standard deviation) of 24 genes and m6A reader genes are stratified in 6 colors (-3 to 3). At the top of heatmap, R2 signature is presented and discriminated into high and low groups by the color (red: high, blue: low). From left to right, bladder cancer samples are sorted by R2 signatures in ascending order.

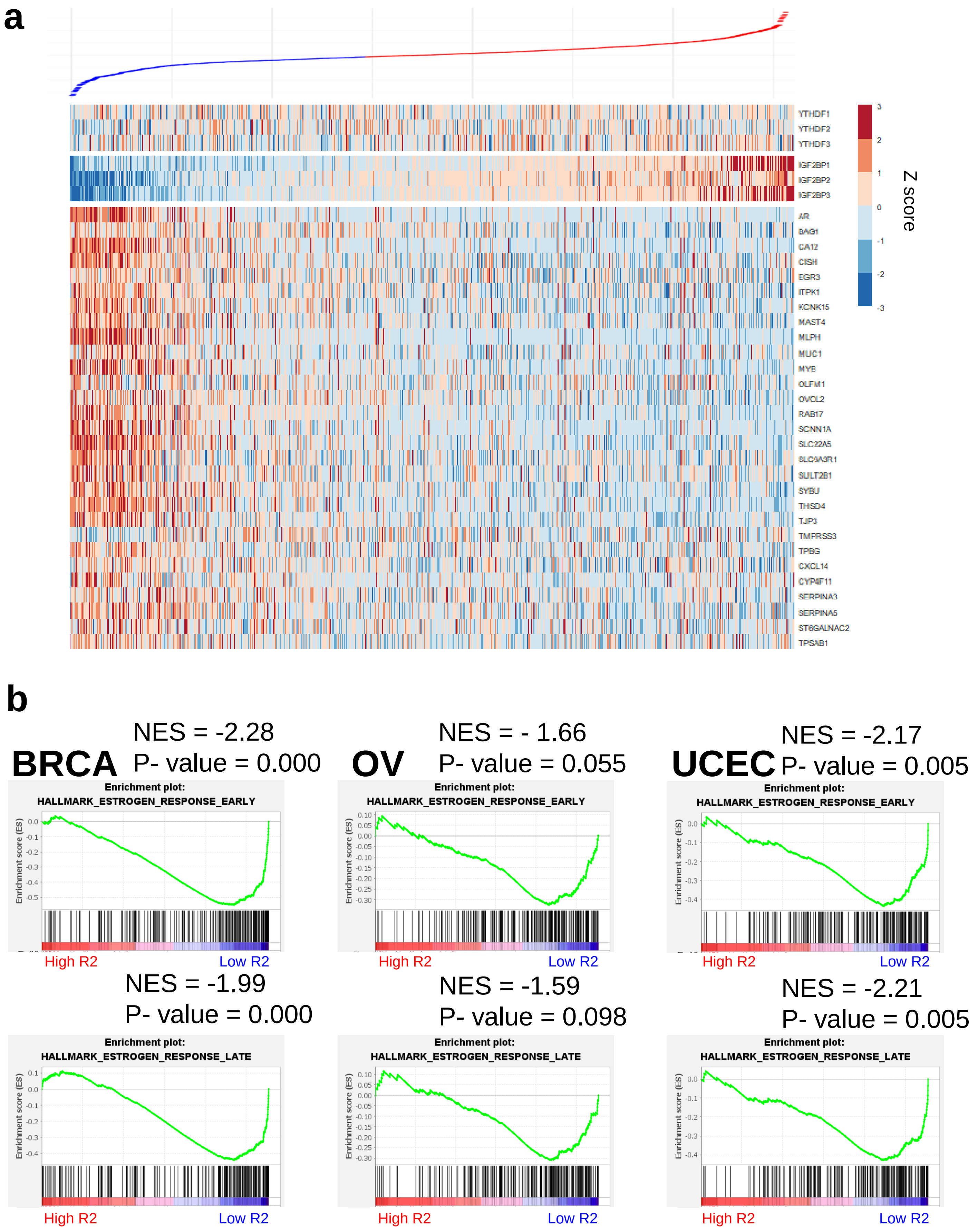
a
YTHDF1
YTHDF2
YTHDF3
IGF2BP1
IGF2BP2
IGF2BP3
AR
BAG1
CA12
CISH
EGR3
ITPK1
KCNK15
MAST4
MLPH
MUC1
MYB
OLFM1
OVOL2
RAB17
SCNN1A
SLC22A5
SLC9A3R1
SULT2B1
SYBU
THSD4
TJP3
TMPRSS3
TPBG
CXCL14
CYP4F11
SERPINA3
SERPINA5
ST6GALNAC2
TPSAB1
Z score
3
2
1
0
-1
-2
-3
b
BRCA
NES = -2.28
P- value = 0.000
Enrichment plot: HALLMARK_ESTROGEN_RESPONSE_EARLY
Enrichment score (ES)
High R2
Low R2
OV
NES = - 1.66
P- value = 0.055
Enrichment plot: HALLMARK_ESTROGEN_RESPONSE_EARLY
Enrichment score (ES)
High R2
Low R2
UCEC
NES = -2.17
P- value = 0.005
Enrichment plot: HALLMARK_ESTROGEN_RESPONSE_EARLY
Enrichment score (ES)
High R2
Low R2
NES = -1.99
P- value = 0.000
Enrichment plot: HALLMARK_ESTROGEN_RESPONSE_LATE
Enrichment score (ES)
High R2
Low R2
NES = -1.59
P- value = 0.098
Enrichment plot: HALLMARK_ESTROGEN_RESPONSE_LATE
Enrichment score (ES)
High R2
Low R2
NES = -2.21
P- value = 0.005
Enrichment plot: HALLMARK_ESTROGEN_RESPONSE_LATE
Enrichment score (ES)
High R2
Low R2

Supplementary Figure 20. Expression of genes included in estrogen response gene sets from breast, ovarian, and uterine cancers.

a Heatmap for mRNA expression of genes included in estrogen early and late response gene sets. Each column stands for an individual cancer sample, and each row denotes a gene. Among genes in estrogen early and late response gene sets, 29 genes are commonly enriched in BRCA, OV, and UCEC. Z-scores (standard deviation) of 29 genes and m6A reader genes are stratified in 6 colors (-3 to 3). At the top of heatmap, R2 signature is presented and discriminated into high and low groups by the color (red: high, blue: low). From left to right, breast cancer samples are sorted by R2 signatures in ascending order. **b** GSEA plot of estrogen early and late response gene sets in BRCA, OV, and UCEC according to R2 signature. Each tumor type's normalized enrichment score and P-value is demonstrated.

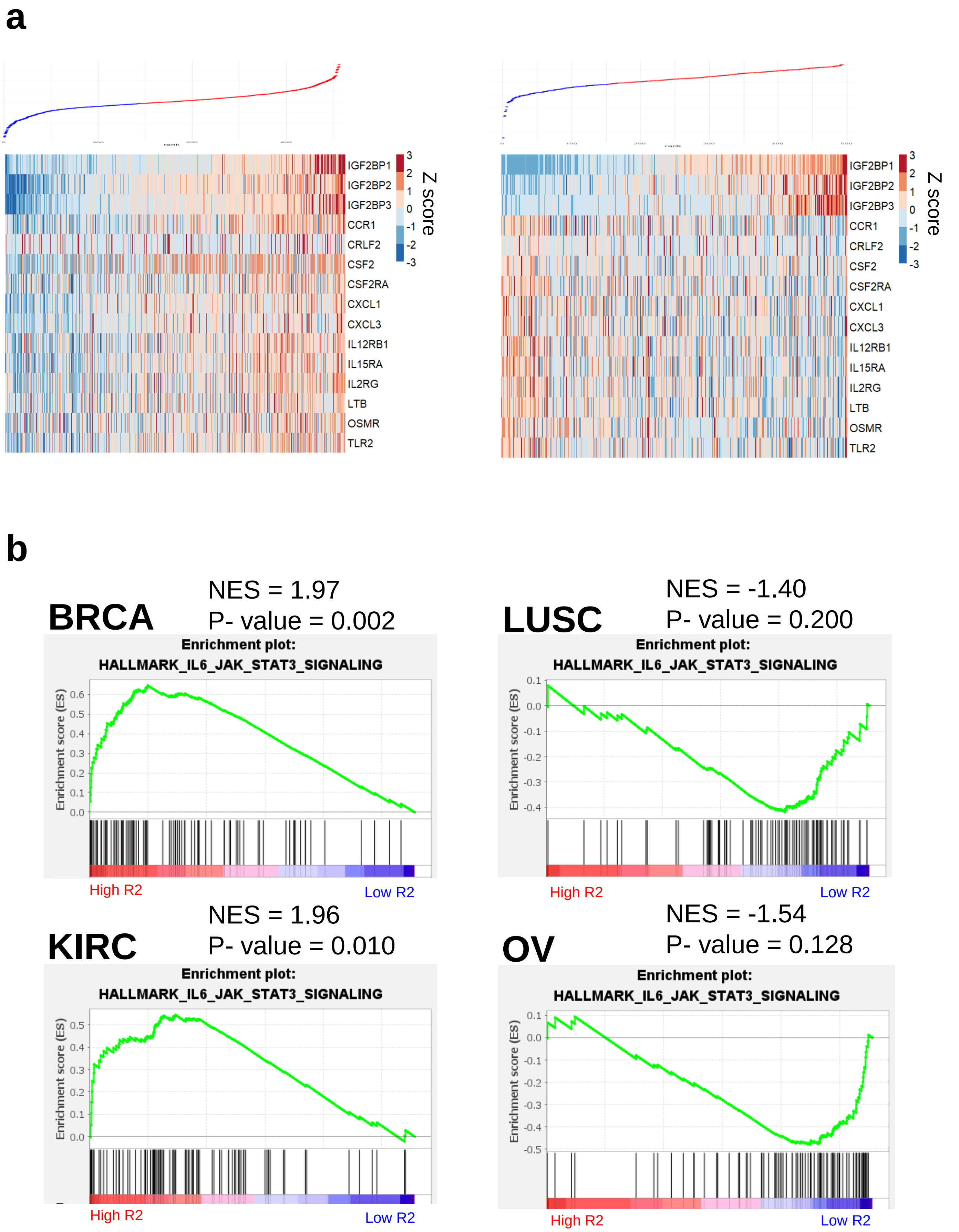

a
IGF2BP1
IGF2BP2
IGF2BP3
CCR1
CRLF2
CSF2
CSF2RA
CXCL1
CXCL3
IL12RB1
IL15RA
IL2RG
LTB
OSMR
TLR2
Z score
3
2
1
0
-1
-2
-3
b
BRCA
NES = 1.97
P- value = 0.002
Enrichment plot:
HALLMARK_IL6_JAK_STAT3_SIGNALING
Enrichment score (ES)
High R2
Low R2
LUSC
NES = -1.40
P- value = 0.200
Enrichment plot:
HALLMARK_IL6_JAK_STAT3_SIGNALING
Enrichment score (ES)
High R2
Low R2
KIRC
NES = 1.96
P- value = 0.010
Enrichment plot:
HALLMARK_IL6_JAK_STAT3_SIGNALING
Enrichment score (ES)
High R2
Low R2
OV
NES = -1.54
P- value = 0.128
Enrichment plot:
HALLMARK_IL6_JAK_STAT3_SIGNALING
Enrichment score (ES)
High R2
Low R2

Supplementary Figure 21. Expression of genes included in IL6-JAK-STAT3 signaling gene sets from breast, kidney, lung, and ovarian cancer.

a Heatmap for mRNA expression of genes included in IL6-JAK-STAT3 signaling gene sets. Each column stands for an individual cancer sample, and each row denotes a gene. Among genes in IL6-JAK-STAT3 signaling gene sets, 12 genes are commonly enriched in high R2 signature groups of BRCA and KIRC (left panel) and in low R2 signature groups of LUSC and OV (right panel). Z-scores (standard deviation) of 12 genes and IGF2BPs are stratified in 6 colors (-3 to 3). At the top of heatmap, R2 signature is presented and discriminated into high and low groups by the color (red: high, blue: low). From left to right, cancer samples are sorted by R2 signatures in ascending order. **b** GSEA plot IL6-JAK-STAT3 signaling gene sets according to R2 signature in BRCA, KIRC, LUSC, and OV. Each tumor type's normalized enrichment score and P-value is demonstrated.

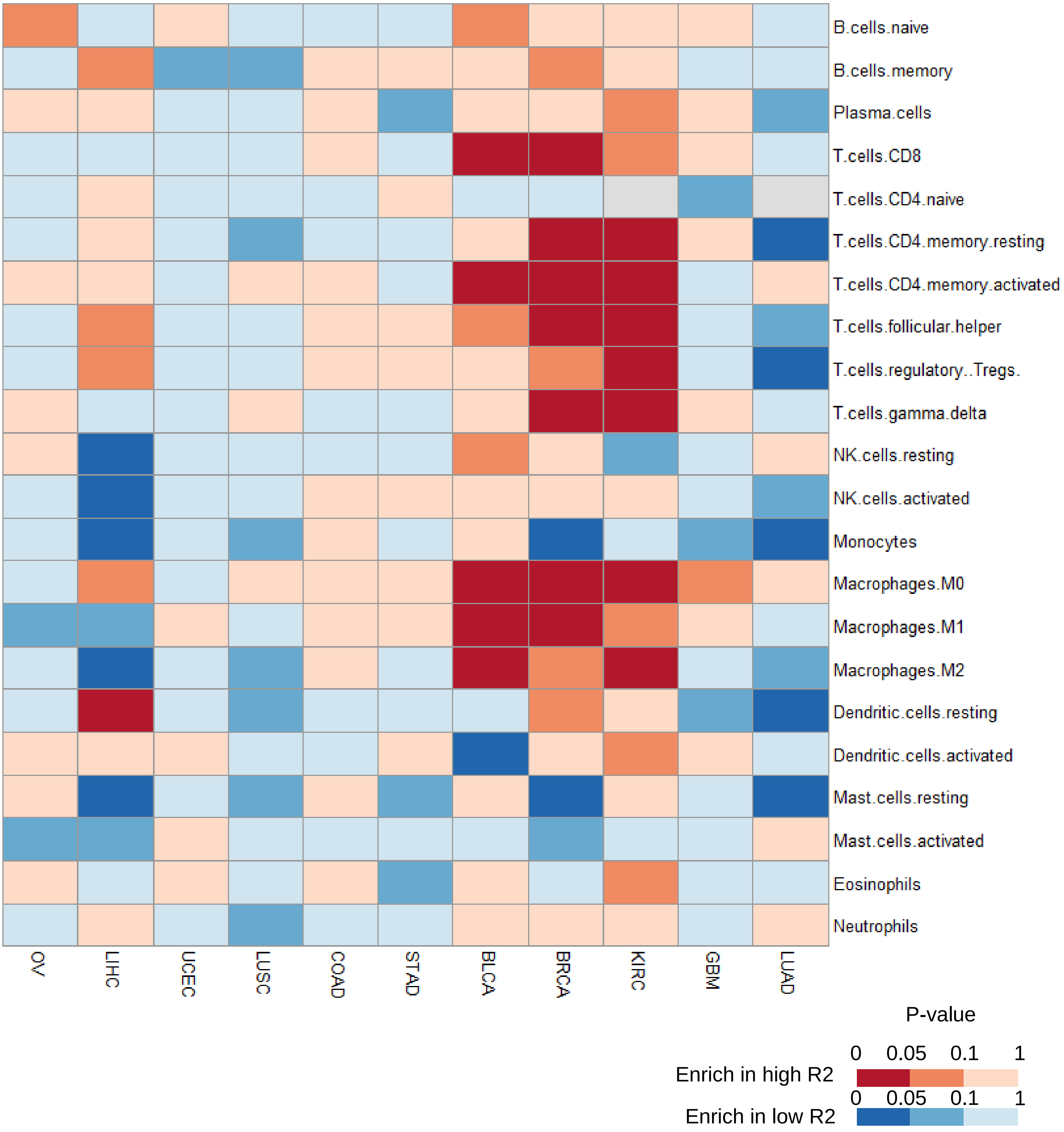

Supplementary Figure 22. Analyzing immune cell proportion in tumors by CIBERSORT.

Heatmap for the difference of immune cell count (absolute mode of CIBERSORT) between samples with high and low m6A R2 signature. Each column stands for an individual cancer type, and each row denotes an immune cell type. Each cell represents -log(P-value) from student t-test. Red cells mean that high R2 signature has higher count than low R2 signature, and blue cells mean that high R2 signature has lower count than low R2 signature.

a

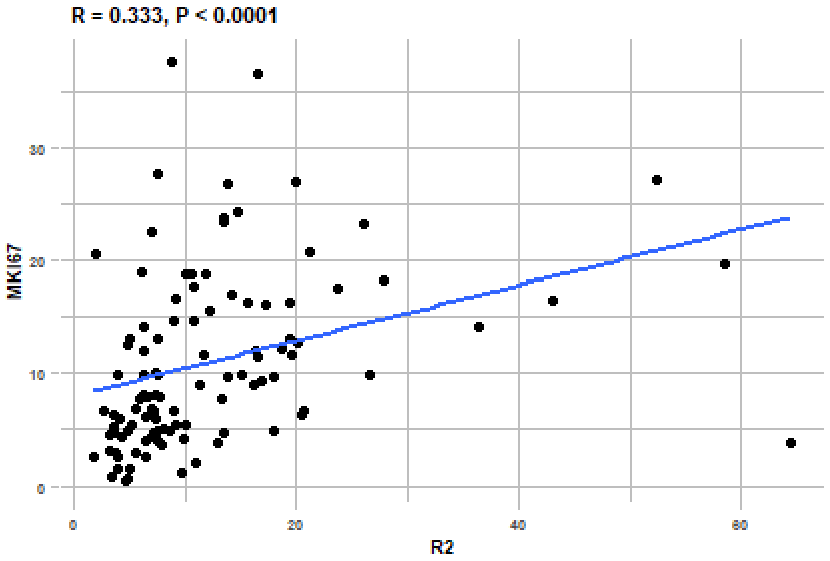

b

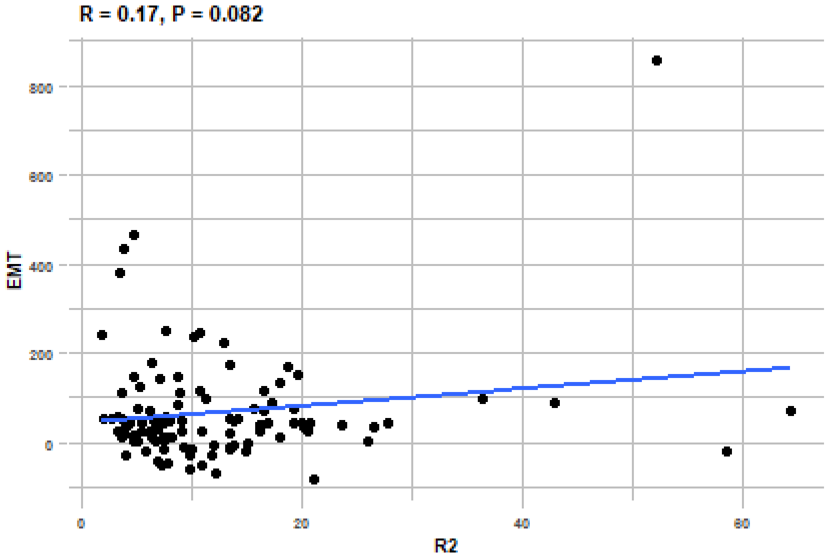

Supplementary Figure 23. Scatter plot of m6A R2 signature with proliferation and EMT markers in in-house gastric cancer cohort.

Scatter plots show the correlation of m6A R2 signatures with MKI67 expression **(a)** and EMT signature **(b)** in RNA sequencing data of in-house gastric cancer cohort. EMT signature was calculated by arithmetic means of EMT marker genes (CDH2, FN1, and VIM). Each plot contains Pearson correlation coefficients (R) and p-value (P).

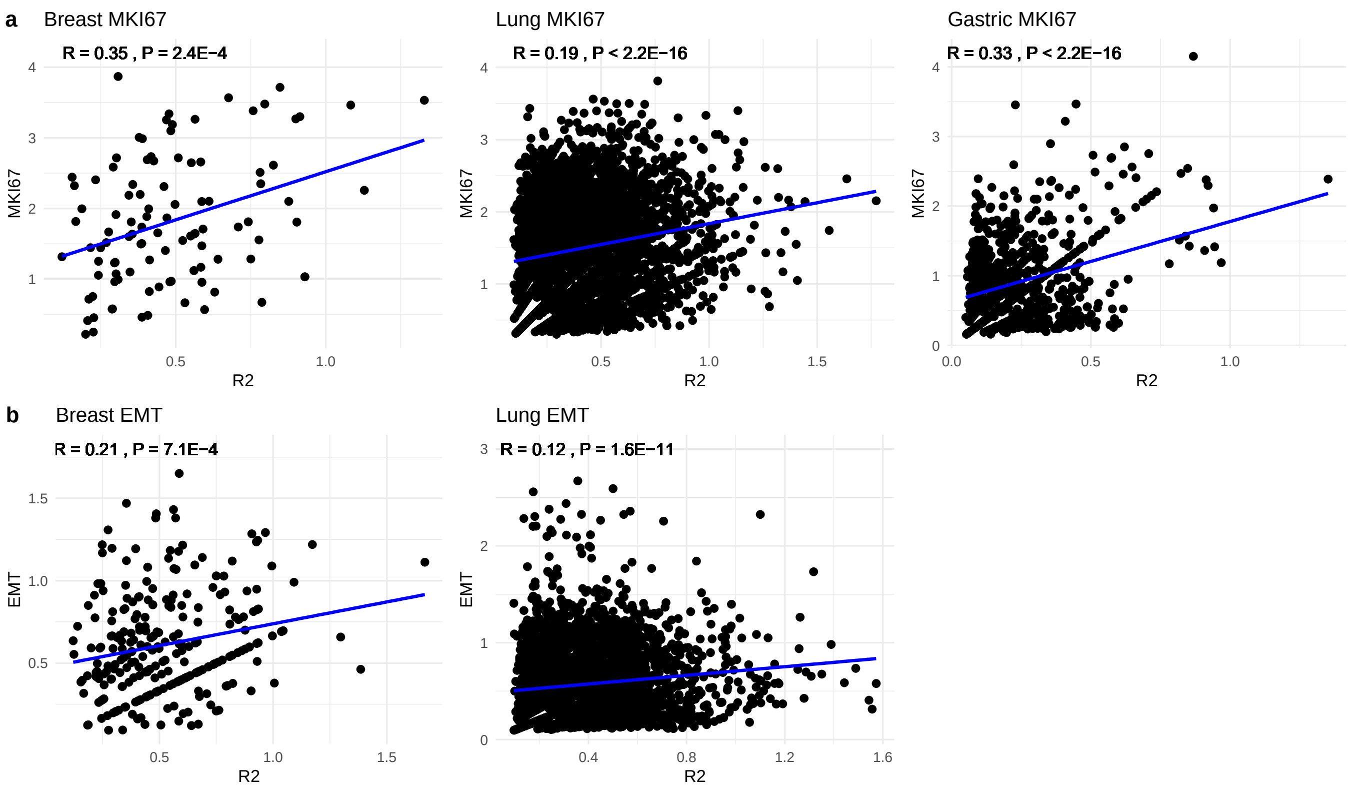

Supplementary Figure 24. Scatter plot of m6A R2 signature with EMT & proliferation signatures.

Scatter plots show the correlation of m6A R2 signatures with MKI67 expression **(a)** and EMT signature **(b)** in tumor cells in scRNA data of breast, lung, and gastric cancers. EMT signature was calculated by arithmetic means of EMT marker genes (CDH2, FN1, and VIM). Each plot contains Pearson correlation coefficients (R) and p-value (P).

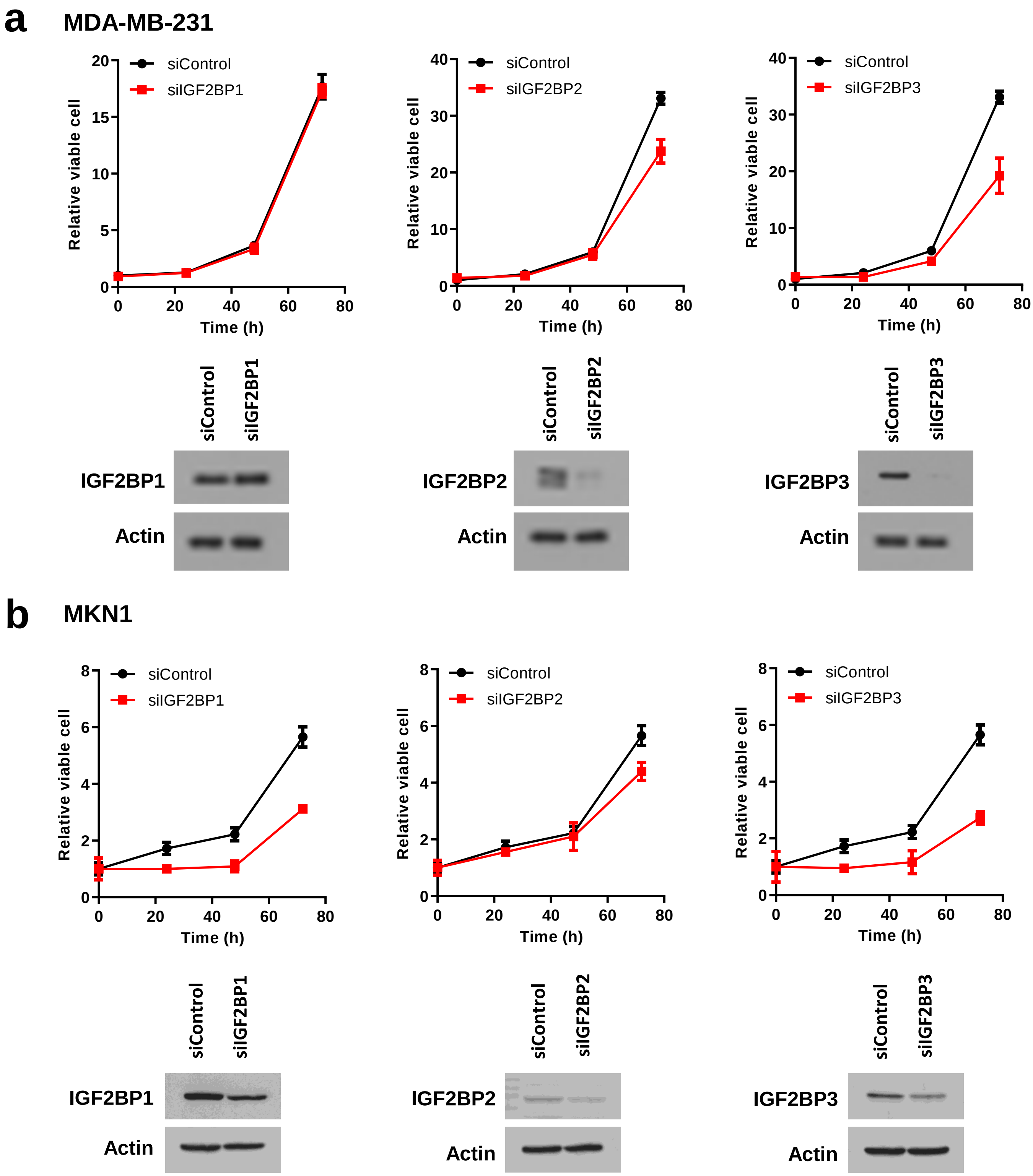

Supplementary Figure 25. Knock-down effect of IGF2BPs on cell proliferation.

Cell proliferation was determined using WST assays after knock-down of IGF2BPs with siRNAs for 48 h in MDA-MB-231 (**a**) and MKN1 cells (**b**). Upper panels show plots of cell proliferation and lower panels demonstrate the knock-down of IGF2BPs with siRNA. Data are presented as the mean values ± standard error of mean (SEM) of experiments performed in triplicate.

Supplementary Table 1. RNA m6A signature-related genes in cell cluster marker genes

Cancer Type	Gene	Cell Type	Average log_2FC*	Adj. pvalue	Pct.1**	Pct.2***
Breast	METTL3 (W)	Tumor Cell	0.29	2e-21	0.17	0.06
	KIAA1429 (W)	Tumor Cell	0.50	3e-172	0.45	0.18
		Tumor Cell	0.49	4e-136	0.46	0.18
		Tumor Cell	0.42	5e-17	0.35	0.19
		Tumor Cell	0.29	1e-26	0.70	0.19
	ZC3H13 (W)	T Cell	0.27	3e-13	0.27	0.45
		Tumor Cell	0.57	3e-28	0.65	0.43
	FTO (E)	Tumor Cell	0.72	4e-181	0.26	0.03
	YTHDF1 (R)	Tumor Cell	0.72	<4e-202	0.37	0.06
		Tumor Cell	0.46	1e-5	0.21	0.08
		Macrophage	0.50	3e-16	0.30	0.08
		Fibroblast	0.43	5e-50	0.29	0.09
	YTHDF2 (R)	Tumor Cell	0.35	8e-120	0.49	0.28
		Tumor Cell	0.26	9e-36	0.48	0.29
	IGF2BP2 (R)	Tumor Cell	0.26	1e-115	0.11	0.01
	IGF2BP3 (R)	Tumor Cell	0.37	<4e-202	0.16	0.01
Lung	WTAP (W)	Neutrophil	0.35	8e-5	0.12	0.17
	ZC3H13 (W)	T cell	0.36	1e-4	0.31	0.43
	YTHDF2 (R)	Tumor Cell	0.44	9e-35	0.68	0.28
		Tumor Cell	0.32	<4e-202	0.51	0.26

	IGF2BP2 (R)	Neutrophil	0.34	4e-202	0.27	0.14
Gastric	WTAP (W)	B Cell	0.34	1e-12	0.36	0.35
		Macrophage	1.00	<4e-202	0.56	0.35
	YTHDF2 (R)	T Cell	0.29	2e-6	0.26	0.27
	YTHDF3 (R)	Macrophage	0.49	1e-119	0.28	0.15
	IGF2BP2 (R)	Tumor	0.37	<4e-202	0.37	0.05
		Pit Mucous Cell	0.32	<4e-202	0.13	0.05

P-values were adjusted using Benjamini-Hochberg method.

*Average log_2FC: Average fold-change of markers in this cluster compared to the rest of the clusters in log_2 ratio

** Pct.1: Percentage of cells expressing the marker in this cluster

*** Pct.2: Average percentage of cells expressing the marker in rest of the clusters
